# *Bacillus* sp. G2112 Detoxifies Phenazine-1-carboxylic Acid by *N*5 Glucosylation

**DOI:** 10.3390/molecules29030589

**Published:** 2024-01-25

**Authors:** Kenechukwu Iloabuchi, Dieter Spiteller

**Affiliations:** 1 Department Chemical Ecology/Biological Chemistry, University of Konstanz, Universitätsstraße 10, 78457 Konstanz, Germany; kenechukwu.iloabuchi@uni-konstanz.de; 2 Department of Biochemistry, Faculty of Biological Sciences, University of Nigeria Nsukka, Obukpa Road, Nsukka 410105, Nigeria

**Keywords:** antibiotic, biocontrol, co-cultivation, epiphyte, glycosylation, mass spectrometry, natural product, nuclear magnetic resonance, resistance

## Abstract

Microbial symbionts of plants constitute promising sources of biocontrol organisms to fight plant pathogens. *Bacillus* sp. G2112 and *Pseudomonas* sp. G124 isolated from cucumber (*Cucumis sativus*) leaves inhibited the plant pathogens *Erwinia* and *Fusarium*. When *Bacillus* sp. G2112 and *Pseudomonas* sp. G124 were co-cultivated, a red halo appeared around *Bacillus* sp. G2112 colonies. Metabolite profiling using liquid chromatography coupled to UV and mass spectrometry revealed that the antibiotic phenazine-1-carboxylic acid (PCA) released by *Pseudomonas* sp. G124 was transformed by *Bacillus* sp. G2112 to red pigments. In the presence of PCA (>40 µg/mL), *Bacillus* sp. G2112 could not grow. However, already-grown *Bacillus* sp. G2112 (OD_600_ > 1.0) survived PCA treatment, converting it to red pigments. These pigments were purified by reverse-phase chromatography, and identified by high-resolution mass spectrometry, NMR, and chemical degradation as unprecedented 5*N*-glucosylated phenazine derivatives: 7-imino-5*N*-(1′β-D-glucopyranosyl)-5,7-dihydrophenazine-1-carboxylic acid and 3-imino-5*N*-(1′β-D-glucopyranosyl)-3,5-dihydrophenazine-1-carboxylic acid. 3-imino-5*N*-(1′β-D-glucopyranosyl)-3,5-dihydrophenazine-1-carboxylic acid did not inhibit *Bacillus* sp. G2112, proving that the observed modification constitutes a resistance mechanism. The coexistence of microorganisms—especially under natural/field conditions—calls for such adaptations, such as PCA inactivation, but these can weaken the potential of the producing organism against pathogens and should be considered during the development of biocontrol strategies.

## 1. Introduction

Around 25% of crops are lost each year because of plant pathogens [1]. Moreover, environmental pollution, fast evolving resistances to established treatments, the growing human population, and increasing demand for pesticide-free food call for sustainable ways of phytopathogen control [2]. Microbial symbionts beneficial to plant protection, usually referred to as biocontrol organisms (BCOs), can effectively control plant pathogens and support plant growth, thereby ensuring global food supply and reducing the use of environmentally hazardous pesticides [3,4,5,6,7]. Understanding the complex microbial interactions in the soil/plant ecosystem where BCOs function will aid their proper formulation and application [2,8,9,10,11].

BCOs of the genera *Bacillus* and *Pseudomonas* have been intensively investigated because they are ubiquitous, often co-isolated, and employ a variety of strategies to fight pathogens and boost systemic immunity in plants. *Pseudomonas* spp. massively colonize root nodules, thereby limiting access for pathogens [12]. Moreover, they release antibiotics that act against pathogens, and use siderophores to acquire scarce minerals, such as iron ions, thus outcompeting pathogens [13,14]. In addition, pseudomonads prime the plants’ defense mechanisms against pathogens and pests [15].

Bacilli employ similar mechanisms as pseudomonads: the efficient colonization of plants by biofilm formation limits nutrient and space availability to pathogens [16]; release of antibiotics and degrading enzymes destroys pathogens [17,18]; release of plant hormone analogs promotes plant growth [19]; and induction of systemic resistance in plants [20]. Moreover, *Pseudomonas* spp. and *Bacillus* spp. can be cultivated on an industrial scale and survive in nutrient-poor soils, which renders them excellent candidates for biocontrol [12,14,17,20]

Though often coexisting, the interactions of *Bacillus* and *Pseudomonas* spp. are rather complex, involving antibiosis [21,22], competition [23], antagonism [24,25,26,27], and amensalism [16,28,29,30]. A detailed study of the interactions between *Pseudomonas chlororaphis* PCL1606 and *Bacillus amyloliquefaciens* FZB42 revealed that both organisms continuously released antagonizing compounds against each other to ensure coexistence [22]. Conversely, synergistic interactions boosting biocontrol potentials of microbial partners and plant growth have been characterized [31,32,33,34,35].

Our understanding of the interactions of bacilli and pseudomonads has increased in recent years, thanks to studies of model microbial communities that reveal interactions between multiple organisms [30,36,37,38], and sensitive detection techniques that allow the in situ characterization of interactions between microorganisms [22,23,39]. Co-cultivation experiments of two microbial partners are important to pinpoint specific interactions and to resolve details of complex interactions [22,23,25].

During the co-cultivation of bacterial isolates from cucumber (*Cucumis sativus*) on agar plates in order to test whether there is a synergistic effect against phytopathogens, we observed the release of red pigments into the agar medium when two isolates, *Bacillus* sp. G2112 and *Pseudomonas* sp. G124, interacted, but not when they grew alone.

Here, we present why the red pigments were formed in the co-culture of *Bacillus* sp. G2112 and *Pseudomonas* sp. G124, elucidate the structure of the pigments, and discuss the implications of such reactions caused upon the interaction of microorganisms for the biocontrol of pathogens.

## 2. Results

### 2.1. Co-Cultivation of Bacillus sp. G2112 and Pseudomonas sp. G124

*Bacillus* sp. G2112 and *Pseudomonas* sp. G124 were isolated from cucumber plants. Both microorganisms are closely related to biocontrol strains (Supplementary Figures S1 and S2) of their respective genera. *Bacillus* sp. G2112 and *Pseudomonas* sp. G124 inhibited the plant pathogens *Fusarium equiseti* and *Erwinia tracheiphila* (Figures S3 and S4).

**Figure 1:**
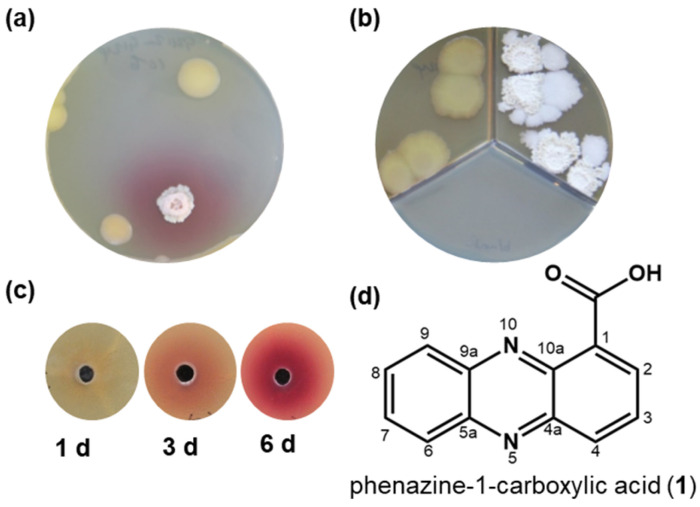
Red pigment formation by *Bacillus* sp. G2112 in response to *Pseudomonas* sp. G124. (**a**) Co-culture of *Bacillus* sp. G2112 and *Pseudomonas* sp. G124. Red pigmentation occurred around *Bacillus* sp. G2112. (**b**) Co-cultivation of *Bacillus* sp. G2112 and *Pseudomonas* sp. G124 in different compartments separated by a plastic barrier allowing only exchange via the headspace did not result in red pigment formation. (**c**) Red pigment formation by *Bacillus* sp. G2112 treated with methanol extracts of 1, 3, and 6 d old *Pseudomonas* sp. G124 spent agar media. (**d**) Structure of the antibiotic phenazine-1-carboxylic acid (**1**) produced by *Pseudomonas* sp. G124, which caused red pigment formation by *Bacillus* sp. G2112.

When *Bacillus* sp. G2112 and *Pseudomonas* sp. G124 were co-cultured on agar plates, a red pigment occurred in the agar. The red pigment was absent when each organism was cultivated alone. (Figure 1a and Figure S5). The red color appeared in all three agar media tested, namely soy flour mannitol medium [40], King’s B medium [41], and modified 5b glycerol salt medium [42,43]. Eliminating Fe^2+^ and Mn^2+^ ions or replacing complex nitrogen sources such as peptone with amino acids did not stop red pigment formation as long as both organisms survived the cultivation conditions. However, when *Bacillus* sp. G2112 and *Pseudomonas* sp. G124 colonies were separated by a plastic barrier in compartmentalized Petri dishes that allowed only an exchange of volatiles via the headspace, the red pigment was not observed (Figure 1b and Figure S5). Thus, red color formation was not caused by a volatile compound but by a compound released into the agar medium.

*Bacillus* sp. G2112 reacted to a compound secreted by the neighboring *Pseudomonas* sp. G124 (Figure 1a) because the red pigmentation appeared around the *Bacillus* sp. G2112 colonies. The closer the colonies of *Bacillus* sp. G2112 and *Pseudomonas* sp. G124 were, the faster the red color appeared (Figure S6).

Other bacilli that were co-cultivated with *Pseudomonas* sp. G124 produced the red pigment on agar plates as well, although with varying intensities (Table S1). Conversely, no red pigment formation was observed when *Bacillus* sp. G2112 was grown together with *Pseudomonas syringae* pv. *syringae* 22d/93, *Pseudomonas syringae* pv. *glycinea* 1a/96, or *Pseudomonas fluorescens* DSM6506 (Table S1), indicating that *Pseudomonas* sp. G124 released a specific compound that causes red pigment formation around *Bacillus* sp. G2112 colonies.

### 2.2. Identification of the Antibiotic Phenazine-1-carboxylic Acid as Precursor of Red Pigments

Supernatants and methanol extracts of 5b glycerol agar medium on which *Pseudomonas* sp. G124 was cultivated for 1, 3, and 6 d were tested to see whether they caused red pigment formation by *Bacillus* sp. G2112. Red pigment production increased with age of the *Pseudomonas* sp. G124 spent culture, indicating that more pigment-inducing compound was released into the medium with time (Figure 1b).

In order to identify which compound caused the red pigment formation by *Bacillus* sp. G2112, the metabolite profiles of methanol extracts of 5b glycerol agar from *Pseudomonas* sp. G124 and *P. syringae* pv. *syringae* 22d/93 (the latter did not cause red pigment formation) were compared by LC–UV–MS profiling. Several ESI–MS peaks were only present in either *Pseudomonas* strain (Figure S7).

When testing HPLC-purified candidate compounds from *Pseudomonas* sp. G124 against *Bacillus* sp. G2112 for the red pigment formation, we identified the responsible compound. It exhibited a UV spectrum with λ_max_ at 223, 249, and 370 nm as well as a quasimolecular ion [M+H]^+^ at *m*/*z* 225 (Figures S8 and S9). The compound was extracted into ethyl acetate at a low pH (<4) but could be re-extracted into water at pH > 9. Further precipitation into chloroform allowed its straightforward purification (ca. 150 mg, >90% purity per liter of 5b glycerol agar medium).

High resolution mass spectrometry produced a quasimolecular ion of [M + H]^+^ at *m*/*z* 225.06598 corresponding to the molecular composition C_13_H_9_N_2_O_2_ (calc. *m*/*z* 225.06585, Δppm 0.56) (Figure S9). The compound contained 11 double bond equivalents suggesting an aromatic system. Tandem MS indicated the loss of water (*m*/*z* 207) and CO_2_ (*m*/*z* 181) suggesting the presence of a carboxyl group (Figure S9). The NMR data verified the molecular formula and the presence of a carboxyl group. The aromatic heterocyclic molecule comprised 1,2-substituted and 1,2,3-substituted benzene rings joined by two nitrogen bridges (Figures S10–S14). Taking the analytical data together (Figures S8–S14), the compound was identified as phenazine-1-carboxylic acid (PCA, **1**), which is a well-known antibiotic produced by several *Pseudomonas* and *Streptomyces* strains [44,45,46].

Depending on the PCA (**1**) concentration and application time, it inhibited the growth of *Bacillus* sp. G2112 or caused red pigment production in agar diffusion assays (Figure S15). The germination of *Bacillus* sp. G2112 was completely inhibited in media supplemented with 180 µM (40 µg/mL) PCA (**1**) (Figure S15a). The hampered growth of *Bacillus* sp. G2112 on PCA-supplemented agar reflected by a reduced colony diameter was observed at PCA (**1**) concentrations as low as 1.8 µM (Figure S15a). However, already-growing *Bacillus* sp. G2112 cells survived PCA (**1**) treatment by producing red pigments. The color intensified with increasing PCA (**1**) concentration (0.004, 0.008, 0.016, and 0.024 mg/mL) (Figure S15b).

### 2.3. Identification of the Red Pigments

**Figure 2:**
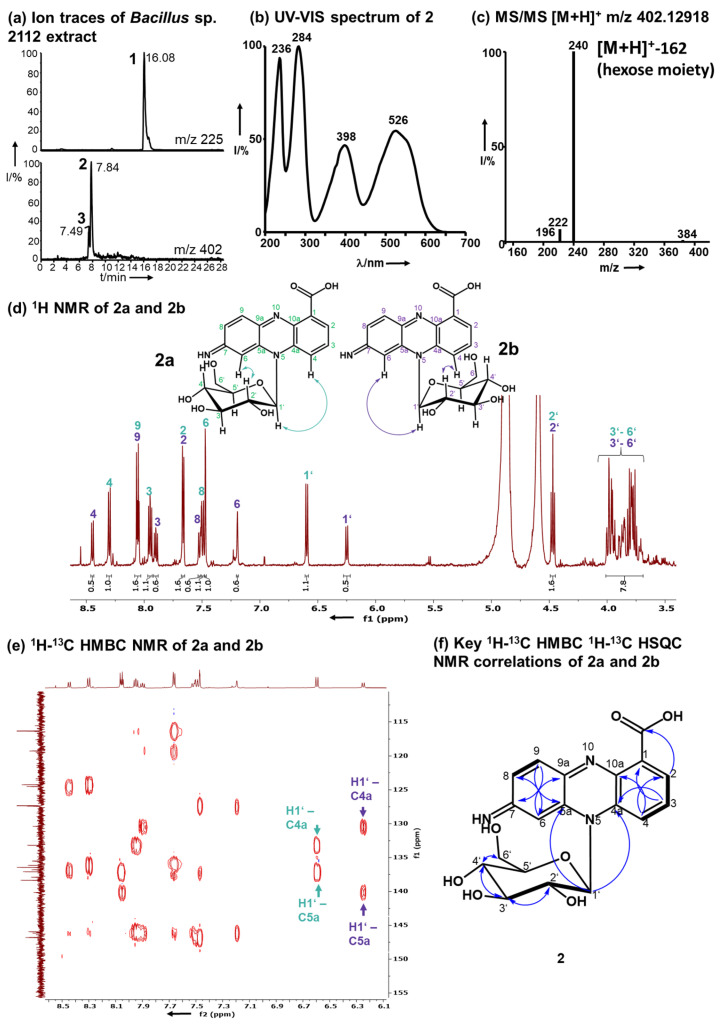
Structure elucidation of **2** as 7-imino-5*N*-(1′β-D-glucopyranosyl)-5,7-dihydrophenazine -1-carboxylic acid. (**a**) Ion traces *m*/*z* 225 and 402 (**1**, **2,** and **3**) of *Bacillus* sp. G2112 supernatant after exposure to PCA (**1**). (**b**) UV–Vis spectrum of **2**. (**c**) MS/MS ESI–mass spectrum of **2** indicating the loss of a hexose moiety. (**d**) ^1^H-NMR (600 MHz, CD_3_OD) of **2** with a double dataset corresponding to two isomers **2a** (green) and **2b** (purple; arrows in the structures indicate NOE correlations, Figures S47 and S48). (**e**) ^1^H-^13^C-HMBC revealing the correlations of the anomeric protons H1’ of **2a** and **2b** to the phenazine ring system ^13^C carbons **4a** and **5a**. (**f**) Key ^1^H-^13^C-HMBC correlations are indicated by blue arrows and ^1^H-^1^H-COSY by thick bonds.

A time course assay of *Bacillus* sp. G2112 in 5b glycerol liquid medium challenged with 134 µM PCA (**1**) revealed that the culture turned red at around 3 d post-treatment (Figure S15c). A comparison of LC–MS profiles of supernatants of PCA-(**1**)-treated and untreated *Bacillus* sp. G2112 cultures over a 6-d period revealed that the PCA (**1**) decreased over time (Figure S16) while red compounds **2** and **3** increased. Both pigments were eluted at 7.5 and 7.9 min with *m*/*z* 402 and λ_max_ 236, 284, 398, 526 nm/λ_max_ 236, 288, 398, 532 nm, respectively (Figure 2a,b, Figures S17, S18, S27 and S28).

To elucidate the structures of the red pigments **2** and **3**, *Bacillus* sp. G2112 growing in liquid 5b glycerol medium (5.25 L, OD_600_ 0.3) was treated with PCA (**1**, 0.36 mM). *Bacillus* sp. G2112 survived such high amounts of PCA (**1**, 0.36 mM) if it was administered in non-lethal doses by pulse feeding (0.12 mM, three times) to already-grown cultures. The pigments were purified, avoiding heat and light, by freeze-drying the spent 5b glycerol medium to one-fifth of the original volume and subjecting the concentrate to Diaion HP20 column chromatography. Fractions containing **2** and **3** were freeze-dried and separated by RP8 MPLC to obtain a mixture of **2** and **3** at 8–10 min, which was resolved using a polar RP HPLC column at 14–15 min and 16–18 min, respectively.

During purification, we observed that pigments **2** and **3**, which were very polar and could not be extracted with organic solvents such as ethyl acetate or chloroform, decomposed upon extended (>1 h) exposure to intense light or heat, e.g., in centrifuge dryers, and after acidification (<pH 4) or alkalization (>pH 9).

High-resolution electrospray ionization mass spectrometry (HR-ESI-MS) revealed quasimolecular ions [M+H]^+^ at *m*/*z* 402.12918 and 402.12948 for **2** and **3** respectively, corresponding to the molecular composition C_19_H_20_N_3_O_7_ (calc. *m*/*z* 402.12958, Δppm 0.99 and Δppm 0.24, respectively, Figures S17 and S27). Pigments **2** and **3** exhibited similar MS/MS fragmentation spectra (Figure 2c, Figures S19 and S29), dominated by the fragments with *m*/*z* 240.07664 for **2** and 240.07686 for **3** resulting from the loss of a hexose residue (C_6_H_10_O_5_, 162 amu). The aglycons had a molecular composition C_13_H_10_N_3_O_2_ and 10.5 double bond equivalents similar to PCA (**1**, C_13_H_8_N_2_O_2_). Mass spectrometry suggested that **2** and **3** constitute glycosylated aminophenazine-1-carboxylic acids.

^1^H-NMR (600 MHz, CD_3_OD) analyses revealed that **2** and **3** contained six aromatic CH protons and seven HC-OH protons that are typical for glycosyl moieties (Figure 2d, Figures S20 and S30). In the ^1^H-NMR of both pigments **2** and **3,** a double dataset in a ratio of 2:1 was observed indicating the presence of two isomers, namely, **2a** and **2b,** as well as **3a** and **3b**. The observed protons at 6.25–6.60 ppm with ^13^C carbon signals at 91 ppm are characteristic for anomeric protons and carbons of sugars in the vicinity of an aromatic system [47,48,49]. These anomeric proton signals all had a coupling constant of 9.3 Hz, which is characteristic for β-anomers [48]. The overlap of the signals of the other HC-OH protons of the sugar moiety hindered a clear assignment of the hexose moiety (3.75–4.48 ppm, H2’–H6’). Therefore, the hexose moiety of **2** and **3** was identified by methanolysis followed by acetylation and GC–MS analysis in comparison to standards to be glucose (Figure S45). Moreover, after hydrolysis of **2** the obtained sugar was derivatized using chiral L-cysteine methyl ester [50] and 4-fluoro-benzyl isothiocyanate. The glucosyl moiety of **2** is D-glucopyranose (Figure S46).

The aglycon of **2** contains six aromatic protons between δ_H_ 7.35–8.38 ppm. ^1^H-NMR and ^1^H,^1^H-COSY analyses revealed two benzene ring systems, a 1,2,4-substituted and a 1,2,3-substituted aromatic moiety (Figure 2d, Figures S20, S22 and S23), which result from the functionalization of position 7 of PCA (**1**). The positions of the aromatic protons were confirmed by ^1^H-^1^H COSY (Figures S22 and S23).

Pigment **3** differs from **2** by having a different substitution pattern of the phenazine-1-carboxylic acid core that is reflected by signals in the aromatic region corresponding to a 1,2-substituted and a 1,2,3,5-substituted benzene moiety as deduced from ^1^H-NMR and ^1^H,^1^H-COSY (Figures S30–S32). In **3,** the 3 position of PCA (**1**) is functionalized by *Bacillus* sp. G2112.

The positions of H2 and H4 in **2** and H6 in **3** were established by ^1^H-^13^C HMBC: H2 and H4 correlated to C4 and C2 respectively in **2,** and H8 correlated to C6 in **3** (Figures S25 and S34). The HMBC correlations between H2 and the carboxylic acid carbon in both **2** and **3** verified the position of H2.

Analysis of the ^13^C-NMR, HSQC and HMBC NMR spectra indicated that the position 7 of **2** and position 3 of **3** are functionalized by an imino group, respectively (Figures S24, S25, S33 and S34). For **2,** the imino group was confirmed by HMBC correlations of H6 and H9 to carbon C7 at 162 ppm (Figure S25). This shift is typical for imino groups [51]. The imino moiety in **3** was assigned by analogy to **2** because there are no HMBC correlations to C3 in compound **3**.

The connection of the glucosyl moiety to the phenazine aglycon was established by analysis of the HMBC correlations: the anomeric H1’ correlated to C4a and C5a in both **2** and **3**, proving that the sugar moiety is attached to *N*5 (Figure 2e, Figures S25 and S34).

**Figure 3:**
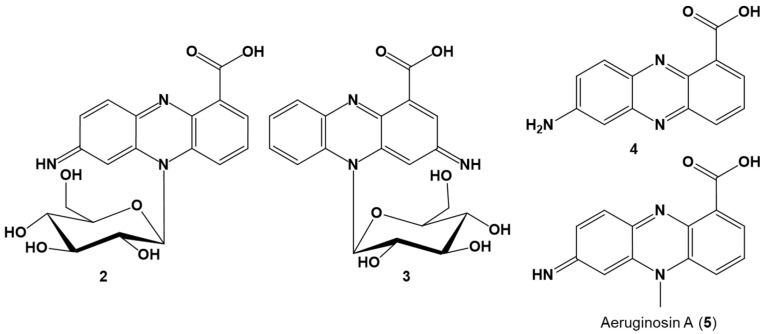
Structures of the red pigments, 7-imino-5*N*-(1′β-D-glucopyranosyl)-5,7-dihydrophenazine-1-carboxylic acid (**2**) and 3-imino-5*N*-(1′β-D-glucopyranosyl)-3,5-dihydrophenazine-1-carboxylic acid (**3**), detoxification products of *Bacillus* sp. G2112 upon challenge with phenazine-1-carboxylic acid (**1**) and the degradation product 7-amino-phenazine-1-carboxylic acid (**4**). For comparison, the related aeruginosin A (**5**) from *Pseudomonas aeruginosa* [52,53] is depicted, though it was neither isolated from *Bacillus* sp. G2112 nor *Pseudomonas* sp. G124.

Thus, based on the analytical data, **2** was identified as 7-imino-5*N*-(1′-β-D-glucopyranosyl)-5,7-dihydrophenazine-1-carboxylic acid and **3** as 3-imino-5*N*-(1′-β-D-glucopyranosyl)-3,5-dihydrophenazine-1-carboxylic acid (Figure 3).

The *N*5 atom of **2** and **3** is chiral because of its lone electron pair and the *N*5-glucosidic bond constitutes a chiral axis as observed by the occurrence of a double NMR dataset for both **2** and **3**, corresponding to two atropisomers: **2a** and **2b**, as well as **3a** and **3b**, respectively. The analysis of the NOESY NMR spectra revealed the orientations of the atropisiomers (Figure 2, Figures S47, S48, S49 and S50). All ^1^H-NMR and ^13^C-NMR assignments for **2** and **3** are summarized in Tables S2 and S3, respectively.

Because of the lability of **2** and **3**, initial purification attempts in the presence of light and acid led to hydrolysis of the red pigments yielding **4** with a quasimolecular ion [M + H]^+^ at *m*/*z* 240.07655 (Δ 0.85 ppm, C_13_H_10_O_2_N_3_), corresponding to 7-aminophenazine-1-carboxylic acid (**4**). The decomposition product was identical to the aglycon obtained from hydrolysis of pure **2**. UV, MS, and NMR data of **4** are provided (Figures S37–S44 and Table S4).

### 2.4. Detoxification Products Did Not Inhibit Bacillus sp. G2112

Paper disc agar diffusion assays against *Bacillus* sp. G2112 revealed that the glucosylated metabolite **3** was not toxic compared to PCA (**1**) at similar concentrations (20 mM, 20 µL), indicating that **2** and **3** are detoxification products (Figure S51).

## 3. Discussion

*Pseudomonas* sp. G124 and *Bacillus* sp. G2112, both isolated from cucumber plants, inhibited cucumber pathogens, namely *Erwinia* and *Fusarium*, demonstrating their potential as biocontrol organisms. Because both organisms originated from the same habitat, we investigated how they interacted with each other in co-cultivation experiments on agar plates, initially with the aim to use them in combination against plant pathogens. *Pseudomonas* sp. G124 and *Bacillus* sp. G2112 coexisted well on agar plates, except that a red pigmentation surrounded *Bacillus* sp. G2112 colonies only when it was grown in co-culture. *Pseudomonas* sp. G124 releases the antibiotic phenazine-1-carboxylic acid (**1**) into the medium, which inhibited *Bacillus* sp. G2112 in its early growth phase.

*Pseudomonas* strains are well known to produce phenazines, such as PCA (**1**), which can act as antibiotics, modulate gene expression, biofilm formation, and mineral cycling, or induce systemic resistance in host plants [14,54]. More complex phenazines are usually produced by *Actinobacteria* including glycosylated, prenylated, and phenylated phenazines [55,56,57,58,59,60,61,62,63]. PCA (**1**) is secreted by several pseudomonads and streptomycetes as a redox active agent that mediates nutrient cycling and iron ion uptake [64,65,66], as well as being a key intermediate to the biosynthesis of many other phenazines [54,67].

Bacilli that coexist in the same habitats as *Pseudomonas* strains [21,31,34,36] would benefit from adaptation to phenazines and other antibiotics from pseudomonads. Generally, microorganisms survive and adapt to antibiotics by inactivation [68,69,70], excretion [71,72], release of defensive and/or protective compounds as counterstrike [25], or even by modifying the target proteins [73,74,75]. Bacilli react in several ways to chemicals released by pseudomonads: *B. velezensis* counteracted against pyochelin secreted by pseudomonads by producing the antibiotics bacillaene and difficidin-like polyketides against the producer [23,25]. *B. amyloliquefaciens* reduced its cell membrane permeability in response to antibiotics from *Pseudomonas chlororaphis* [22]. *B. subtilis* with a defective extracellular matrix sporulated upon contact with *P. chlororaphis* [76]. *B. subtilis* and *B. licheniformis* detoxified 2-heptyl-4-hydroxyquinoline *N*-oxide from *P. aeruginosa* by glucosylation [68]. Similarly, *Bacillus* sp. G2112, and likely the other *Bacilli* tested (Table S1), survived phenazine-1-carboxylic acid (**1**) released by *Pseudomonas* sp. G124 by glycosylating and imino group formation yielding red imino-5*N*-(1′β-D-glucosyl)-dihydrophenazine-1-carboxylic acids **2** and **3**, thereby rendering PCA (**1**) inactive.

Glycosylation as a resistance and detoxification mechanism is widespread in bacteria, fungi, algae, plants, and man [68,69,70,71,72,77]. Although detoxification by glycosylation of a phenazine compound, namely 1-phenazinol, by *Caenorhabditis elegans* was described [78], the combination of both glycosylation and imino group formation as a resistance mechanism of a *Bacillus* strain has not been reported. Interestingly, *Bacillus* sp. G2112 modifies PCA (**1**) with an imino group either at position 3 or 7. The mechanisms for the simultaneous production of both 3- and 7-imino derivatives need to be elucidated in order to determine if *Bacillus* sp. G2112 recruited already existing enzymes to inactivate phenazine-1-carboxylic acid (**1**) as an emergency program or if it has a gene cluster tailored to the modification of phenazine-1-carboxylic acid (**1**) and similar compounds.

The red compounds **2** and **3** represent new glucosylated phenazine derivatives different to known glycosylated phenazines [47,56,61,62,63,79,80,81,82,83]. So far, no *N*-glycosylated phenazines have been reported. The glucosylation at *N*5 of the imino-dihydrophenazine-1-carboxylic acid core structure leading to atropisomers is unprecedented (see Figure S52 for comparison). Moreover, apart from the *O*-glucosylated phenazine derivatives produced by *C. elegans* [78] during detoxification of 1-phenazinol from *P. aeruginosa*, all other known glycosylated phenazines are biosynthesized by a single producer organism, usually *Streptomyces* spp. or *Kitasatospora* spp. (Figure S52) [47,56,61,62,63,79,80,81,82,83].

Although PCA (**1**) constitutes an important secondary metabolite produced by many pseudomonads and functions as an important antimicrobial compound against plant pathogens [54,84], thereby contributing to shaping microbial communities [85,86], very few detoxification mechanisms are known for it [87,88,89,90,91]. These detoxifications, identified from *Mycobacteria*, *Sphingomonas*, and *Aspergillus* spp., converted PCA (**1**) to various decarboxylated phenazine alcohols [88,89,90,91,92] and ring-opened carbonitriles and oximes [87]. *Bacillus* sp. G2112 performed, instead, a two-site modification introducing an imino group and *N*-glucosylation, thus rendering PCA (**1**) very polar and inactive, and by doing so enabled itself to coexist well with *Pseudomonas* sp. G124. However, this process also undermines *Pseudomonas* sp. G124′s major principle against competing microorganisms, including plant pathogens [54,84].

The observed detoxification of PCA (**1**) by *Bacillus* sp. G2112 demonstrates how interactions with other microorganisms may seriously affect the success of biocontrol under natural/field conditions. Knowing that resistance mechanisms are widespread among microorganisms [68,69,72,78], it would be important to consider their impact on biocontrol organisms during product development. Unraveling the enzymatic mechanisms that enabled the detoxification of PCA (**1**) by *Bacillus* sp. G2112, a current undertaking of our lab, would reveal how widespread this mechanism may be among microorganisms that coexist with pseudomonads in soil and plant ecosystems.

## 4. Materials and Methods

Chemicals: HPLC-grade methanol and acetonitrile were purchased from VWR (Darmstadt, Germany), soy flour (Hensel Vollsoja) was from W. Schoenenberger GmbH (Magstadt, Germany), and other medium ingredients were obtained from Carl Roth (Karlsruhe, Germany). D-glucose, D-maltose, and D-lactose were purchased from Merck (Darmstadt, Germany), *N*-Methyl-*N*-(trimethylsilyl)trifluoracetamide, L-glucose, and L-cysteine methyl ester from BLD Pharmatech GmbH (Reinbek, Germany), 4-fluorobenzyl isothiocyanate from TCI (Tokyo, Japan), and deuterated solvents (CD_3_OD, D_2_O, and CDCl_3_) from Deutero GmbH (Kastellaun, Germany).

Instruments: LC–MS was performed using a Phenomenex Synergi polar RP column (250 × 2.5 mm, 4 μm) using a Thermo Fisher Surveyor HPLC (Thermo Fisher, Dreieich; Germany) or Waters Acquity UPLC system (Waters GmbH, Eschborn, Germany) equipped with a PDA detector connected to an LTQ mass spectrometer (Thermo Fisher, San Jose, CA, USA) fitted with a heated electrospray ionization source (HESI II) operated in positive ionization mode. Analytical separation conditions were solvent A (water 0.1% acetic acid) and solvent B (acetonitrile 0.1% acetic acid) using programmed elution at a flow rate of 220 μL/min. HPLC programme: isocratic elution 2% B 1 min, 2% to 100% B in 25 min, and isocratic elution at 100% B for 3 min. UV spectra were recorded from 200–800 nm and mass spectra were recorded at a mass range of *m*/*z* 50–2000 Da.

High-resolution mass spectra were recorded using an LTQ Orbitrap XL mass spectrometer (Thermo Fisher, Bremen, Germany) with a heated electrospray ionization source (HESI II) operated in positive ionization mode connected to a Dionex Ultimate 3000 UHPLC system. The HPLC conditions were identical to above. High-resolution mass spectra were obtained after fresh calibration of the instrument at resolution setting 100,000 and using the lock mass function.

For GC–MS, a TraceGC Ultra connected to an ISQ mass spectrometer (Thermo Fisher, Dreieich, Germany) fitted with an Optima 5MS GLC column (30 m × 0.25 mm, 0.25 μm, Macherey Nagel, Düren, Germany) was used. The GC was operated in splitless mode with helium as the carrier gas at flow rate of 1 mL/min. The GC programme was as follows: initial temperature 50 °C for 0 min, first ramp at 20 °C/min to 80 °C, second ramp at 7 °C/min to 280 °C, isotherm for 5 min. The mass spectrometer was operated with electron impact ionization at 80 eV in positive ionization mode at a mass range of 41–850 Da. The MS transfer line temperature was set to 290 °C, and the ion source temperature was set to 220 °C.

NMR data were acquired using a Bruker Avance III 600 spectrometer (^1^H 600 MHz, ^13^C 151 MHz) equipped with a TCI-H/C/N triple resonance cryoprobe with a Z-gradient (Bruker, Rheinstetten, Germany). The NMR spectrometer was calibrated to residual CDCl_3_ (^13^C 77.16, ^1^H 7.26 ppm), D_2_O (^1^H 4.79), or CD_3_OD signals (^13^C 49.00, ^1^H 3.31 ppm), respectively [93].

Microorganisms: *Pseudomonas fluorescens* DSM6506, *Pseudomonas syringae* pv. *glycinea* 1a/96, *Bacillus subtilis* DSM10, *Bacillus pumilus* DSM27, *Bacillus thuringiensis* DSM 2046, *Bacillus amyloliquefaciens* DSM7, and *Erwinia tracheiphila* DSM21139 were obtained from the German Collection of Microorganisms and Cell Cultures GmbH (Braunscheig, Germany). *Pseudomonas syringae* pv. *syringae* 22d/93 was from Dr. Beate Völksch, Friedrich Schiller University Jena. *Fusarium equiseti* FSU5459 was obtained from the collection of the Friedrich Schiller University Jena. *Bacillus* sp. G2112 and *Pseudomonas* sp. G124 were isolated from cucumber (*Cucumis sativus*) leaves taken from farms from the island of Reichenau near Konstanz, Germany.

Identification of microorganisms: From genomic DNA of the isolates G2112 and G124, the 16S rDNA was amplified using primers 8f (5′-AGAGTTTGATCCTGCCTCAG-3′) and 1492r (5′-GGTTACCTTGTTACGACTT-3′) [94] and sequenced at Eurofins Genomics Ebersberg, Germany. Sequences were assembled using DNA Baser (Heracle BioSoft SRL, Arges, Romania). The 16*S* rDNA sequences were compared to the GenBank database using BLASTN [95,96]. The phylogenetic relationship to selected closely and distantly related microorganisms was established using MEGA 11 [97] with the following parameters: sequences were aligned using Muscle [98] with default settings. Phylogenetic trees were constructed using the neighbor-joining [99] method with default settings including the bootstrap [100] test (1000 replications). The *Pseudomonas* sp. G124 and *Bacillus* sp. G2112 16*S* rDNA sequences were deposited at Genbank with the accession numbers OR725974 and OR725975, respectively.

Cultivation conditions: Microorganisms were cultivated on soy flour mannitol (20 g soy flour, 20 g mannitol, per L H_2_O) [40], King’s B (20 g peptone, 10 g glycerol, 1.5 g K_2_HPO_4_, 1.5 g MgSO_4_ 7H_2_O, per L H_2_O) [41], or modified 5b (8.6 g glycerol, 2.6 g KH_2_PO_4_, 5.5 g Na_2_HPO_4_, 2.5 g NH_4_Cl, 1 g Na_2_SO_4_, 0.1 g MgCl_2_ 6H_2_O, 0.01 g FeSO_4_ 7H_2_O; 0.01 g MnSO_4_ H_2_O, per L H_2_O) [42,43] media (containing 20 g agar when required) at 28 °C for the indicated time. Test organisms were usually grown overnight prior to use unless otherwise stated.

Red pigment production: Overnight King’s B liquid pre-cultures of *Bacillus* sp. G2112 and *Pseudomonas* sp. G124 were adjusted with sterile water to 1 × 10^6^ and 1 × 10^8^ cfu/mL and spotted onto King’s B agar plates as mono- and co-cultures. For co-cultivation, *Pseudomonas* sp. G124 and *Bacillus* sp. G2112 were either grown side-by-side on the same agar allowing reaction to metabolites secreted both into the agar medium and the headspace, or the two organisms were grown in three-compartment agar plates so that exchange could only occur via the headspace. Subsequently, the co-cultivation of *Bacillus* sp. G2112 and *Pseudomonas* sp. G124 neighboring each other on the same agar was modified by spotting the microorganisms either 2 or 4 cm apart from each other to investigate the role of distance on pigment production. All agar plates were incubated for 8 d and the experiments were performed in triplicates and photographed for documentation.

To rule out that pigments were produced directly from metal ions or nitrogen sources in the media, the assay above was repeated on 5b glycerol agar plates lacking either FeSO_4_ 7H_2_O or MnSO_4_ H_2_O or both, as well as on 5b glycerol agar plates in which NH_4_Cl was completely replaced by selected L-amino acids (glycine, alanine, asparagine, glutamine, lysine, phenylalanine, tryptophan, tyrosine, and proline; 2.5 g/L).

Moreover, pigment production was also investigated using 100 mL liquid King’s B cultures in 250 mL Erlenmeyer flasks. Monocultures were inoculated with 50 µL overnight pre-cultures of *Bacillus* sp. G2112 or *Pseudomonas* sp. G124. One set of co-cultures was inoculated at day 0 with 25 µL each of both *Bacillus* sp. G2112 and 25 µL *Pseudomonas* sp. G124. For the second set, 50 mL of media in 250 mL Erlenmeyer flasks was inoculated separately with 25 µL of *Bacillus* sp. G2112 or *Pseudomonas* sp. G124 and both cultures were combined after 24 h. The Erlenmeyer flasks were incubated at 28 °C with shaking at 120 rpm and observed for 12 d.

Identification of the roles of the interacting partners in pigment formation: In order to identify which organism was responsible for the red pigment formation, two assays were conducted. In the first assay, two inocula of *Pseudomonas* sp. G124 were placed well apart on the same King’s B agar and an inoculum of *Bacillus* sp. G2112 was placed 2 cm away from one of the *Pseudomonas* sp. G124 colonies. The plates were observed for 7 d. In a second assay, spent King’s B agar of *Pseudomonas* sp. G124 (3 plates, Ø 8.6 cm, 25 mL medium each) was harvested after 1, 3, and 6 d and extracted with 50 mL methanol. The extracts were concentrated with a rotary evaporator (Büchi, Flawil, Switzerland), re-dissolved in 2 mL methanol, then filter-sterilized and stored at −20 °C until use. A total of 50 µL of extract was assayed against 1 d-old King’s B agar cultures of *Bacillus* sp. G2112 by adding the extract into holes made with flame-sterilized wide ends of glass Pasteur pipettes. The plates were incubated at 28 °C and observed for 2 d for pigment production. Results were documented by taking photos.

Screening of bacilli and pseudomonads for red pigment production: In order to determine whether pigment production was specific to only *Bacillus* sp. G2112 and *Pseudomonas* sp. G124 in a co-culture, each strain was co-cultured with other *Pseudomonas* strains and *Bacillus* strains respectively (Table S1), on King’s B agar for 7 d and analyzed for pigment production.

Purification and identification of the red pigment-inducing compound: *Pseudomonas* sp. G124 was grown for 7 d on King’s B agar plates (Ø 8.6 cm, 25 mL medium, 3 plates). From each plate the spent King’s B agar was cut into small pieces with a scalpel and extracted in a 50 mL Erlenmeyer flask with 30 mL of ddH_2_O for 30 min. The three extracts were divided into three equal parts that were adjusted to pH 3, 7, and 9.5, respectively. The pH-adjusted aqueous samples (ca. 10 mL) were extracted with an equal volume of ethyl acetate. The organic layer was dried over Na_2_SO_4_ and both aqueous and organic extracts were dried using a rotary evaporator. The dry extracts were re-suspended in 1 mL methanol and sterile-filtered (0.22 µm). A total of 50 µL of each extract was tested in agar diffusion assays against *Bacillus* sp. G2112 spread on King’s B agar from an overnight pre-culture as reported above. Methanol (50 µL) served as a control. The plates were incubated at 28 °C and analyzed for pigment production after 2 d. Photos were taken for documentation.

For large scale purification of PCA (**1**), 2 kg of spent 5b glycerol agar from agar plates inoculated with *Pseudomonas* sp. G124 and cultivated for 11 d at 28 °C was extracted for 1 h with 2.5 L ddH_2_O at pH 9.5. The extract was filtered through cotton wool and remnant cells and insoluble material was removed by centrifugation (4612 *g*, 20 min, 18 °C). The aqueous extract (2.5 L) was adjusted to pH 3.5 with 6 N HCl and extracted with an equal volume of ethyl acetate using a separatory funnel. The ethyl acetate layer was collected, dried over Na_2_SO_4_ and evaporated *in vacuo*. The residue was redissolved in 100 mL ddH_2_O adjusted to pH 9.5 with 8 N NaOH. The solution was transferred into a separatory funnel and an equal volume of chloroform was added to it. About 1 mL of 6 N HCl was added dropwise into the aqueous layer until pH 3–4, leading to a yellow precipitate. The mixture was shaken vigorously for about 1 min to dissolve the precipitate in the organic phase. After complete phase separation the organic layer was collected. The aqueous layer was extracted once again until the organic phase became colorless. All organic extracts were dried over Na_2_SO_4_ and concentrated *in vacuo*. The obtained yellow residue was washed once with 5 mL methanol and dried. Portions of the obtained compound were dissolved in methanol for LC–MS analysis and in CDCl_3_ for NMR analysis, respectively.

Antibiotic effect of phenazine-1-carboxylic acid (PCA, **1**) and the derivative **3** on *Bacillus* sp. G2112: To determine the antibiotic effect of PCA (**1**) on *Bacillus* sp. G2112, warm 5b agar (50–60 °C) was supplemented with PCA (**1**) to final concentrations of 0, 0.18, 1.8, 18, and 180 µM and poured into Petri dishes (Ø 5.2 cm, 10 mL medium). A total of 2 µL of a *Bacillus* sp. G2112 overnight culture was spotted twice onto PCA-(**1**)-containing agar plates to produce two colonies per plate. The experiment was repeated once and all the plates were incubated at 28 °C for 4 d.

The toxicities of PCA (**1**) and 3-imino-5*N*-(1′-β-D-glucosyl)-3,5-dihydrophenazine-1-carboxylic acid (**3**) against *Bacillus* sp. G2112 was compared using paper disc agar diffusion assays of 20 µL of aqueous 20 mM PCA (**1**) (pH 9) and 20 µL of methanolic 20 mM 3-imino-5*N*-(1′-β-D-glucosyl)-3,5-dihydrophenazine-1-carboxylic acid (**3**). The plates were inoculated with an overnight culture *Bacillus* sp. G2112 spread over the agar plate. The test compounds were added onto a sterile paper disc placed in the middle of the plates. The plates were incubated at 28 °C for 1 d. Photos were taken for documentation.

Red pigment formation provoked by phenazine-1-carboxylic acid (PCA, **1**): Pigment induction at sub-lethal concentrations of PCA (**1**) was investigated. A total of 50 µL of dilute overnight culture of *Bacillus* sp. G2112 (adjusted to 10^6^ cfu/mL) was spread onto eight 5b glycerol agar plates (Ø 8.5 cm, 25 mL medium) and incubated for 4 h at 28 °C. One hole was punched into each quarter of the agar plates. The plates were divided into four groups of 2 plates each. Group 1 received 25 µL of 1 mg/mL aqueous PCA (**1**) per hole; group 2 received 50 µL of 1 mg/mL per hole; group 3 received 50 µL of 1 mg/mL per hole twice within 16 h; and group 4 received 50 µL of 3 mg/mL PCA per hole. The plates were incubated at 28 °C for 4 d. Photos were taken for documentation.

Production of red pigments in 5b liquid culture of *Bacillus* sp. G2112 using PCA (**1**): A 1 mL overnight pre-culture of *Bacillus* sp. G2112 was used to inoculate 0.7 L of 5b liquid medium that contained 1.8 g/L NH_4_Cl and 0.7 g/L glycine (5b + Gly) in a 2 L Erlenmeyer flask. This starter culture was grown to an OD_600_ of 0.3–0.4 at 28 °C and 120 rpm. A total of 100 mL each of the starter culture was transferred into six 250 mL Erlenmeyer flasks. One group of three flasks received 0.3 mL of 10 mg/mL aqueous PCA (**1**) (pH 9, final concentration 134 µM) and the other group received an equivalent volume of sterile alkaline double-distilled water. The culture flasks were incubated at 28 °C, 120 rpm for 6 d. A total of 5 mL spent media were harvested from each flask after 1 d, 2 d, 3 d, and 6 d, centrifuged (4612 *g*, 20 min, 18 °C), and the supernatants were analyzed by LC–MS.

Extraction and purification of red pigments from *Bacillus* sp. G2112: A total of 150 mL 5b + Gly broth was inoculated with 2 mL of *Bacillus* sp. G2112 overnight culture. This pre-culture was grown at 28 °C, 120 rpm for 2 d. Seven 2 L baffled Erlenmeyer flasks containing 730 mL of 5b + Gly medium were inoculated with 20 mL of the pre-culture. At an OD_600_ of 0.3, 2.25 mL of 8 mg/mL alkaline aqueous PCA (pH 9) was added into each flask. Another 3 mL and 2.25 mL of the PCA solution were added into each flask after 2 d and 3 d, respectively. The culture flasks were incubated for 12 d. The spent medium was centrifuged (4612 *g*, 20 min, 18 °C) and the supernatant was concentrated to ca. 1 L by lyophilization. The pH was adjusted to 6.5 and the sample was applied onto a Diaion HP20 column (120 g, 4.5 × 35 cm, equilibrated with ddH_2_O before use, Supelco, Taufkirchen, Germany). The first 200 mL column flow-through was discarded and the following ca. 600 mL flow-through was collected as FT. The bound compounds were eluted with 300 mL of 15%, 30%, and 60% aqueous acetonitrile, and 100% acetonitrile (F1, F2, F3, and F4, respectively). The HP20 column was re-equilibrated with water and FT was reinjected and eluted with 30% aqueous acetonitrile (FTF1).

All fractions were lyophilized and analyzed by LC–DAD–MS. Then, fractions FTF1 and F1 were subjected to medium pressure liquid chromatography (MPLC, Sepacore system, Büchi, Essen, Germany) separately. A Polygoprep C8 column (28 cm × 1.8 cm i.d., 60–50 μm particle size, Macherey-Nagel, Düren, Germany) was used for the further purification. The MPLC column was packed as slurry in methanol and equilibrated with 1% aqueous acetonitrile prior to separation. MPLC conditions: solvent A was ddH_2_O and solvent B acetonitrile. Programed elution at 15 mL/min was used starting with 1% B for 1 min, gradient elution 1–30% B in 15 min, gradient elution 3–100% B in 15 min, and isocratic elution 100% B for 3 min. One-minute fractions were collected in test tubes and lyophilized. To further purify the red pigments, MPLC fractions 8–10 from both FTF1 and F1 separations were combined and concentrated. A total of 3.2 mg of sample were re-dissolved in 400 µL ddH_2_O for semi-preparative HPLC separation by an Agilent 1100 HPLC system (Waldbronn, Germany) fitted with a Phenomenex Synergi polar RP column (250 × 4.6 mm, 4 μm, Phenomenex, Aschaffenburg, Germany) coupled to a Gilson 206 fraction collector (Gilson International, Berlin, Germany). HPLC conditions: 20 μL were injected per run. ddH_2_O 0.1% acetic acid served as solvent A and acetonitrile 0.1% acetic acid served as solvent B at 0.8 mL/min flow rate. HPLC elution started at 2% B for 1 min, followed by gradient elution from 2–8% B in 10 min, 8–20% B in 8 min, 20–70% B in 7 min, 70% B for 1 min, and re-equilibration at 2% B for 8 min. Fractions (0.5 min) were collected from 4 to 27 min. Fractions with red color were analyzed by LC–MS for their purity and pure contiguous fractions with the same retention time in LC–MS were combined and lyophilized.

Structure elucidation of red pigments: Pure samples from the HPLC fractionation were identified by LC–DAD–MS and subjected to HR-HESI-MS/MS. The samples (ca. 0.3–0.5 mg) were dissolved in CD_3_OD for NMR analysis. Their ^1^H- ^13^C- ^1^H-^1^H-COSY, ^1^H-^13^C-HMBC, ^1^H-^13^C HSQC, and NOESY spectra were recorded. The analytical data can be found in the SI.

Characterization of the sugar moiety of the red pigments **2** and **3**: The purified pigments **2** and **3** (50–100 μg), and maltose and glucose standards were methanolysed and subsequently acetylated [101]: samples were dissolved in 200 μL methanol in 1.5 mL autosampler glass vials fitted with a septum lid. A total of 20 µL acetyl chloride was added dropwise and the vials were incubated at 80 °C for 1 h. The solvent was evaporated by rotary evaporation. FeCl_3_ crystals (~10 µg) were added into each vial followed by 20 μL of anhydrous acetic anhydride. The vials were incubated at room temperature for 20 min and intermittently shaken by hand. The samples were partitioned twice in 600 μL 50:50 ice cold water–ethyl acetate with vigorous shaking, then left standing for phase separation. The organic phase was dried over Na_2_SO_4_, filtered through cotton, and concentrated in a gentle N_2_ stream to about 50 µL. A total of 1 µL of the sample was injected into the GC–MS.

The absolute configuration of the glucose moiety of **2** was determined by a modified method of Tanaka et al. [50]. A total of 0.3 mg of 7-imino-5*N*-(1′-β-D-glucosyl)-5,7dihydrophenazine-1-carboxylic acid (**2**) and standard sugars (D-maltose, D-glucose and L-glucose) in glass screw-capped vials were dissolved in 20 µL of water. A total of 20 µL of 1 N H_2_SO_4_ was added and the solutions were incubated at 80 °C for 3 h. The solutions were neutralized using 2 M NaOH and evaporated to dryness. A total of 20 µL of 1% L-cysteine methyl ester in pyridine was added and the solutions incubated at 60 °C, 600 rpm for 1 h. Then, 5 µL of 4-fluorobenzyl isothiocyanate was added into the vials that were further incubated for another 1 h. The derivatized samples were directly analyzed by HPLC–MS using Nucleodur C8 Gravity column (250 mm × 2 mm, 5 μm, Macherey-Nagel, Düren, Germany) using the following program: solvents (H_2_O A, acetonitrile B) + 0.1% acetic acid, flow rate 220 µL/min, 2% B 1 min, 2–50% B 10 min, 50–55% B 3 min, 55–100% B 7 min, 100% B 3 min, and re-equilibration 7 min.

## Data Availability

The data presented in this study are available in the Supporting Information and from the authors.

## Supplementary Materials

The Supporting Information can be downloaded at: https://www.mdpi.com/article/10.3390/molecules29030589/s1. Figure S1: Neighbour-joining tree of *Bacillus* sp. G2112 and other bacilli. Figure S2: Neighbour-joining tree of *Pseudomonas* sp. G124 and other *Pseudomonas* spp. Figure S3: Bioassays of *Bacillus* sp. G2112 against (A) *Fusarium equiseti* and (B) *Erwinia tracheiphila*. Figure S4: Bioassays of *Pseudomonas* sp. G124 against (A) *Fusarium equiseti* and (B) *Erwinia tracheiphila*. Figure S5: Conditions for red pigment formation. Figure S6: Effect of the distance between *Pseudomonas* sp. G124 and *Bacillus* sp. G2112 colonies on the pigment production on 5b agar. Figure S7: Comparison of the metabolite profiles (base peak ion traces and UV traces) of (A) *Pseudomonas* sp. G124 which caused red pigmentation around *Bacillus* sp. G2112 colonies and (B) *Pseudomonas syingae* pv*. syringae* 22d/93 which did not cause red pigmentation around *Bacillus* sp. G2112. Figure S8: UV-Vis spectrum of phenazine-1-carboxylic acid (1). Figure S9: HR-ESI-MS of phenazine-1-carboxylic acid (1). Figure S10: ^1^H-NMR of phenazine-1-carboxylic acid (1). Figure S11: ^13^C-NMR of phenazine-1-carboxylic acid (1) at 151 MHz in CDCl_3_. Figure S12: ^1^H-^1^H-COSY NMR of phenazine-1-carboxylic acid (1) at 600 MHz in CDCl_3_. Figure S13: ^1^H-^13^C-HSQC NMR of phenazine-1-carboxylic acid (1) at 600 MHz in CDCl_3_. Figure S14: ^1^H-^13^C-HMBC NMR of phenazine-1-carboxylic acid (1) at 600 MHz in CDCl_3_. Figure S15: Effect of different PCA (1) concentrations on the growth of *Bacillus* sp. G2112 and its production of red pigments. Figure S16: Conversion of PCA (1) to red pigments 2 and 3 by *Bacillus* sp. G2112. Figure S17: UV-Vis spectrum of 7-imino-5*N*-(1’β-D-glucopyranosyl)-5,7-dihydrophenazine-1-carboxylic acid (2). Figure S18: HR-ESI-MS spectrum of 7-imino-5*N*-(1’β-D-glucopyranosyl)-5,7-dihydrophenazine-1-carboxylic acid (2). Figure S19: HR-ESI-MS/MS spectrum of 7-imino-5*N*-(1’β-D-glucopyranosyl)-5,7-dihydrophenazine-1-carboxylic acid (2). Figure S20: ^1^H-NMR spectrum of 7-imino-5*N*-(1’β-D-glucopyranosyl)-5,7-dihydrophenazine-1-carboxylic acid (2). Figure S21: ^13^C-NMR spectrum of 7-imino-5*N*-(1’β-D-glucopyranosyl)-5,7-dihydro-phenazine-1-carboxylic acid (2). Figure S22: ^1^H-^1^H-COSY NMR spectrum of 7-imino-5*N*-(1’β-D-glucopyranosyl)-5,7-dihydrophenazine-1-carboxylic acid (2). Figure S23: ^1^H-^1^H-COSY NMR spectrum of the aromatic region of 7-imino-5*N*-(1’β-D-glucopyranosyl)-5,7-dihydrophenazine-1-carboxylic acid (2). Figure S24: ^1^H-^13^C-HSQC NMR spectrum of the aromatic region of 7-imino-5*N*-(1’β-D-glucopyranosyl)-5,7-dihydrophenazine-1-carboxylic acid (2). Figure S25: ^1^H-^13^C HMBC NMR spectrum of the aromatic region of 7-imino-5*N*-(1’β-D-glucopyranosyl)-5,7-dihydrophenazine-1-carboxylic acid (2). Figure S26: Structure of 7-imino-5*N*-(1’β-D-glucopyranosyl)-5,7-dihydrophenazine-1-carboxylic acid (2). Figure S27: UV-Vis spectrum of 3-imino-5*N*-(1’β-D-glucopyranosyl)-3,5-dihydrophenazine-1-carboxylic acid (3). Figure S28: HR-ESI-MS of 3-imino-5*N*-(1’β-D-glucopyranosyl)-3,5-dihydrophenazine-1-carboxylic acid (3). Figure S29: HR-ESI-MS/MS of 3-imino-5*N*-(1’β-D-glucopyranosyl)-3,5-dihydrophenazine-1-carboxylic acid (3). Figure S30: ^1^H-NMR spectrum of 3-imino-5*N*-(1’β-D-glucopyranosyl)-3,5-dihydrophenazine-1-carboxylic acid (3). Figure S31: ^1^H-^1^H-COSY NMR spectrum of 3-imino-5*N*-(1’β-D-glucopyranosyl)-3,5-dihydrophenazine-1-carboxylic acid (3). Figure S32: ^1^H-^1^H-COSY NMR spectrum of the aromatic region of 3-imino-5*N*-(1’β-D-glucopyranosyl)-3,5-dihydrophenazine-1-carboxylic acid (3). Figure S33: ^1^H-^13^C-HSQC NMR spectrum of 3-imino-5*N*-(1’β-D-glucopyranosyl)-3,5-dihydrophenazine-1-carboxylic acid (3). Figure S34: ^1^H-^13^C-HMBC NMR spectrum of 3-imino-5*N*-(1’β-D-glucopyranosyl)-3,5-dihydrophenazine-1-carboxylic acid (3). Figure S35: Structure of 3-imino-5*N*-(1’β-D-glucopyranosyl)-3,5-dihydrophenazine-1-carboxylic acid (3). Figure S36: HPLC identification of 7- amino-phenazine-1-carboxylic acid. Figure S37: UV-Vis 7-amino-phenazine-1-carboxylic acid (4). Figure S38: HR-ESI-MS of 7-amino-phenazine-1-carboxylic acid (4). Figure S39: HR-ESI-MS/MS of 7-amino-phenazine-1-carboxylic acid (4). Figure S40: ^1^H-NMR of 7-amino-phenazine-1-carboxylic acid (4) at 600 MHz in D_2_O. Figure S41: ^1^H-^1^H-COSY NMR of 7-amino-phenazine-1-carboxylic acid (4) at 600 MHz in D_2_O. Figure S42: ^1^H-^13^C-HSQC NMR of 7-amino-phenazine-1-carboxylic acid (4) at 600 MHz in D_2_O. Figure S43: ^1^H-^13^C HMBC NMR spectrum of 7-amino-phenazine-1-carboxylic acid (4) at 600 MHz in D_2_O. Figure S44: Structure of 7-aminophenazine-1-carboxylic acid (4). Figure S45: GC-MS identification of sugar moieties of 7-imino-5*N*-(1’β-D-glucopyranosyl)-5,7-dihydrophenazine-1-carboxylic acid (2) and 3-imino-5*N*-(1’β-D-glucopyranosyl)-3,5-dihydrophenazine-1-carboxylic acid (3). Figure S46: Determination of the stereochemistry of the glucose moiety of 7-imino-5*N*-(1’β-D-glucopyranosyl)-5,7-dihydrophenazine-1-carboxylic acid (2). Figure S47: NOESY NMR spectrum of 7-imino-5*N*-1’β-D-glucopyranosyl)-5,7-dihydrophenazine-1-carboxylic acid (2) and structures of its atropisomers 2a and 2b. Figure S48: Suggested 3-dimensional structures of the atropisomers of 7-imino-5*N*-1’β-D-glucopyranosyl)-5,7-dihydrophenazine-1-carboxylic acid (2a and 2b) fitting to the NOESY NMR correlations. Figure S49: NOESY NMR spectrum of 3-imino-5*N*-(1’β-D-glucopyranosyl)-3,5-dihydrophenazine-1-carboxylic acid (3) and structures of its atropisomers 3a and 3b. Figure S50: Suggested 3-dimensional structures of the atropisomers of 3-imino-5*N*-(1’β-D-glucopyranosyl)-3,5-dihydrophenazine-1-carboxylic acid (3a and 3b) fitting to the NOESY-NMR correlations. Figure S51: Agar diffusion bioassays with PCA (1) and 3-imino-5*N*-(1’β-D-glucopyranosyl)-3,5-dihydrophenazine-1-carboxylic acid (3). Figure S52: Structures of known glycosylated phenazines. Table S1: Red pigment formation in *Bacillus* and *Pseudomonas* strains in co-culture. Table S2: ^1^H-NMR (600 MHz, CD_3_OD) data for 7-imino-5*N*-(1’β-D-glucopyranosyl)-5,7-dihydrophenazine-1-carboxylic acid (2a and 2b). Table S3: ^1^H-NMR (600 MHz, CD_3_OD) data for 3-imino-5*N*-(1’β-D-glucopyranosyl)-3,5-dihydrophenazine-1-carboxylic acid (3a and 3b). Table S4: ^1^H-NMR (600 MHz, D_2_O) data for 7-aminophenazine-1-carboxylic acid (4).
