# Supplementary material for: Bacillus sp. G2112 Detoxifies Phenazine-1-carboxylic Acid by N5 Glucosylation
Source: Molecules. 2024 Jan 25;29(3):589. doi: 10.3390/molecules29030589 (PMC10856480; doi:10.3390/molecules29030589)
Supplement: Supplementary file 1 [file molecules-29-00589-s001.zip › molecules-2841599-supplementary.pdf]

Bacillus sp. G2112 detoxifies Phenazine-1-carboxylic acid by N5 Glucosylation

Kenechukwu Iloabuchi [1,2] and Dieter Spiteller [1,*]

Table of contents

Phylogenetic placement of *Bacillus* sp. G2112 4

Phylogenetic placement of *Pseudomonas* sp. G124 5

Activity of *Bacillus* sp. G2112 against plant pathogens 6

Activity of *Pseudomonas* sp. G124 against plant pathogens 6

Conditions for red pigment formation 7

Formation of red pigments by different *Pseudomonas* and *Bacillus* strains in co-culture 8

Phenazine-1-carboxylic acid (**1**) as precursor for red pigments 9

Differential metabolite profiling of the compound causing red pigmentation 9

UV-Vis spectrum of phenazine-1-carboxylic acid (**1**) 10

HR-ESI-MS of phenazine-1-carboxylic acid (**1**) 10

^{1}H-NMR of phenazine-1-carboxylic acid (**1**) at 600 MHz in $CDCl_3$ 11

^{13}C-NMR of phenazine-1-carboxylic acid (**1**) at 151 MHz in $CDCl_3$ 11

^{1}H-^{1}H-COSY NMR of phenazine-1-carboxylic acid (**1**) at 600 MHz in $CDCl_3$ 12

^{1}H-^{13}C-HSQC NMR of phenazine-1-carboxylic acid (**1**) at 600 MHz in $CDCl_3$ 13

^{1}H-^{13}C-HMBC NMR of phenazine-1-carboxylic acid (**1**) at 600 MHz in $CDCl_3$ 14

Bacillus sp. G2112 growth and pigment production in response to phenazine-1-carboxylic acid (PCA,1) 15

Conversion of phenazine-1-carboxylic acid (1) to imino-5*N*-(1′β-D-glucosyl)-dihydrophenazine-1-carboxylic acids 2 and 3 16

Structure elucidation of 7-imino-5*N*-(1′β-glucosyl)-5,7-dihydrophenazine-1-carboxylic acid (2) 17

UV-Vis spectrum of 7-imino-5*N*-(1′β-glucosyl)-5,7-dihydrophenazine-1-carboxylic acid (**2**) 17

HR-ESI-MS of 7-imino-5*N*-(1′β-D-glucopyranosyl)-5,7-dihydrophenazine-1-carboxylic acid (**2**) 17

HR-ESI-MS/MS of 7-imino-5*N*-(1′β-D-glucopyranosyl)-5,7-dihydrophenazine-1-carboxylic acid (**2**) 18

^{1}H-NMR of 7-imino-5*N*-(1′β-glucosyl)-5,7-dihydrophenazine-1-carboxylic acid (**2**) at 600 MHz in CD_3OD..18

^{13}C-NMR of 7-imino-5*N*-(1′β-D-glucopyranosyl)-5,7-dihydrophenazine-1-carboxylic acid (**2**) at 151 MHz in CD_3OD..19

^{1}H-^{1}H-Cosy NMR of 7-imino-5*N*-(1′β-glucosyl)-phenazine-1-carboxylic acid (**2**) at 600 MHz in CD_3OD..20

^{1}H-^{13}C-HSQC NMR of 7-imino-5*N*-(1′β-D-glucopyranosyl)-5,7-dihydrophenazine-1-carboxylic acid (**2**) at 600 MHz in CD_3OD 22

^{1}H-^{13}C-HMBC NMR of 7-imino-5*N*-(1′β-D-glucopyranosyl)-5,7-dihydrophenazine-1-carboxylic acid (**2**) at 600 MHz in CD_3OD 23

^{1}H-^{1}H-COSY and ^{1}H-^{13}C-HMBC correlations of 7-imino-5*N*-(1′β-D-glucopyranosyl)-5,7-dihydrophenazine-1-carboxylic acid (**2**) 24

NMR data of 7-imino-5*N*-(1′β-D-glucopyranosyl)-5,7-dihydrophenazine-1-carboxylic acid (**2**) 25

Structure elucidation of 3-imino-5*N*-(1′β-D-glucopyranosyl)-3,5-dihydrophenazine-1-carboxylic acid (3) 26

UV-Vis spectrum of 3-imino-5*N*-(1′β-D-glucopyranosyl)-3,5-dihydrophenazine-1-carboxylic acid (**3**) 26

HR-ESI-MS of 3-imino-5*N*-(1′β-D-glucopyranosyl)-3,5-dihydrophenazine-1-carboxylic acid (**3**) 26

HR-ESI-MS/MS of 3-imino-5*N*-(1′β-D-glucopyranosyl)-3,5-dihydrophenazine-1-carboxylic acid (**3**)...27

H-NMR of 3-imino-5*N*-(1′β-D-glucopyranosyl)-3,5-dihydrophenazine-1-carboxylic acid (**3**) at 600 MHz in CD_3OD..27

^{1}H-^{1}H-COSY NMR of 3-imino-5*N*-(1′β-D-glucopyranosyl)-3,5-dihydrophenazine-1-carboxylic acid (**3**) at 600 MHz in CD_3OD 28

^{1}H-^{13}C-HSQC NMR of 3-imino-5*N*-(1′β-D-glucopyranosyl)-3,5-dihydrophenazine-1-carboxylic acid (**3**) at 600 MHz in CD_3OD 30

^{1}H-^{13}C-HMBC NMR of 3-imino-5*N*-(1′β-D-glucopyranosyl)-3,5-dihydrophenazine-1-carboxylic acid (**3**) at 600 MHz in CD_3OD 31

^{1}H-^{1}H-COSY and ^{1}H-^{13}C-HMBC correlations of 3-imino-5*N*-(1′β-D-glucopyranosyl)-3,5-dihydrophenazine-1-carboxylic acid (**3**) 32

NMR data of 3-imino-5*N*-(1′β-D-glucopyranosyl)-3,5-dihydrophenazine-1-carboxylic acid (**3**) 33

Structure elucidation of 7-amino-phenazine-1-carboxylic acid (4) 34

7-Amino-phenazine-1-carboxylic acid (**4**) from purification of spent *Bacillus* sp. G2112 medium and after hydrolysis from 7-imino-5*N*-(1′β-D-glucosyl)-5,7-dihydrophenazine-1-carboxylic acid (**2**) 34

UV-Vis spectrum of 7-amino-phenazine-1-carboxylic acid (**4**)....35
HR-ESI-MS of 7-amino-phenazine-1-carboxylic acid (**4**)....35
HR-ESI-MS/MS of 7-amino-phenazine-1-carboxylic acid (**4**)....36
^{1}H-NMR of 7-amino-phenazine-1-carboxylic acid (**4**) at 600 MHz in D_2O....36
^{1}H-^{1}H-COSY NMR of 7-amino-phenazine-1-carboxylic acid (**4**) at 600 MHz in D_2O....37
^{1}H-^{13}C-HSQC NMR 7-amino-phenazine-1-carboxylic acid (**4**) at 600 MHz in D_2O....38
^{1}H-^{13}C-HMBC NMR of 7-amino-phenazine-1-carboxylic acid (**4**) at 600 MHz in D_2O....39
^{1}H-^{1}H COSY and ^{1}H-^{13}C HMBC correlations of 7-aminophenazine-1-carboxylic acid (**4**)...40
NMR data of 7-aminophenazine-1-carboxylic acid (**4**)....40
Identification of the sugar moiety of imino-5*N*-(1′β-D-glucopyranosyl)-dihyrophenazine-1-carboxylic acids **2** and **3**....41
Determination of the stereochemistry of the sugar moiety of imino-5*N*-(1′β-D-glucopyranosyl)-dihyrophenazine-1-carboxylic acids **2** and **3**....42
Atropisomers of the imino-5*N*-(1′β-D-glucopyranosyl)-dihydrophenazine-1-carboxylic acids 2 and 3....43
NOESY NMR spectrum of 7-imino-5*N*-(1′β-glucopyranosyl)-5,7-dihydrophenazine-1-carboxylic acid (**2**)....43
NOESY NMR spectrum of 3-imino-5*N*-(1′β-D-glucopyranosyl)-3,5-dihydrophenazine-1-carboxylic acid (**3**)....45
Toxicity assays of phenazine-1-carboxylic acid (**1**) and 3-imino-5*N*-(1′β-D-glucopyranosyl)-3,5-dihydrophenazine-1-carboxylic acid (**3**)....46
Structures of all known glycosylated phenazines....47
References....48

Phylogenetic placement of *Bacillus* sp. G2112

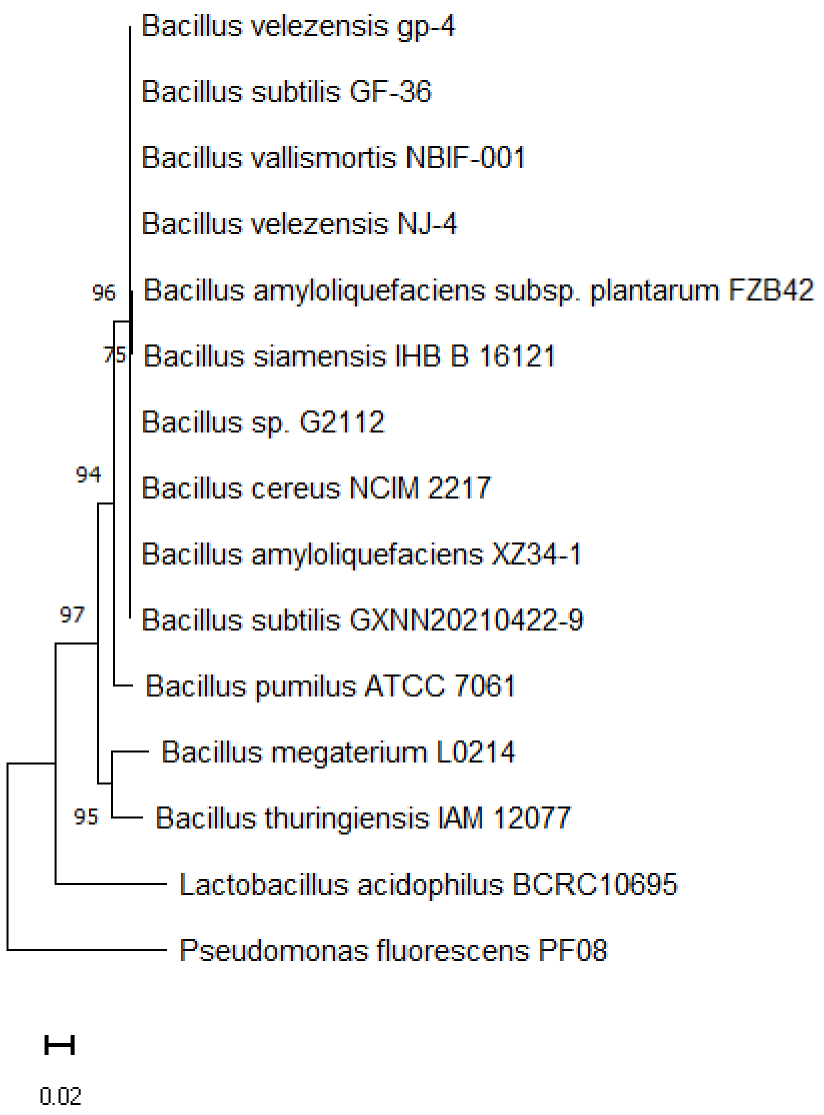

Figure S1: Neighbour-joining tree [1] of *Bacillus* sp. G2112 and other bacilli. The sequence of *Pseudomonas fluorescens* PF08 obtained from GenBank served as outgroup. Sequences used to construct the tree were aligned using the muscle algorithm [2]. Bootstrap (1000 replications) values [3] are given at the branching points. Phylogenetic analysis was performed using Mega 11 [4]. The tree was drawn to scale, with branch lengths in the same units as those of the evolutionary distances used to infer the phylogenetic tree. The evolutionary distances were computed using the Maximum Composite Likelihood method and are in the units of the number of base substitutions per site (scale bar).

Phylogenetic placement of *Pseudomonas* sp. G124

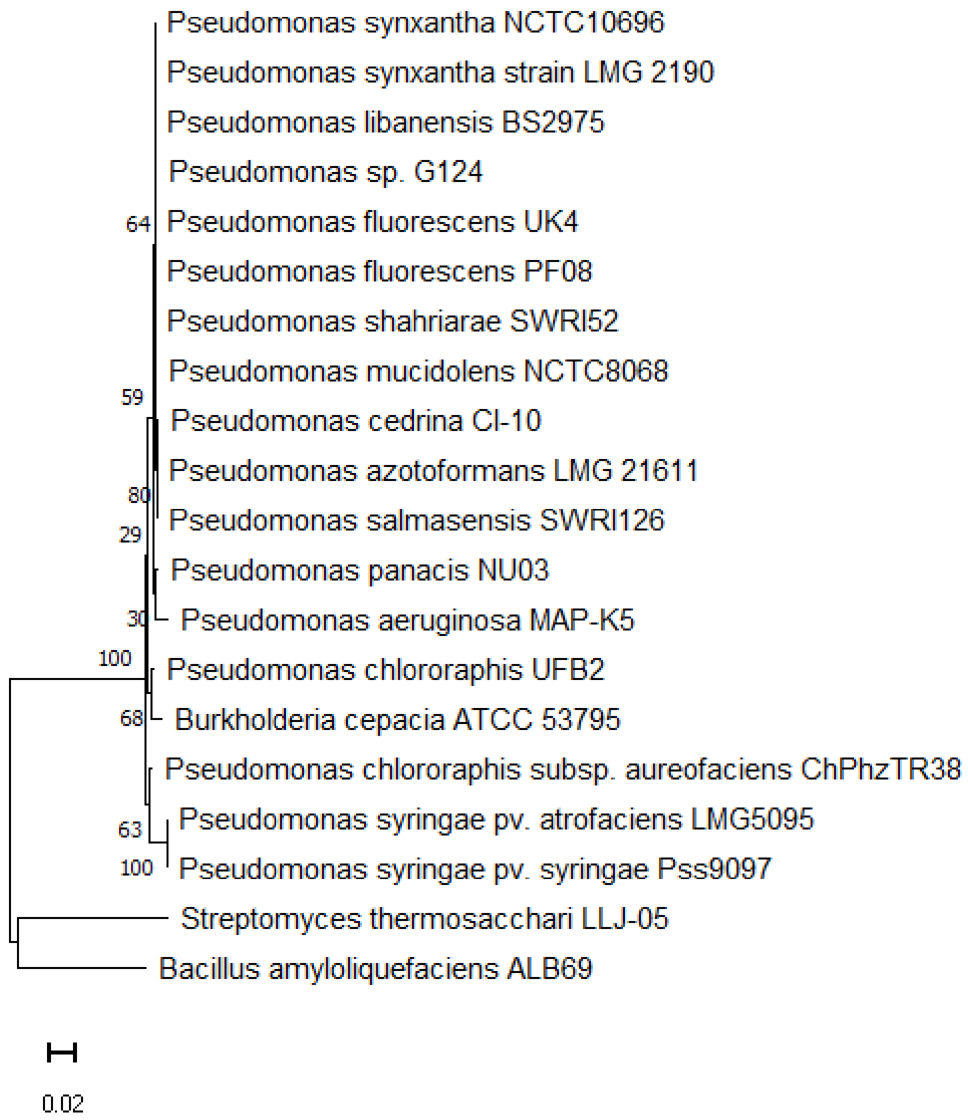

Figure S2: Neighbour-joining tree [1] of *Pseudomonas* sp. G124 and other *Pseudomonas* spp. The sequences of *Bacillus amyloliquefaciens* ALB69 and *Streptomyces theromosacchari* LLJ-05 served as outgroup. Sequences to construct the tree were aligned using the muscle algorithm [2]. Bootstrap (1000 replications) values [3] are given at the branching points. Phylogenetic analysis was performed using Mega 11 [4]. The tree is drawn to scale, with branch lengths in the same units as those of the evolutionary distances used to infer the phylogenetic tree. The evolutionary distances were computed using the Maximum Composite Likelihood method and are in the units of the number of base substitutions per site (scale bar).

Activity of *Bacillus* sp. G2112 against plant pathogens

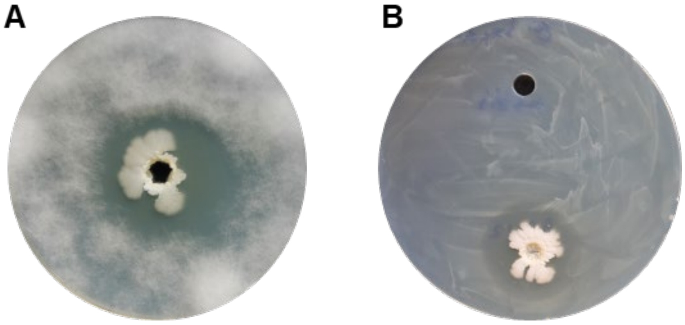

Figure S3: Bioassays of *Bacillus* sp. G2112 against (**A**) *Fusarium equiseti* and (**B**) *Erwinia tracheiphila* (*Bacillus* sp. G2112 below, sterile medium control above). Images taken after 2 d incubation at 28 °C.

Activity of *Pseudomonas* sp. G124 against plant pathogens

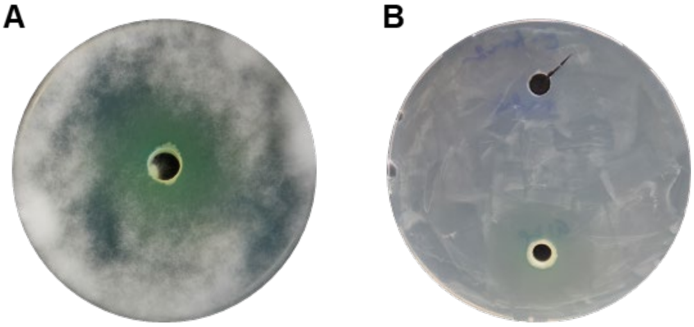

Figure S4: Bioassays of *Pseudomonas* sp. G124 against (**A**) *Fusarium equiseti* and (**B**) *Erwinia tracheiphila* (*Pseudomonas* sp. G124 below, sterile medium control above). Images taken after 2 d incubation at 28 °C.

Conditions for red pigment formation

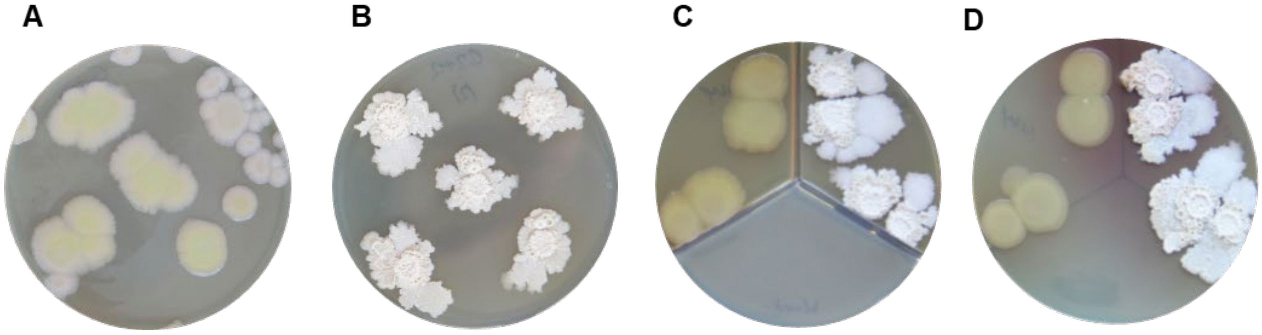

Figure S5: Condition for red pigment formation: The red pigment was not formed when (**A**) *Pseudomonas* sp. G124 and (**B**) *Bacillus* sp. G2112 were grown in single culture on agar plates, and (**C**) when *Pseudomonas* sp. G124 and *Bacillus* sp. G2112 were cultivated together in different compartments of a three-compartment plate. (**D**) The red pigment was produced only in co-culture without partition.

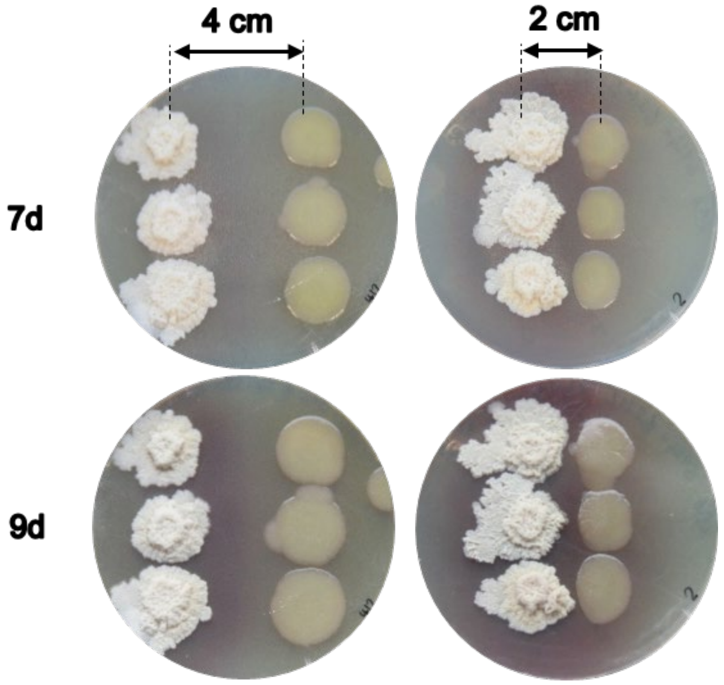

Figure S6: Effect of the distance between *Pseudomonas* sp. G124 and *Bacillus* sp. G2112 colonies on the pigment production on 5b agar. The red pigment was clearly visible after 7 d when both strains were 2 cm apart but when the colonies were 4 cm apart the pigment was only observable after 9 d of growth.

Formation of red pigments by different *Pseudomonas* and *Bacillus* strains in co-culture

Table S1: Red pigment formation in *Bacillus* and *Pseudomonas* strains in co-culture

Test organisms	Co-cultured with	Pigment produced?
Pseudomonas sp. G124	*Bacillus* sp. K13A	Yes
	Bacillus sp. K13B	Yes
	Bacillus sp. K29B	Yes
	B. pumilus DSM27	Yes
	B. subtilis DSM10	Yes
	B. thuringensis DSM2046	Yes
	B. amyloliquefaciens DSM7	Yes
Bacillus sp. G2112	*P. syingae* pv. *syringae* 22d/93	No
	P. syringae pv. *glycinea* 1a/96	No
	P. fluorescens DSM6506	No

Phenazine-1-carboxylic acid (1) as precursor for red pigments

Differential metabolite profiling of the compound causing red pigmentation

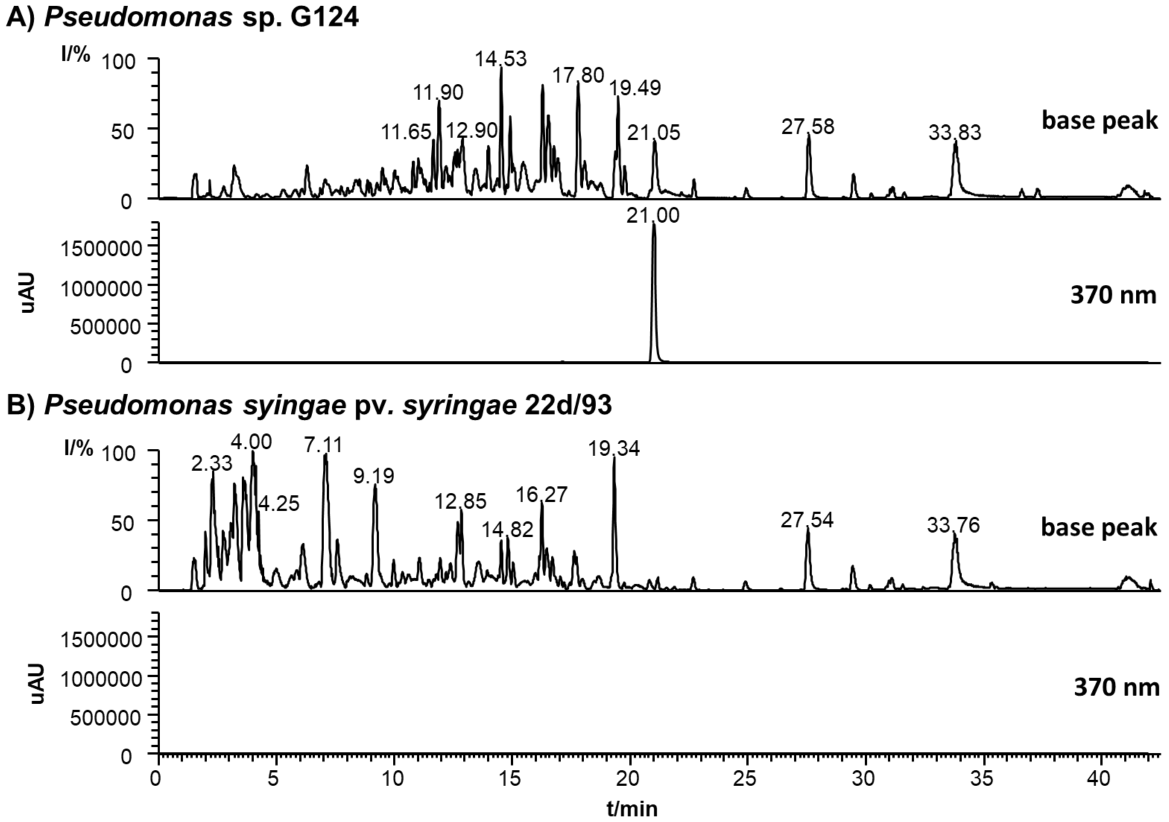

Figure S7: Comparison of the metabolite profiles (base peak ion traces and UV traces) of (**A**) *Pseudomonas* sp. G124 which caused red pigmentation around *Bacillus* sp. G2112 colonies and (**B**) *Pseudomonas syingae* pv. *syringae* 22d/93 which did not cause red pigmentation around *Bacillus* sp. G2112. At 370 nm there is a peak only present in *Pseudomonas* sp. G124 with an $[M+H]^+$ of m/z 225 corresponding to phenazine-1-carboxylic acid (**1**)

UV-Vis spectrum of phenazine-1-carboxylic acid (**1**)

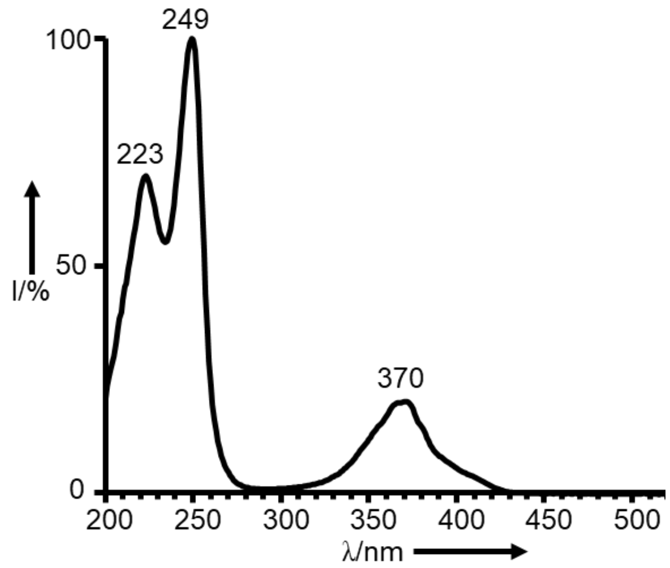

Figure S8: UV-Vis spectrum of phenazine-1-carboxylic acid (**1**)

HR-ESI-MS of phenazine-1-carboxylic acid (**1**)

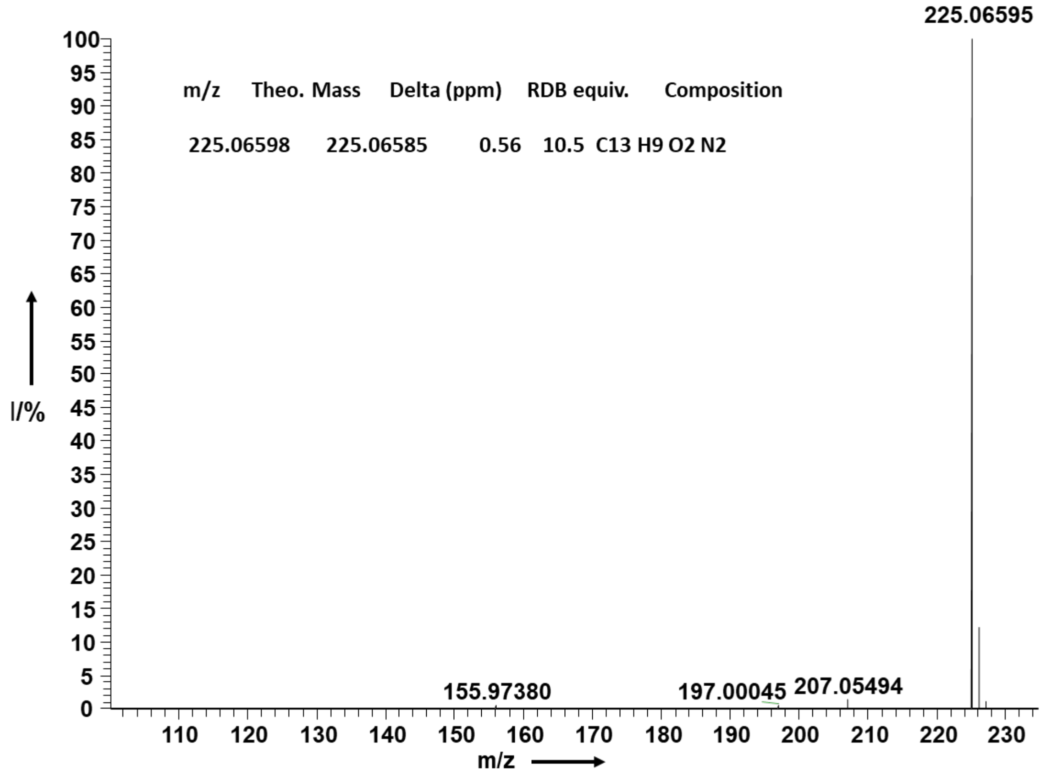

Figure S9: HR-ESI-MS of phenazine-1-carboxylic acid (**1**)

^{1}H-NMR of phenazine-1-carboxylic acid (**1**) at 600 MHz in $CDCl_3$

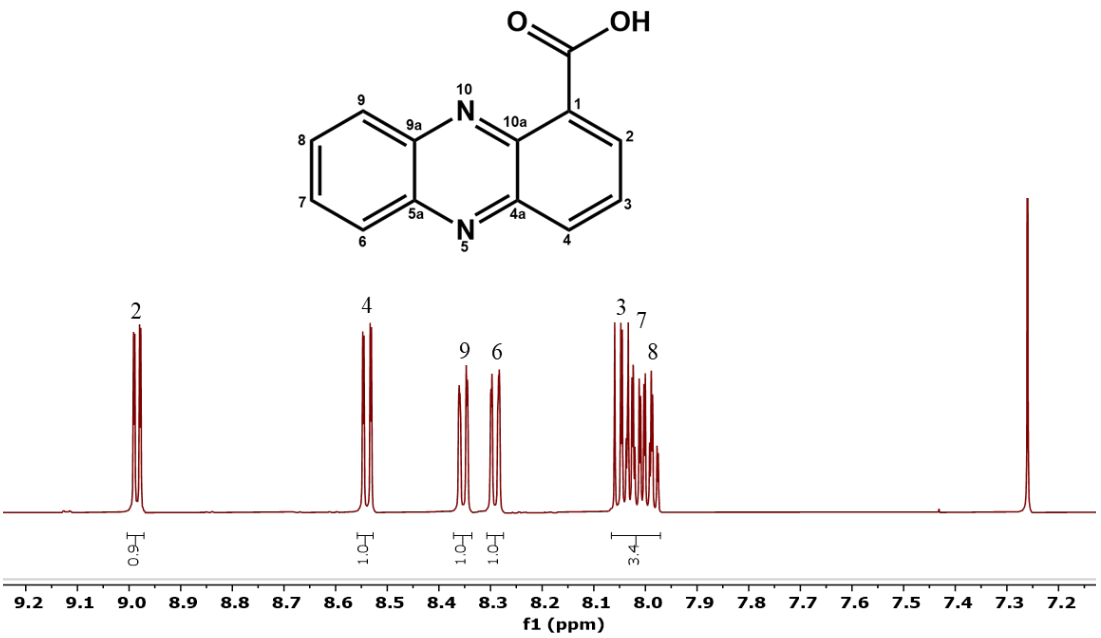

Figure S10: ^{1}H-NMR of phenazine-1-carboxylic acid (**1**)

^{13}C-NMR of phenazine-1-carboxylic acid (**1**) at 151 MHz in $CDCl_3$

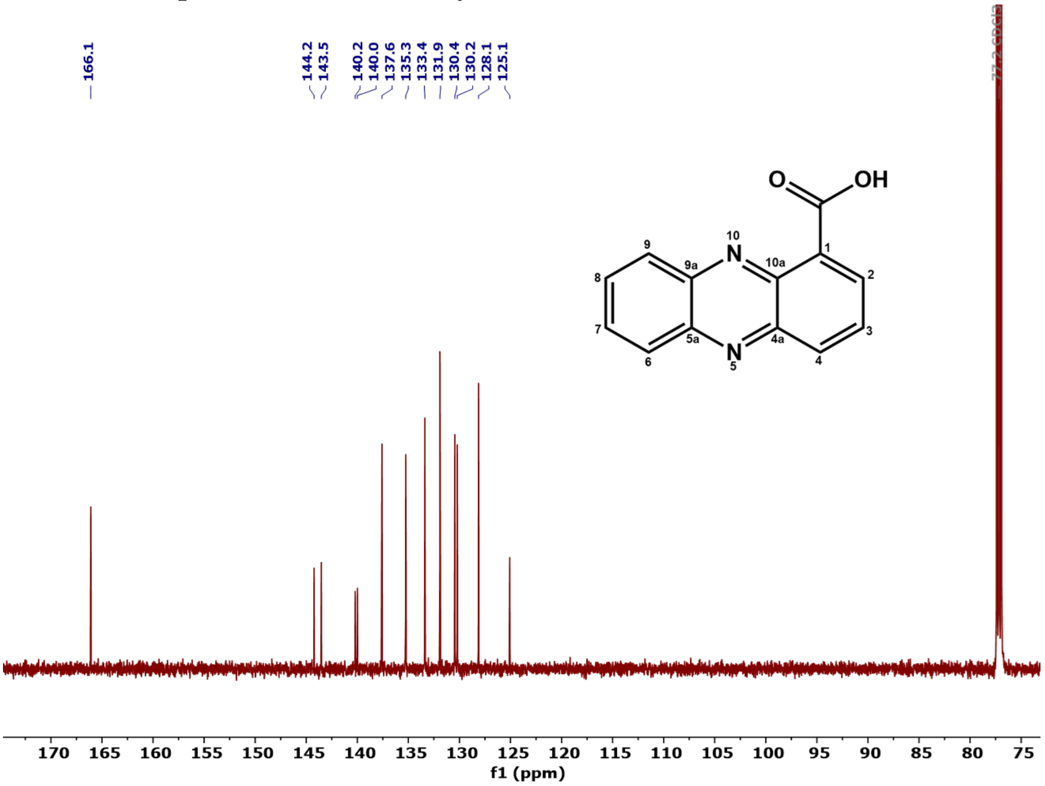

Figure S11: ^{13}C-NMR of phenazine-1-carboxylic acid (**1**) at 151 MHz in $CDCl_3$

^{1}H-^{1}H-COSY NMR of phenazine-1-carboxylic acid (**1**) at 600 MHz in $CDCl_3$

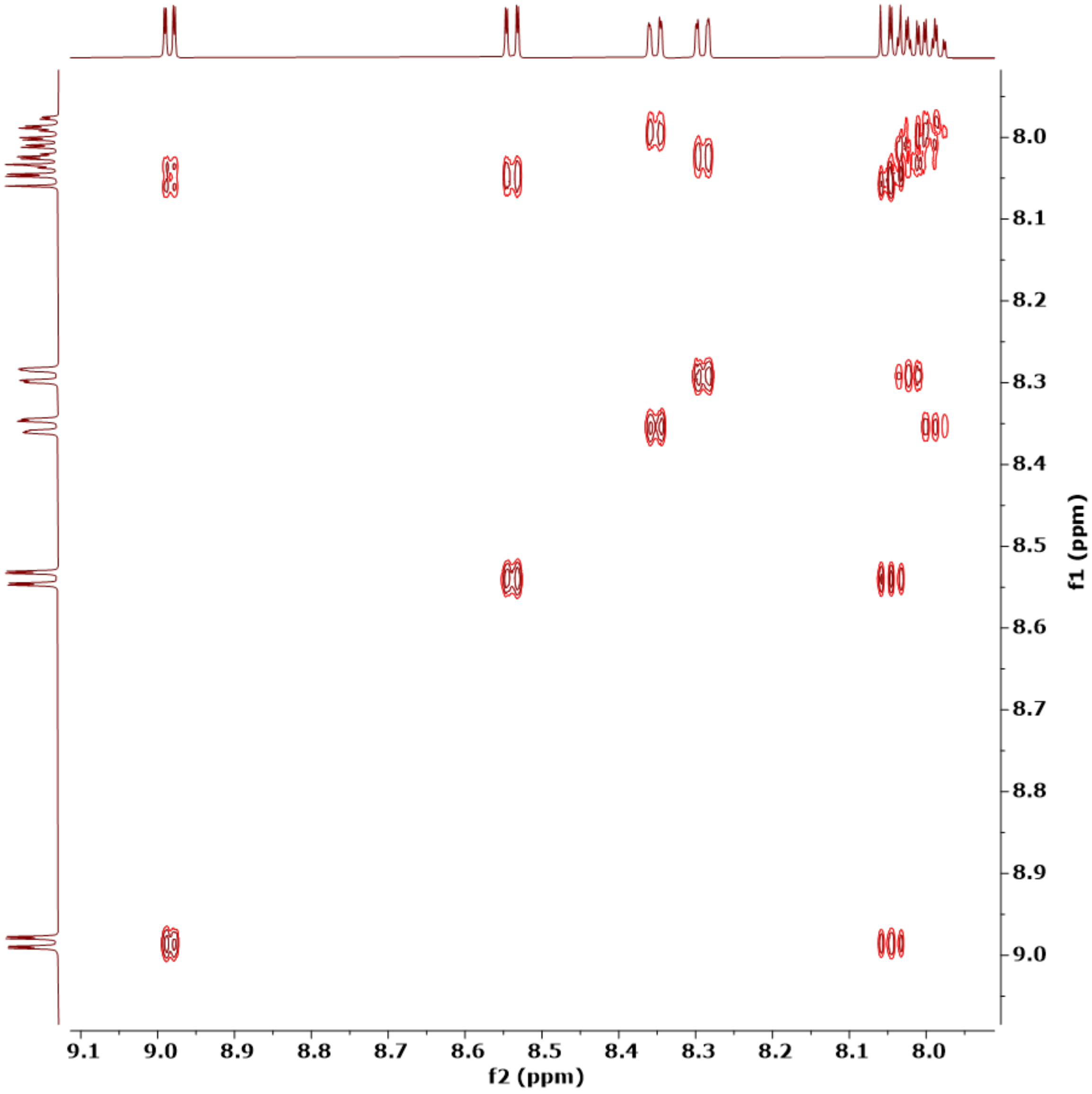

Figure S12: ^{1}H-^{1}H-COSY NMR of phenazine-1-carboxylic acid (**1**) at 600 MHz in $CDCl_3$

^{1}H-^{13}C-HSQC NMR of phenazine-1-carboxylic acid (**1**) at 600 MHz in $CDCl_3$

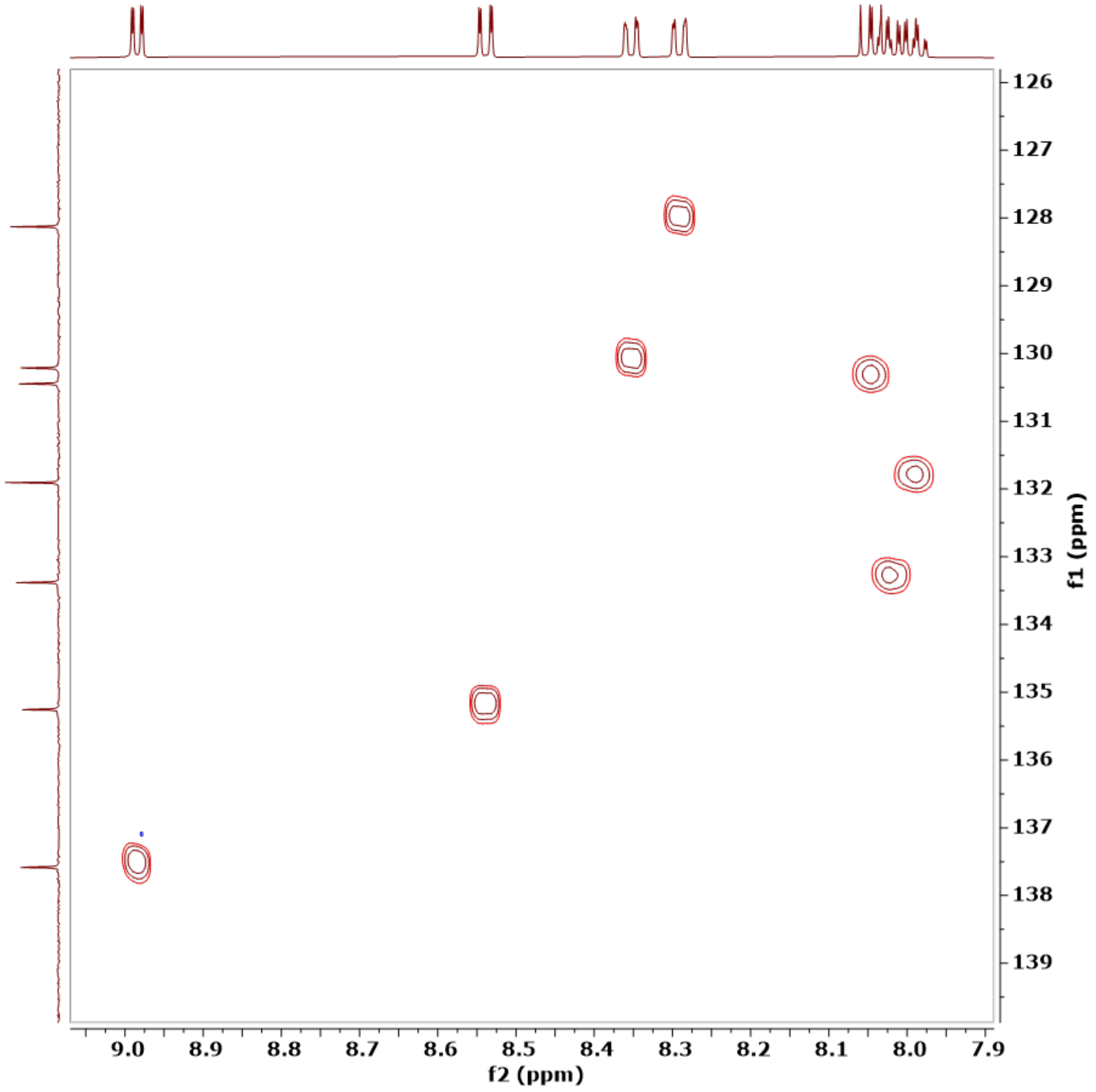

Figure S13: ^{1}H-^{13}C-HSQC NMR of phenazine-1-carboxylic acid (**1**) at 600 MHz in $CDCl_3$

^{1}H-^{13}C-HMBC NMR of phenazine-1-carboxylic acid (**1**) at 600 MHz in $CDCl_3$

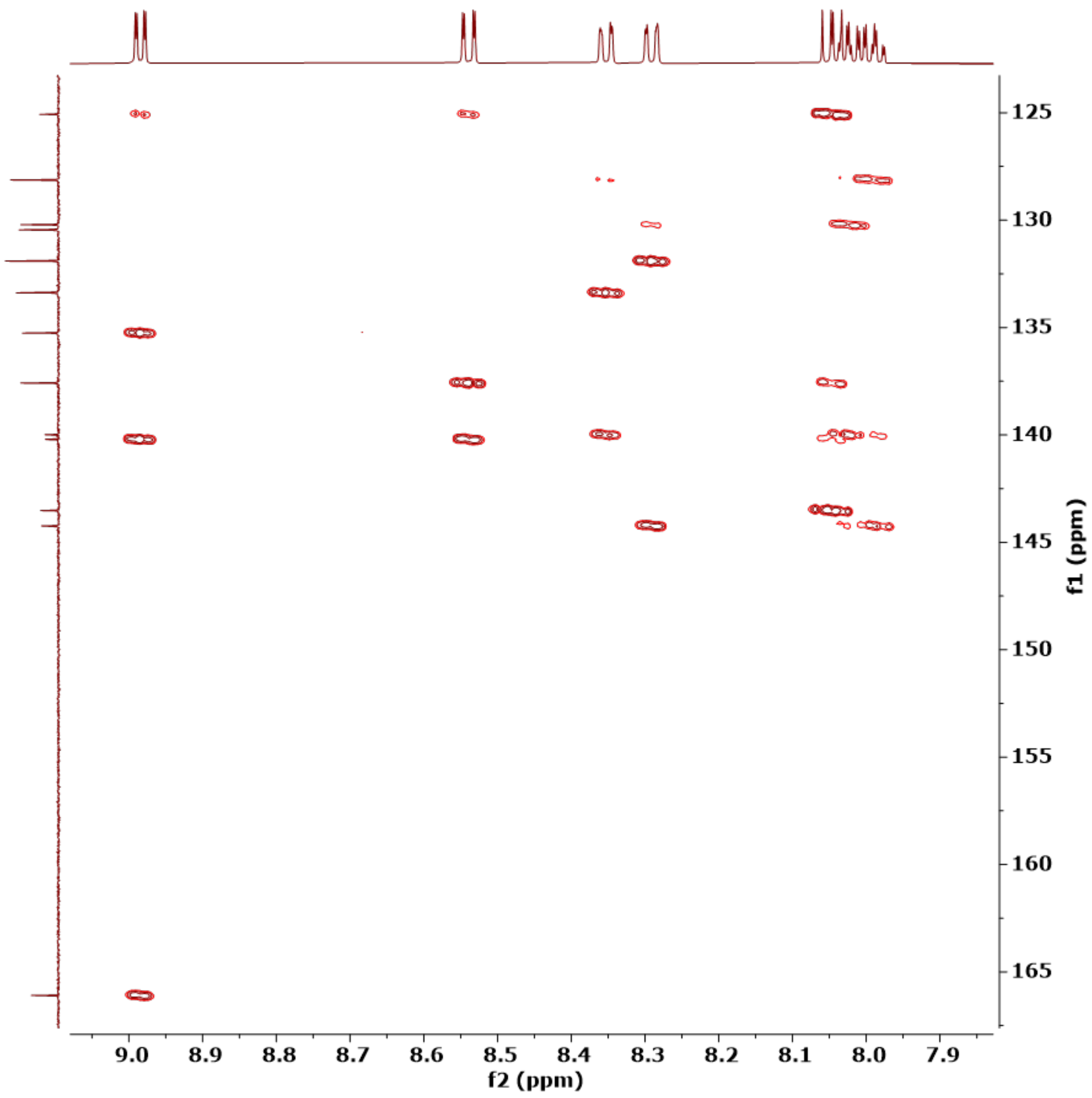

Figure S14: ^{1}H-^{13}C-HMBC NMR of phenazine-1-carboxylic acid (**1**) at 600 MHz in $CDCl_3$

***Bacillus* sp. G2112 growth and pigment production in response to phenazine-1-carboxylic acid (PCA,1)**

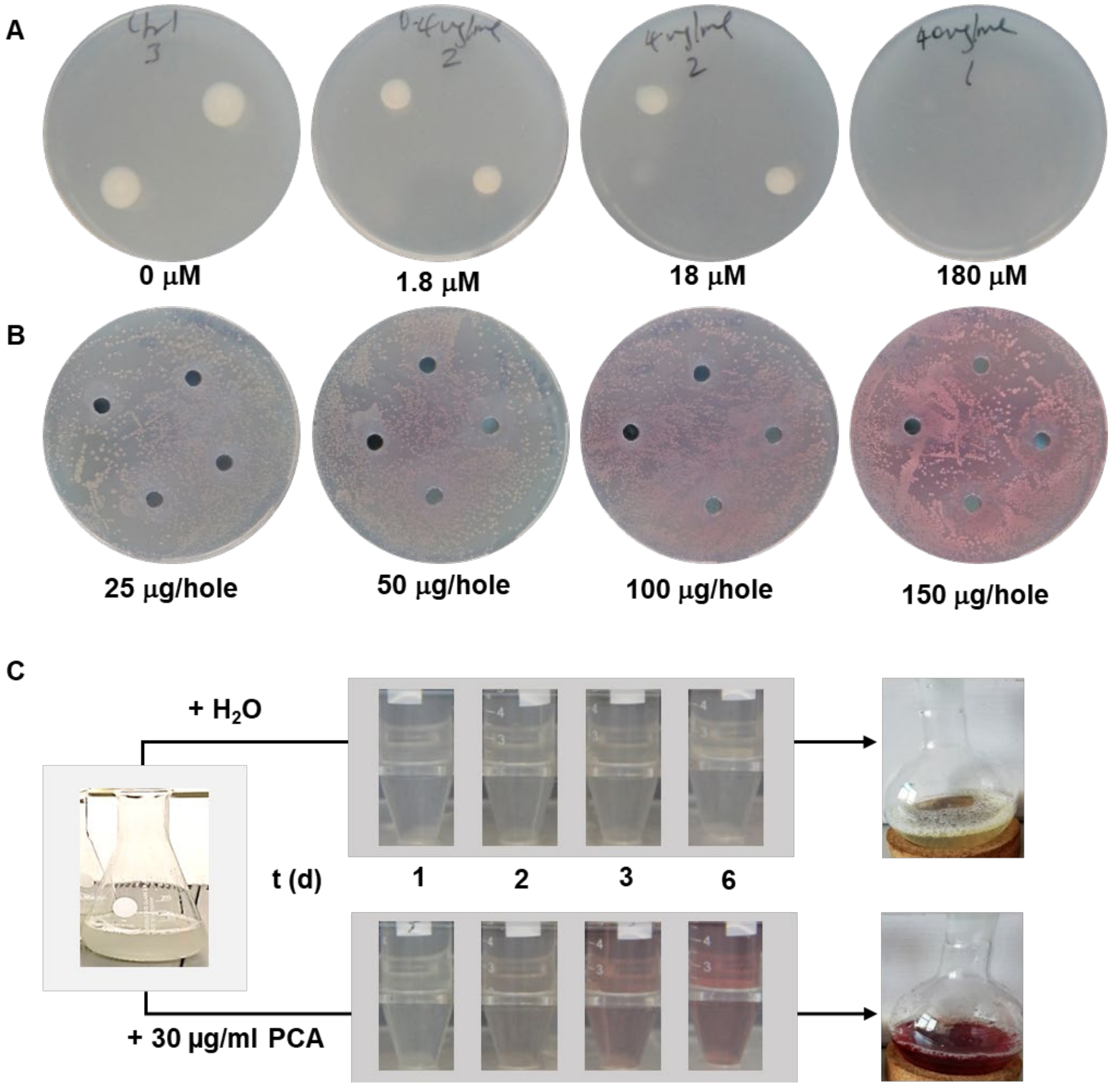

Figure S15: Effect of different PCA (**1**) concentrations on the growth of *Bacillus* sp. G2112 and its production of red pigments. (**A**) With increasing concentration of PCA (**1**) from 1.8 - 18 mM in the medium, the colony size of *Bacillus* sp. G2112 decreased. At 180 mM (40 μg/ml) PCA (**1**), *Bacillus* sp. G2112 did not grow. (**B**) The red pigmentation increased with increasing amount of PCA (**1**) from 25 - 150 μg per hole. All agar assays were performed using 5b glycerol medium. (**C**) Conversion of PCA (**1**) (134 μM) to red pigments in 5b+Gly liquid medium over time. Red coloration of the cultures became obvious at day 3.

Conversion of phenazine-1-carboxylic acid (1) to imino-5*N*-(1′β-D-glucosyl)-dihydrophenazine-1-carboxylic acids 2 and 3

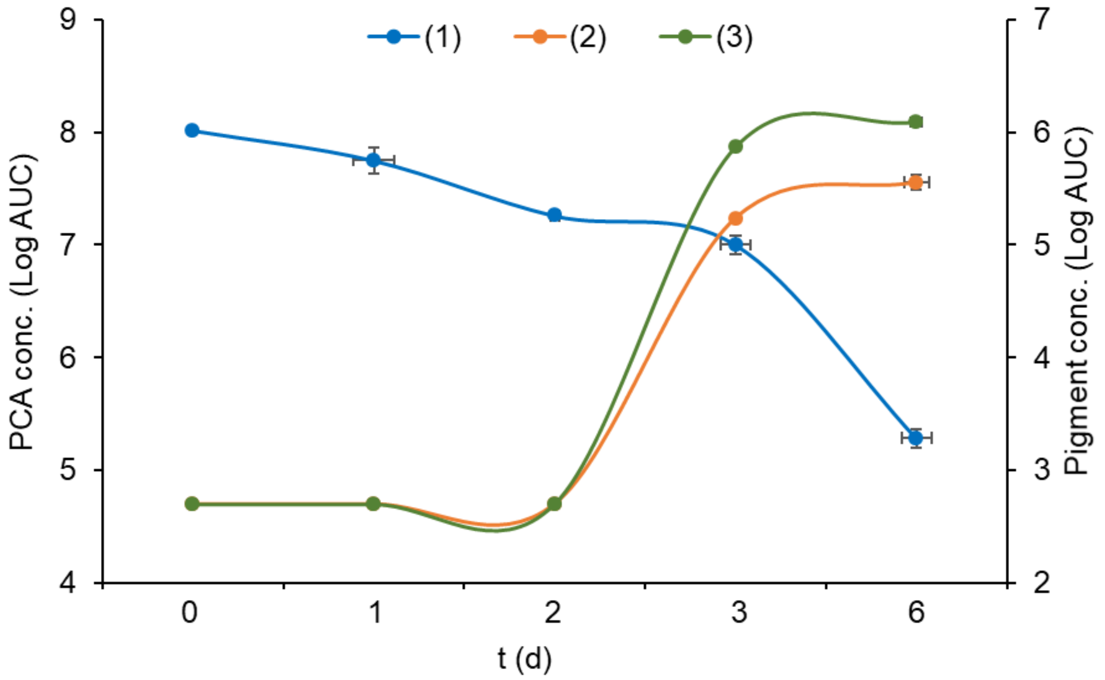

Figure S16: Conversion of PCA (**1**) to red pigments **2** and **3** by *Bacillus* sp. G2112. The concentration of PCA (**1**) decreased and concentration of two red pigments **2** (orange) and **3** (green) increased with time. Plots represent mean values and standard deviation (error bars) calculated as log of area under curve for LC-MS peaks of the individual compounds. Three biological replicates were measured.

Structure elucidation of 7-imino-5*N*-(1′β-D-glucosyl)-5,7-dihydrophenazine-1-carboxylic acid (2)

UV-Vis spectrum of 7-imino-5*N*-(1′β-D-glucosyl)-5,7-dihydrophenazine-1-carboxylic acid (**2**)

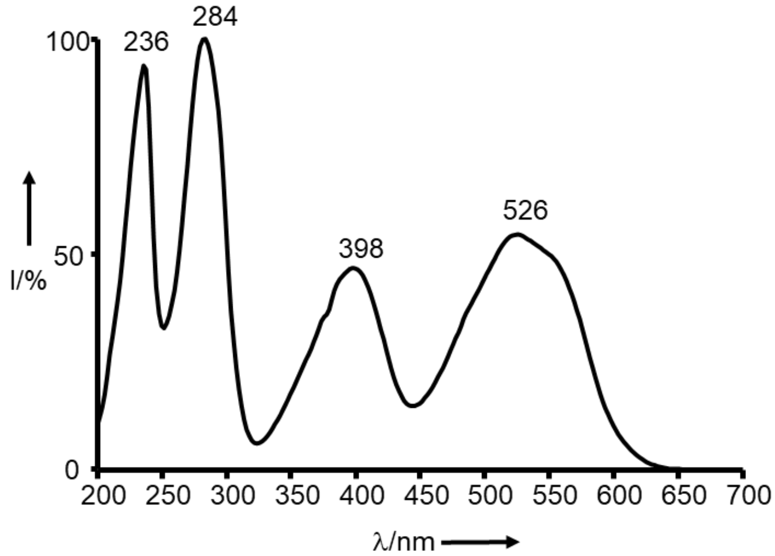

Figure S17: UV-Vis spectrum of 7-imino-5*N*-(1′β-D-glucopyranosyl)-5,7-dihydrophenazine-1-carboxylic acid (**2**).

HR-ESI-MS of 7-imino-5*N*-(1′β-D-glucopyranosyl)-5,7-dihydrophenazine-1-carboxylic acid (**2**)

m/z	Theo. Mass	Delta (ppm)	RDB equiv.	Composition
402.12948	402.12958	-0.24	11.5	$C_{19}H_{20}O_7N_3$

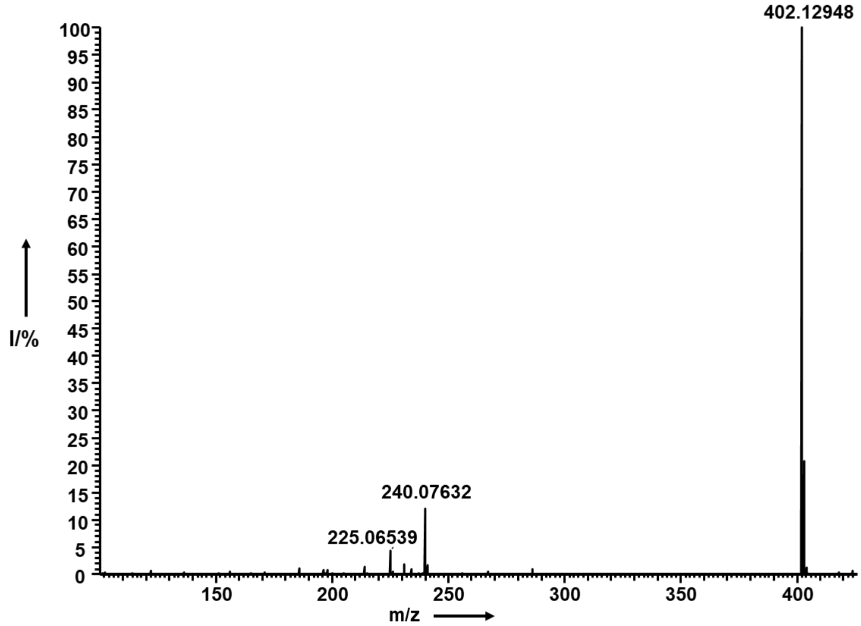

Figure S18: HR-ESI-MS spectrum of 7-imino-5*N*-(1′β-D-glucopyranosyl)-5,7-dihydrophenazine-1-carboxylic acid (**2**).

HR-ESI-MS/MS of 7-imino-5*N*-(1′β-D-glucopyranosyl)-5,7-dihydrophenazine-1-carboxylic acid (**2**)

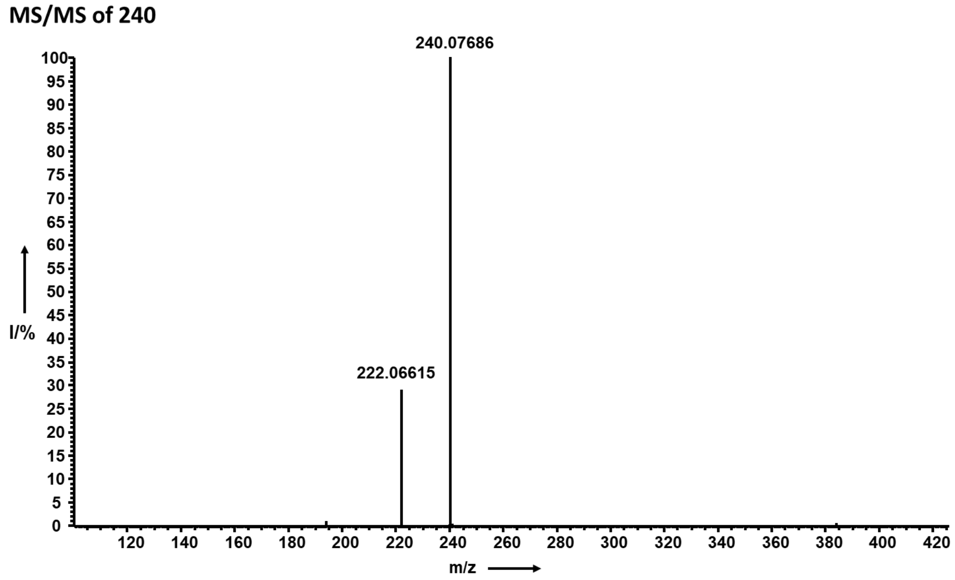

Figure S19: HR-ESI-MS/MS spectrum of 7-imino-5*N*-(1′β-D-glucopyranosyl)-5,7-dihydrophenazine-1-carboxylic acid (**2**).

^{1}H-NMR of 7-imino-5*N*-(1′β-D-glucosyl)-5,7-dihydrophenazine-1-carboxylic acid (**2**) at 600 MHz in CD_3OD

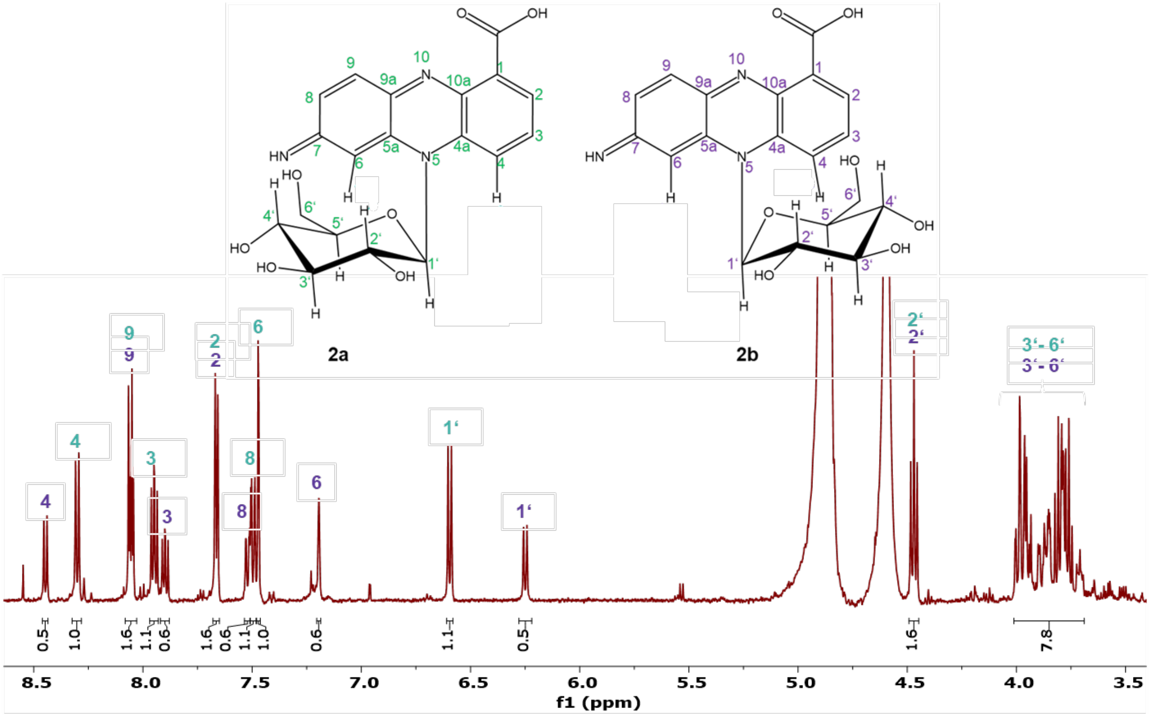

Figure S20: ^{1}H-NMR spectrum of 7-imino-5*N*-(1′β-D-glucopyranosyl)-5,7-dihydrophenazine-1-carboxylic acid (**2**). Atom numbers of major isomer (**2a**) are shown in green and atom numbers of the minor isomer (**2b**) in purple.

^{13}C-NMR of 7-imino-5*N*-(1′β-D-glucopyranosyl)-5,7-dihydrophenazine-1-carboxylic acid (**2**) at 151 MHz in CD_3OD

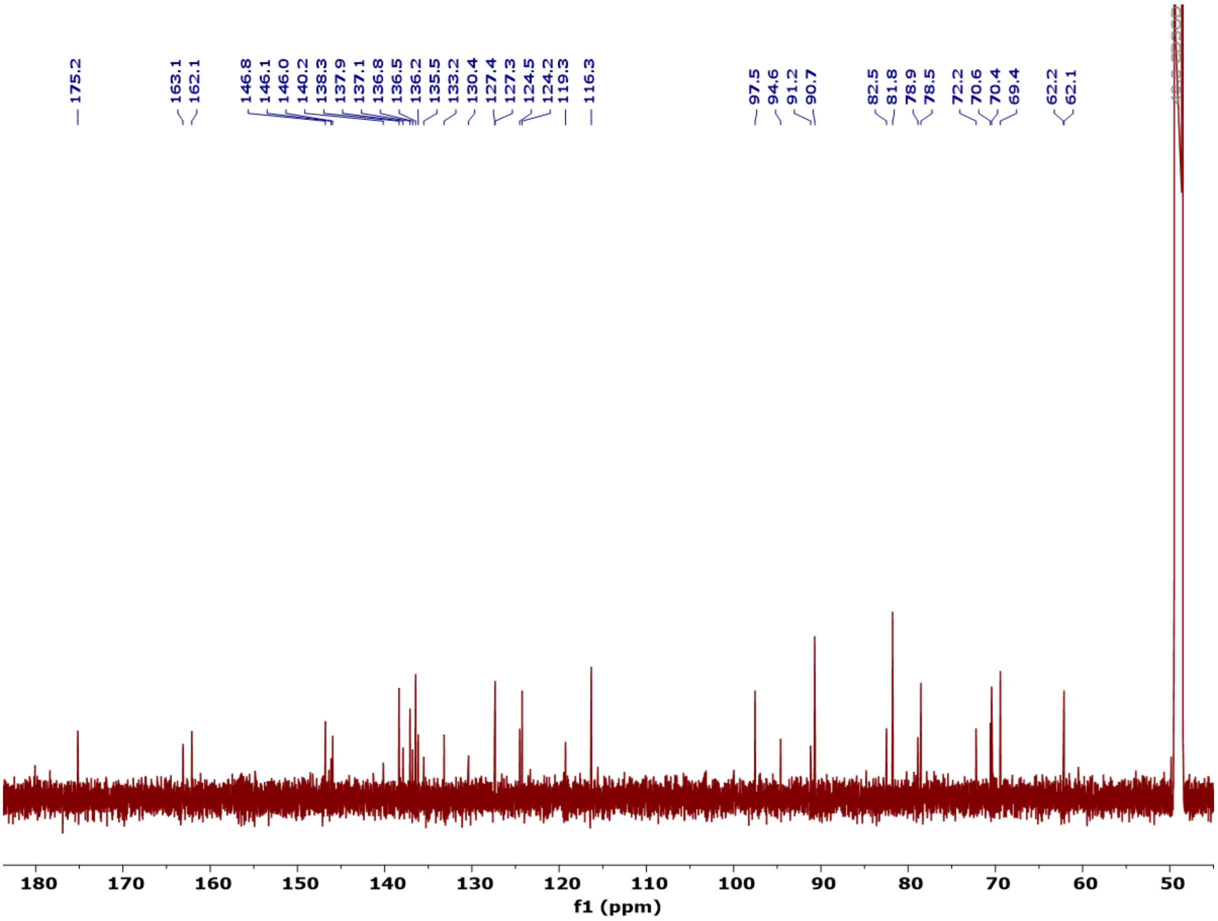

Figure S21: ^{13}C-NMR spectrum of 7-imino-5*N*-(1′β-D-glucopyranosyl)-5,7-dihydro-phenazine-1-carboxylic acid (**2**).

^{1}H-^{1}H-COSY NMR of 7-imino-5*N*-(1′β-D-glucosyl)-phenazine-1-carboxylic acid (**2**) at 600 MHz in CD_3OD

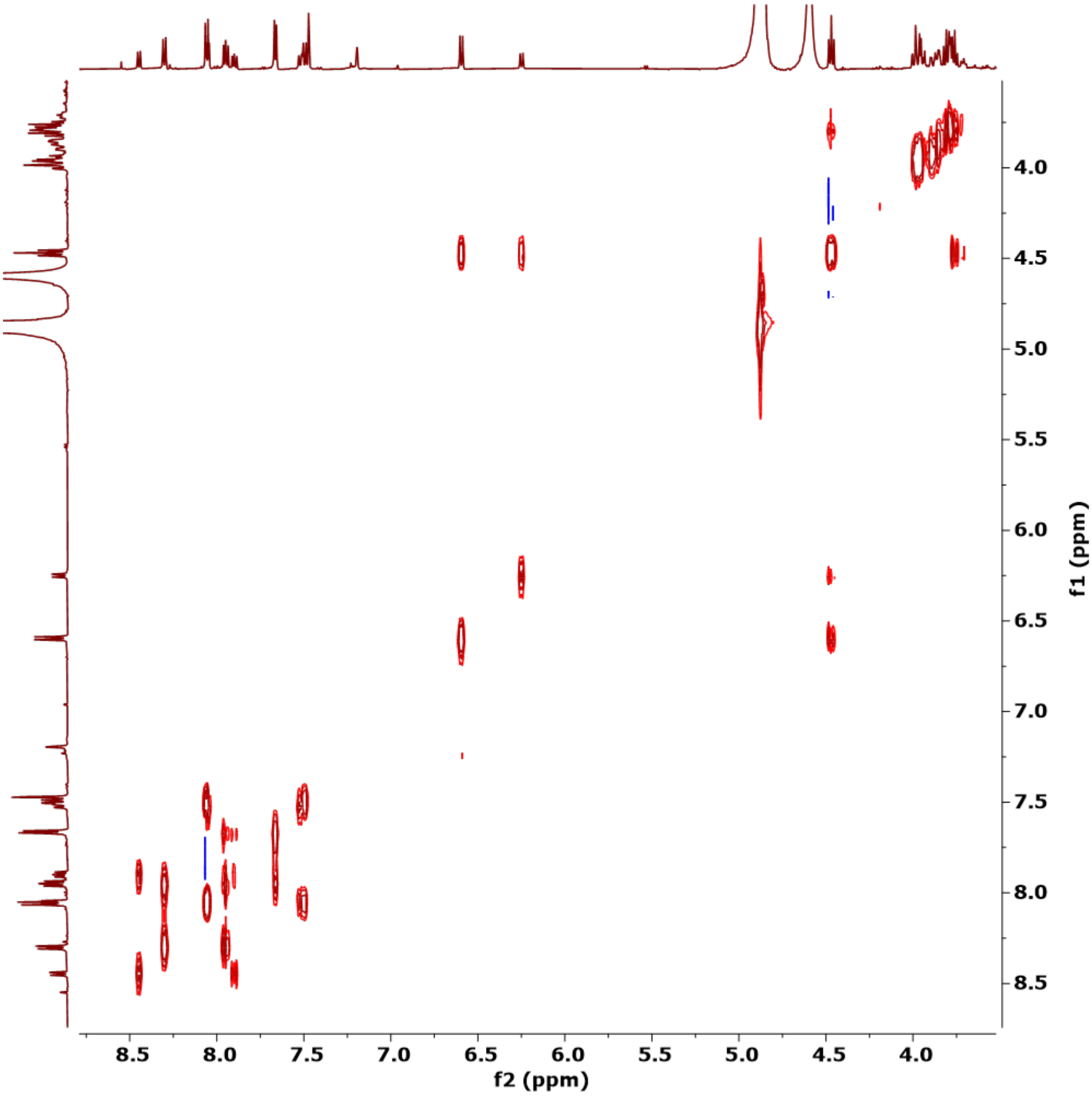

Figure S22: ^{1}H-^{1}H-COSY NMR spectrum of 7-imino-5*N*-(1′β-D-glucopyranosyl)-5,7-dihydrophenazine-1-carboxylic acid (**2**).

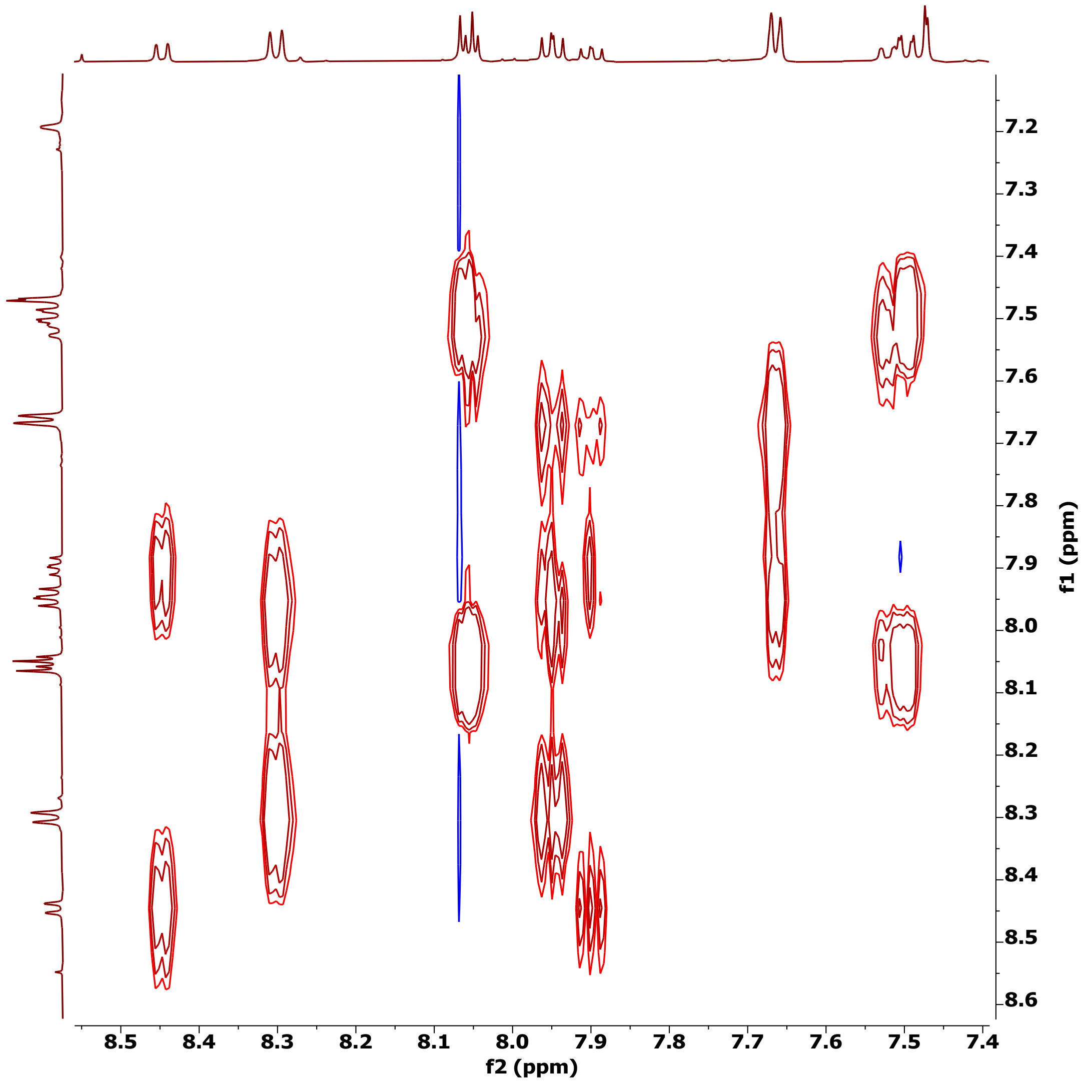

Figure S23: ^{1}H-^{1}H-COSY NMR spectrum of the aromatic region of 7-imino-5*N*-(1′β-D-glucopyranosyl)-5,7-dihydrophenazine-1-carboxylic acid (**2**).

^{1}H-^{13}C-HSQC NMR of 7-imino-5*N*-(1′β-D-glucopyranosyl)-5,7-dihydrophenazine-1-carboxylic acid (**2**) at 600 MHz in CD_3OD

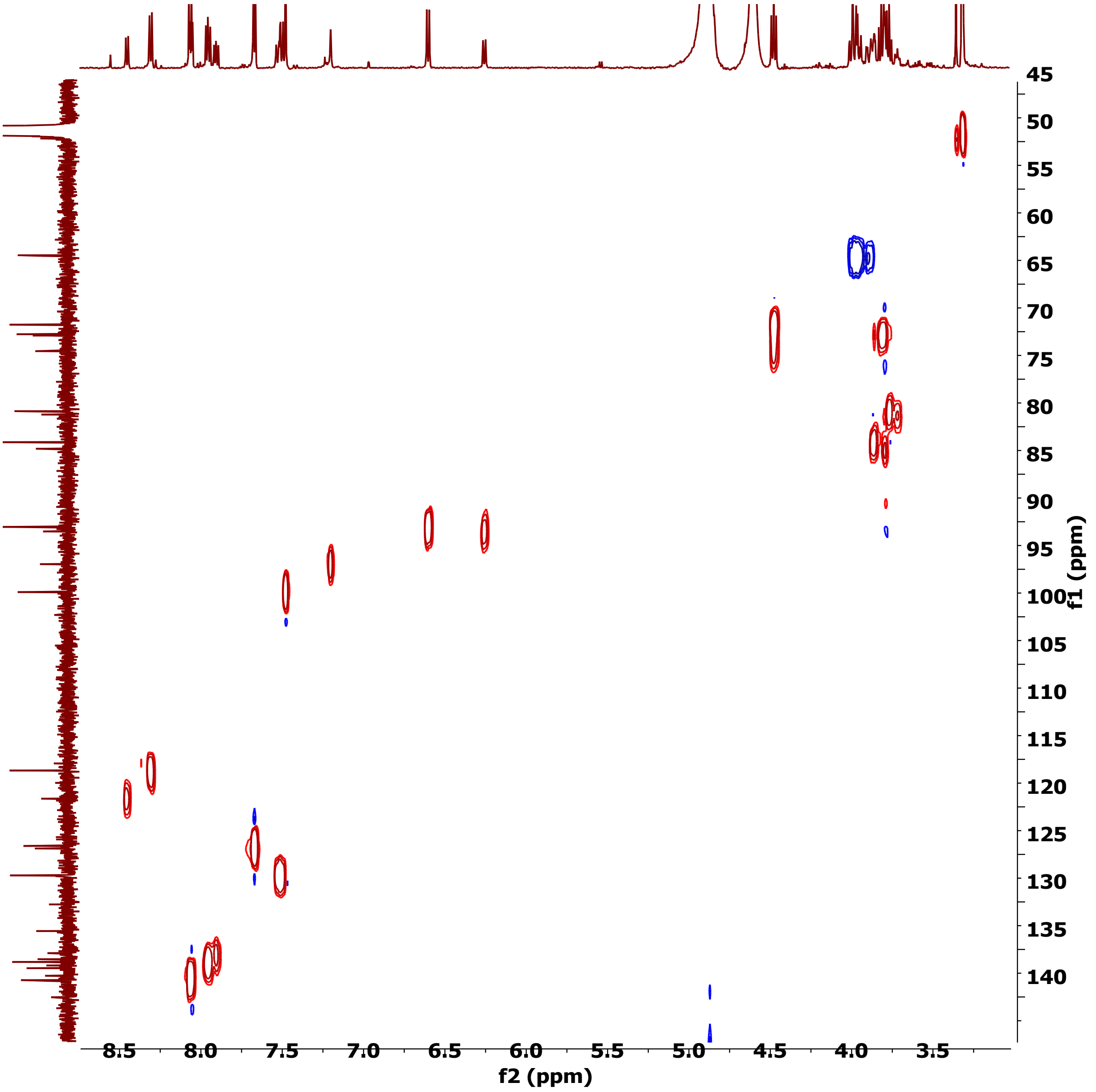

Figure S24: ^{1}H-^{13}C-HSQC NMR spectrum of the aromatic region of 7-imino-5*N*-(1′β-D-glucopyranosyl)-5,7-dihydrophenazine-1-carboxylic acid (**2**).

^{1}H-^{13}C-HMBC NMR of 7-imino-5*N*-(1′β-D-glucopyranosyl)-5,7-dihydrophenazine-1-carboxylic acid (**2**) at 600 MHz in CD_3OD

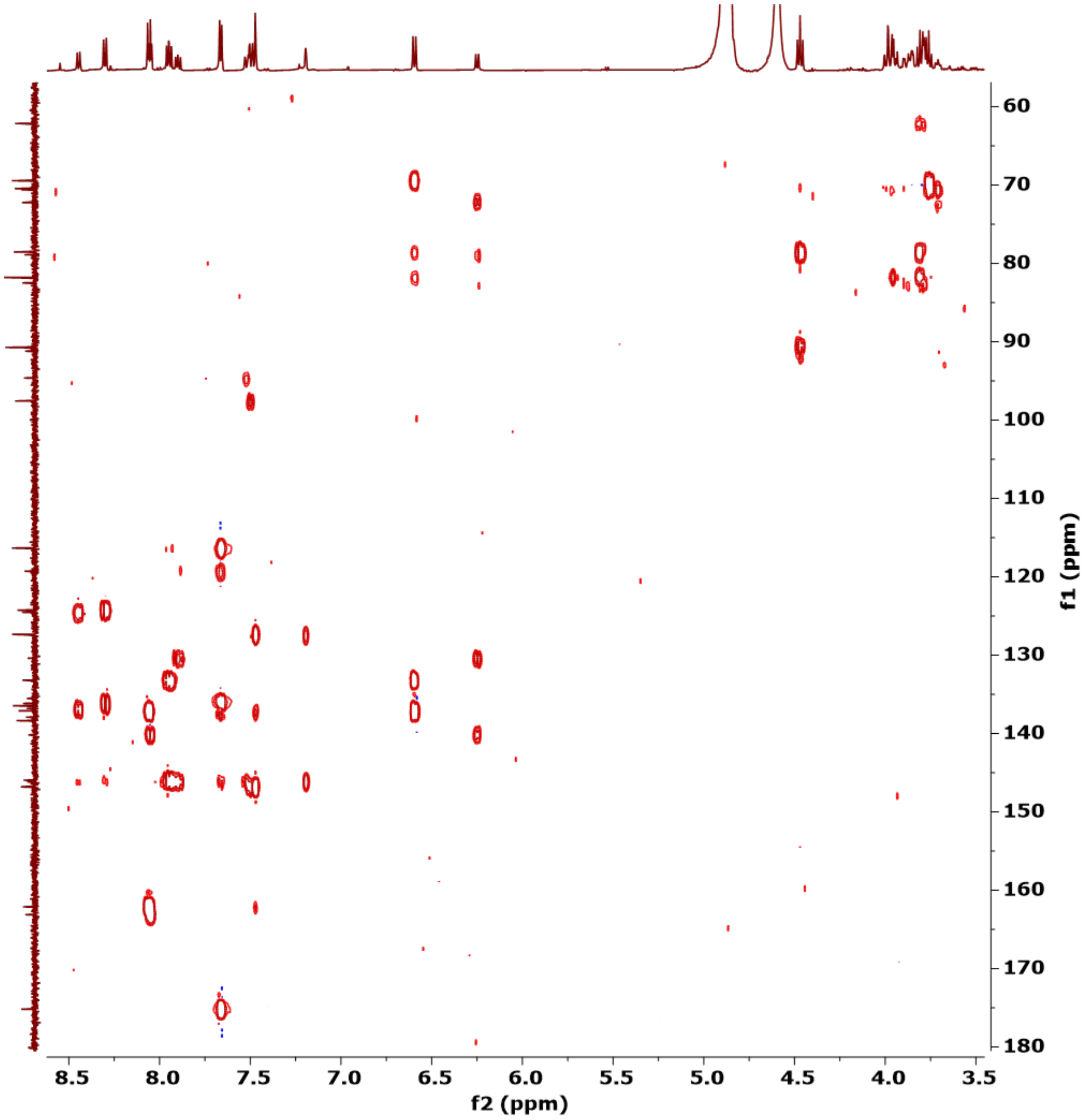

Figure S25: ^{1}H-^{13}C HMBC NMR spectrum of the aromatic region of 7-imino-5*N*-(1′β-D-glucopyranosyl)-5,7-dihydrophenazine-1-carboxylic acid (**2**).

^{1}H-^{1}H-COSY and ^{1}H-^{13}C-HMBC correlations of 7-imino-5*N*-(1′β-D-glucopyranosyl)-5,7-dihydrophenazine-1-carboxylic acid (**2**)

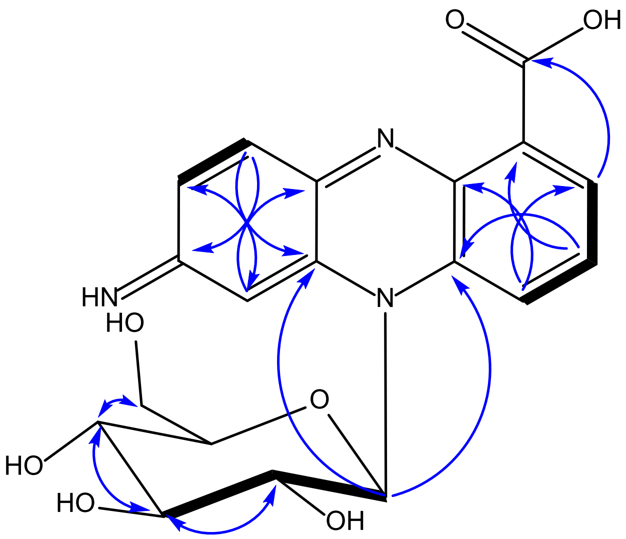

Figure S26: Structure of 7-imino-5*N*-(1′β-D-glucopyranosyl)-5,7-dihydrophenazine-1-carboxylic acid (**2**). ^{1}H-^{1}H-COSY correlations are indicated by thick bonds and ^{1}H-^{13}C-HMBC correlations are indicated by blue arrows.

NMR data of 7-imino-5*N*-(1′β-D-glucopyranosyl)-5,7-dihydrophenazine-1-carboxylic acid (**2**)

Table S2: ^{1}H-NMR (600 MHz, CD_3OD) data for 7-imino-5*N*-(1′β-D-glucopyranosyl)-5,7-dihydrophenazine-1-carboxylic acid (**2a** and **2b**).

**Position**	**^{1}H-NMR of 2a** shift (ppm) /multiplicity/coupling constant *J* (Hertz)	**^{13}C-NMR of 2a** shift (ppm)	**^{1}H-NMR of 2b** shift (ppm) /multiplicity/coupling constant *J* (Hertz)	**^{13}C-NMR of 2b** shift (ppm)
COOH		175.2 qC		175.2 qC
1		146.0 qC		146.1 qC
2	7.66, d, 7.0	124.2, CH	7.66, d, 7.1	124.5, CH
3	7.95, dd, 9.0, 7.1	136.4, CH	7.90, dd, 8.9, 7.1	135.5, CH
4	8.30, d, 9.0	116.3, CH	8.45, d, 8.9	119.3, CH
4a		133.2, qC		130.4, qC
5a		137.1, qC		140.1, qC
6	7.47, d, 2.2	97.5, CH	7.19, d, 2.0	94.6, CH
7	-	162.1, qC	-	163.1, qC
8	7.50, dd, 9.4, 2.2	127.3, CH	7.52, dd, 9.4, 1.9	127.4, CH
9	8.06, d, 9.4	138.3, CH	8.05, d, 9.4	137.9, CH
9a		146.8, qC		146.1, qC
10a		136.2, qC		136.8, qC
1'	6.60, d, 9.4	90.7, CH	6.25, d, 9.4	91.2, CH
2'	4.47, t, 8.9	69.4, CH	4.47, t, 8.9	72.2, CH
3'	3.74-3.78, m[a]	78.5, CH	3.70-3.73, m [a]	78.9, CH
4'	3.78-3.83, m[a]	70.3, CH	3.75-3.78, m [a]	70.5, CH
5'	3.83-3.87, m[a]	81.7, CH	3.77-3.80, m [a]	82.5, CH
6'	3.92-4.01, m[a]	62.1, CH_2	3.88-3.94, m [a]	62.2, CH_2

[a] overlap between signal of both isomers, range determined by ^{1}H-^{1}H-COSY cross signals.

Structure elucidation of 3-imino-5*N*-(1′β-D-glucopyranosyl)-3,5-dihydro-phenazine-1-carboxylic acid (3)

UV-Vis spectrum of 3-imino-5*N*-(1′β-D-glucopyranosyl)-3,5-dihydrophenazine-1-carboxylic acid (**3**)

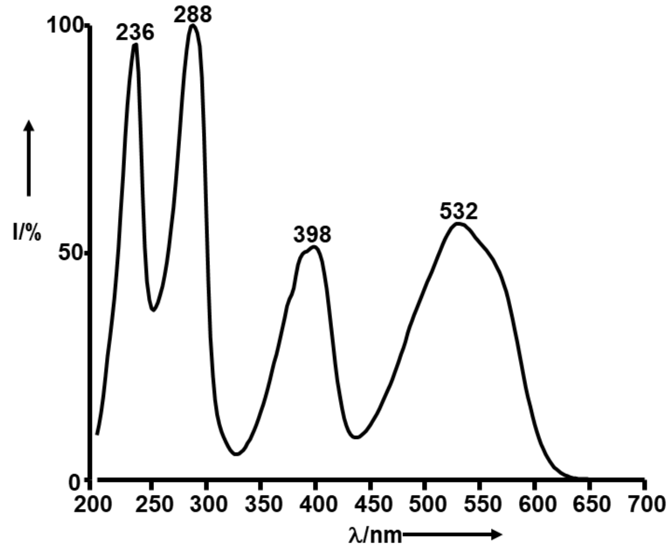

Figure S27: UV-Vis spectrum of 3-imino-5*N*-(1′β-D-glucopyranosyl)-3,5-dihydrophenazine-1-carboxylic acid (**3**).

HR-ESI-MS of 3-imino-5*N*-(1′β-D-glucopyranosyl)-3,5-dihydrophenazine-1-carboxylic acid (**3**)

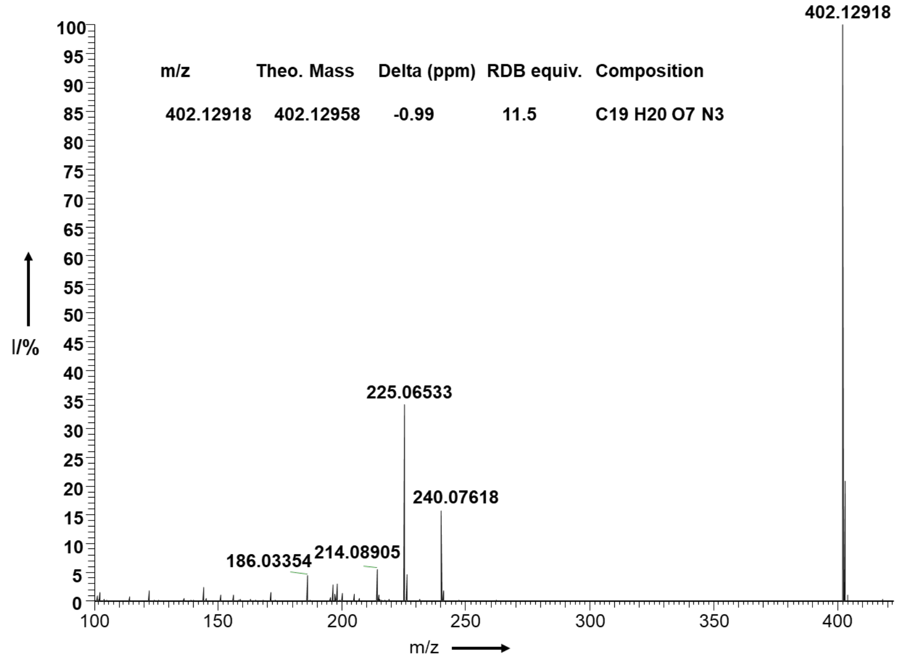

Figure S28: HR-ESI-MS of 3-imino-5*N*-(1′β-D-glucopyranosyl)-3,5-dihydrophenazine-1-carboxylic acid (**3**).

HR-ESI-MS/MS of 3-imino-5*N*-(1′β-D-glucopyranosyl)-3,5-dihydrophenazine-1-carboxylic acid (**3**)

m/z	Theo. Mass	Delta (ppm)	RDB equiv.	Composition
240.07664	240.07675	-0.47	10.5	$C_{13} H_{10} O_2 N_3$

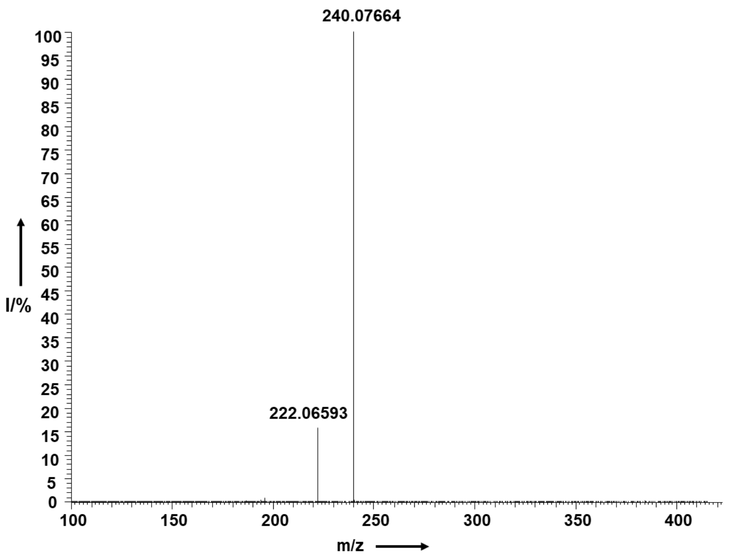

Figure S29: HR-ESI-MS/MS of 3-imino-5*N*-(1′β-D-glucopyranosyl)-3,5-dihydrophenazine-1-carboxylic acid (**3**).

H-NMR of 3-imino-5*N*-(1′β-D-glucopyranosyl)-3,5-dihydrophenazine-1-carboxylic acid (**3**) at 600 MHz in CD3OD

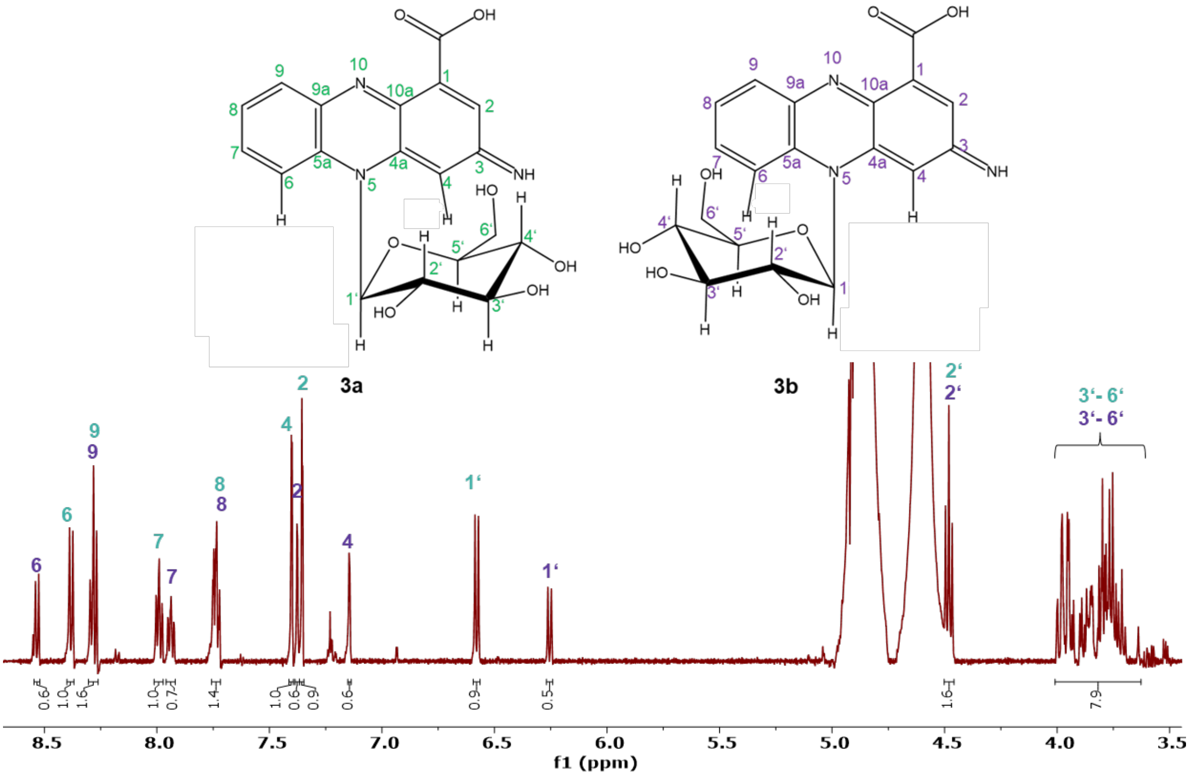

Figure S30: ^{1}H-NMR spectrum of 3-imino-5*N*-(1′β-D-glucopyranosyl)-3,5-dihydrophenazine-1-carboxylic acid (**3**). Atom numbers of major isomer (**3a**) are shown in green and atom numbers of the minor isomer (**3b**) in purple.

^{1}H-^{1}H-COSY NMR of 3-imino-5*N*-(1′β-D-glucopyranosyl)-3,5-dihydrophenazine-1-carboxylic acid (**3**) at 600 MHz in CD_3OD

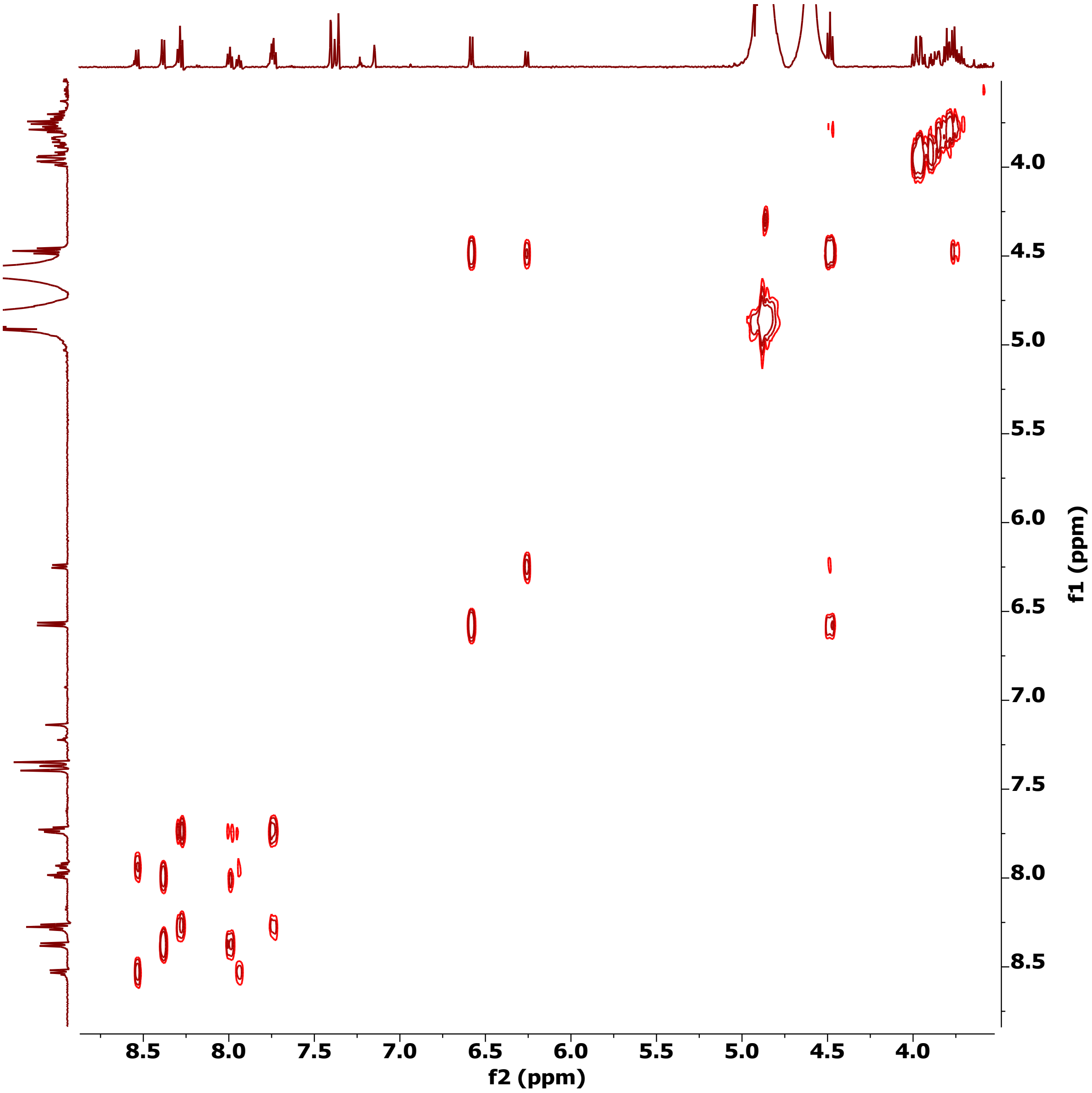

Figure S31: ^{1}H-^{1}H-COSY NMR spectrum of 3-imino-5*N*-(1′β-D-glucopyranosyl)-3,5-dihydrophenazine-1-carboxylic acid (**3**)

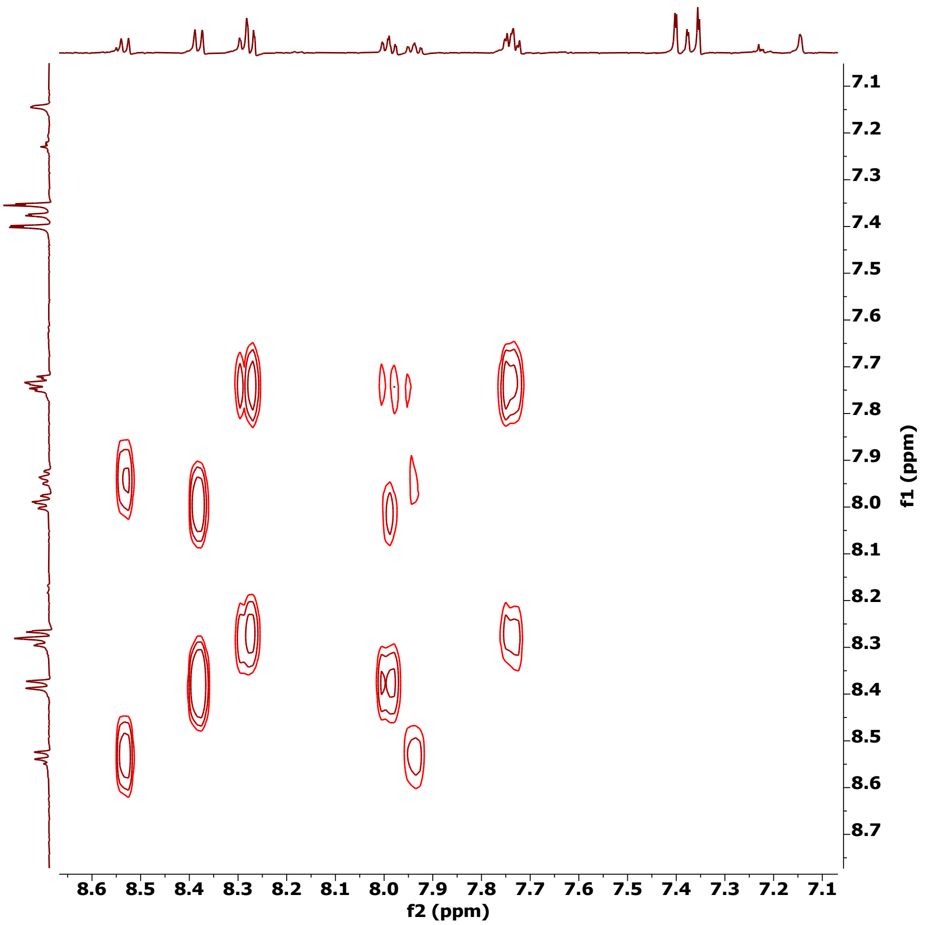

Figure S32: ^{1}H-^{1}H-COSY NMR spectrum of the aromatic region of 3-imino-5*N*-(1′β-D-glucopyranosyl)-3,5-dihydrophenazine-1-carboxylic acid (**3**)

^1H-^{13}C-HSQC NMR of 3-imino-5*N*-(1′β-D-glucopyranosyl)-3,5-dihydrophenazine-1-carboxylic acid (**3**) at 600 MHz in CD_3OD

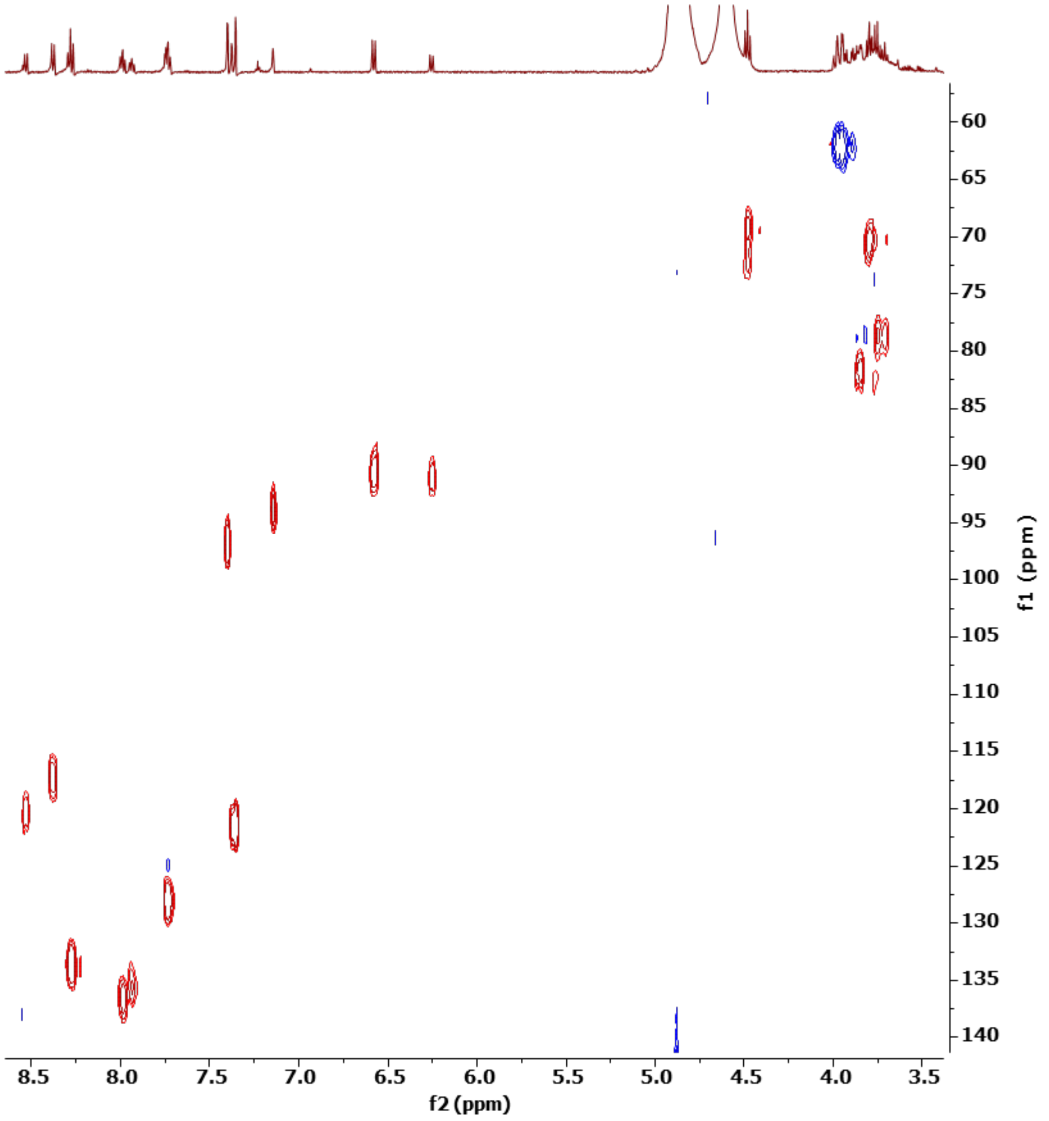

Figure S33: ^1H-^{13}C-HSQC NMR spectrum of 3-imino-5*N*-(1′β-D-glucopyranosyl)-3,5-dihydrophenazine-1-carboxylic acid (**3**)

^1H-^{13}C-HMBC NMR of 3-imino-5*N*-(1′β-D-glucopyranosyl)-3,5-dihydrophenazine-1-carboxylic acid (**3**) at 600 MHz in CD_3OD

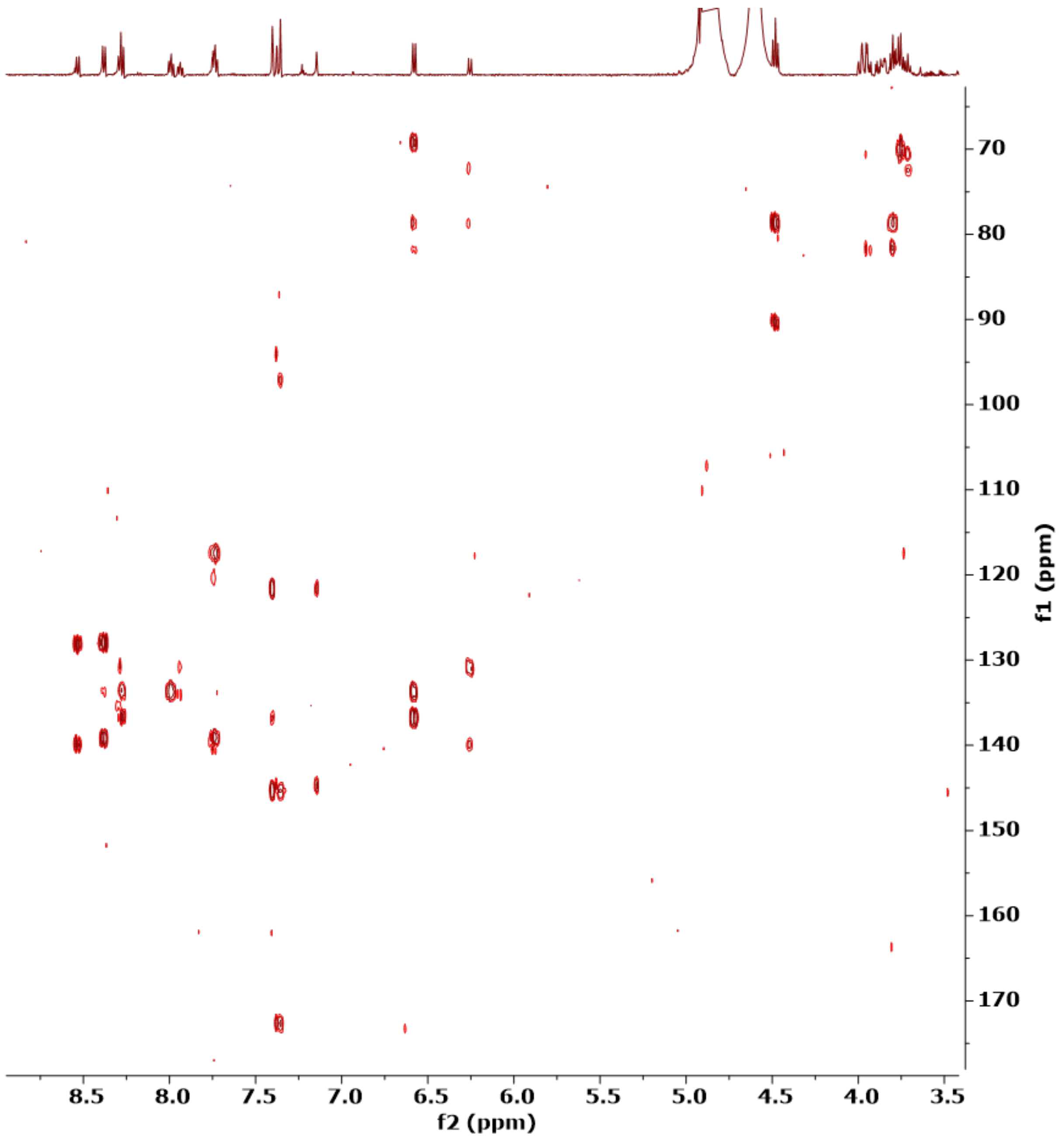

Figure S34: ^1H-^{13}C-HMBC NMR (600 MHz, CD_3OD) spectrum of 3-imino-5*N*-(1′β-D-glucopyranosyl)-3,5-dihydrophenazine-1-carboxylic acid (**3**)

^{1}H-^{1}H-COSY and ^{1}H-^{13}C-HMBC correlations of 3-imino-5*N*-(1′β-D-glucopyranosyl)-3,5-dihydrophenazine-1-carboxylic acid (**3**)

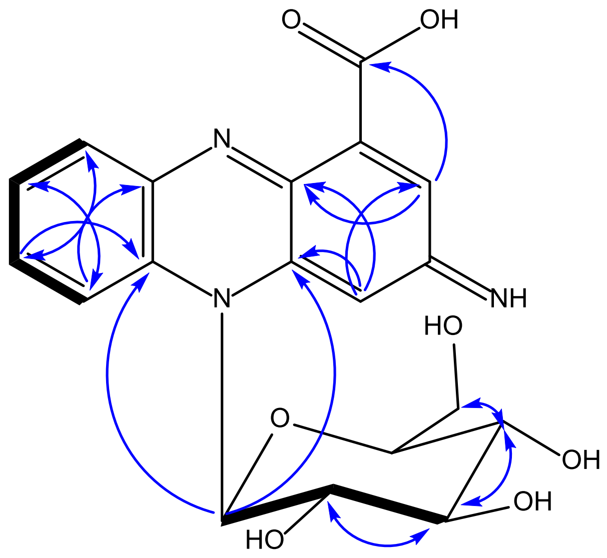

Figure S35: Structure of 3-imino-5*N*-(1′β-D-glucopyranosyl)-3,5-dihydrophenazine-1-carboxylic acid (**3**). ^{1}H-^{1}H COSY correlations are indicated by thick bonds and ^{1}H-^{13}C HMBC correlations are indicated by blue arrows.

NMR data of 3-imino-5*N*-(1′β-D-glucopyranosyl)-3,5-dihydrophenazine-1-carboxylic acid (**3**)

Table S3: ^{1}H-NMR (600 MHz, CD_3OD) data for 3-imino-5*N*-(1′β-D-glucopyranosyl)-3,5-dihydrophenazine-1-carboxylic acid (**3a** and **3b**)

**Position**	**^{1}H-NMR of 3a** shift (ppm) /multiplicity/coupling constant *J* (Hertz)	**^{13}C-NMR of 3a** shift (ppm)	**^{1}H-NMR of 3b** shift (ppm) /multiplicity/coupling constant *J* (Hertz)	**^{13}C-NMR of 3b** shift (ppm)
COOH		172.6, qC		172.6, qC
1				
2	7.35, d, 2.2	121.6 CH	7.38, d, 2.0	121.6, CH
3	-		-	
4	7.40, d, 2.2	96.7, CH	7.14, d, 2.1	93.9, CH
4a		136.8, qC		139.6, qC
5a		133.4, qC		130.8, qC
6	8.38, d, 9.0	117.3, CH	8.53, d, 9.0	120.3, CH
7	7.99, ddd, 8.7, 7.0, 1.7	136.4, CH	7.94, ddd, 8.7, 7.0, 1.7	135.2, CH
8	7.72-7.76, m[a]	127.7, CH	7.72–7.76, m [a]	128.0, CH
9	8.28, dd, 9.0, 1.7	133.6, CH	8.29, td, 17.3, 8.6, 1.6	133.9, CH
9a		139.0, qC		139.7, qC
10a		145.3, qC		144.7, qC
1'	6.58, d, 9.3	90.6, CH	6.26, d, 9.4	91.1, CH
2'	4.48, t, 8.9	69.3, CH	4.48, t, 8.9	72.2, CH
3'	3.63-4.01, m[a]	78.8, CH	3.63-4.01, m [a]	78.6, CH
4'	3.63-4.01, m[a]	70.2, CH	3.63-4.01, m [a]	70.2, CH
5'	3.63-4.01, m[a]	81.7, CH	3.63-4.01, m [a]	82.4, CH
6'	3.63-4.01, m[a]	62.1, CH_2	3.63-4.01, m [a]	62.1, CH_2

[a] overlap between signal of both isomers, range determined by ^{1}H-^{1}H-COSY cross signals.

Structure elucidation of 7-amino-phenazine-1-carboxylic acid (4)

7-Amino-phenazine-1-carboxylic acid (**4**) from purification of spent *Bacillus* sp. G2112 medium and after hydrolysis from 7-imino-5*N*-(1′β-D-glucosyl)-5,7-dihydrophenazine-1-carboxylic acid (**2**)

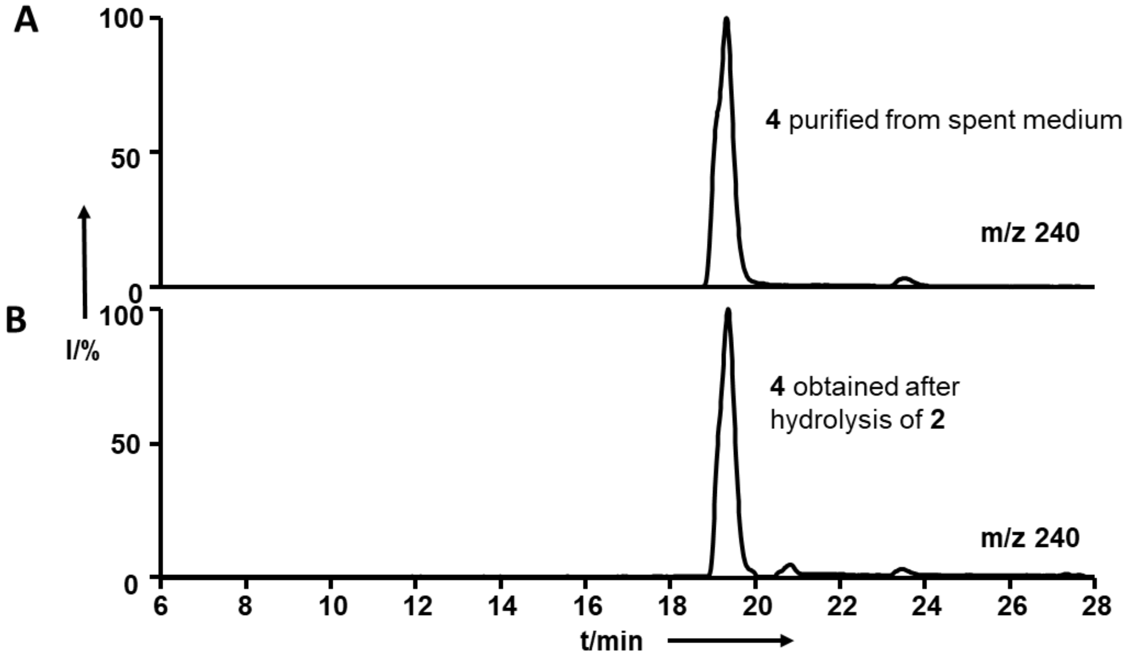

Figure S36: HPLC identification of 7- amino-phenazine-1-carboxylic acid (**4**). (A) 7-Amino-phenazine-1-carboxylic acid (**4**) obtained from purification of spent *Bacillus* sp. G2112 medium (believed to have been generated from hydrolysis of **2** during purification) and B) 7-amino-phenazine-1-carboxylic acid (**4**) after hydrolysis of purified 7-imino-5*N*-(1′β-D-glucopyranosyl)-5,7-dihydrophenazine-1-carboxylic acid (**2**)

UV-Vis spectrum of 7-amino-phenazine-1-carboxylic acid (**4**)

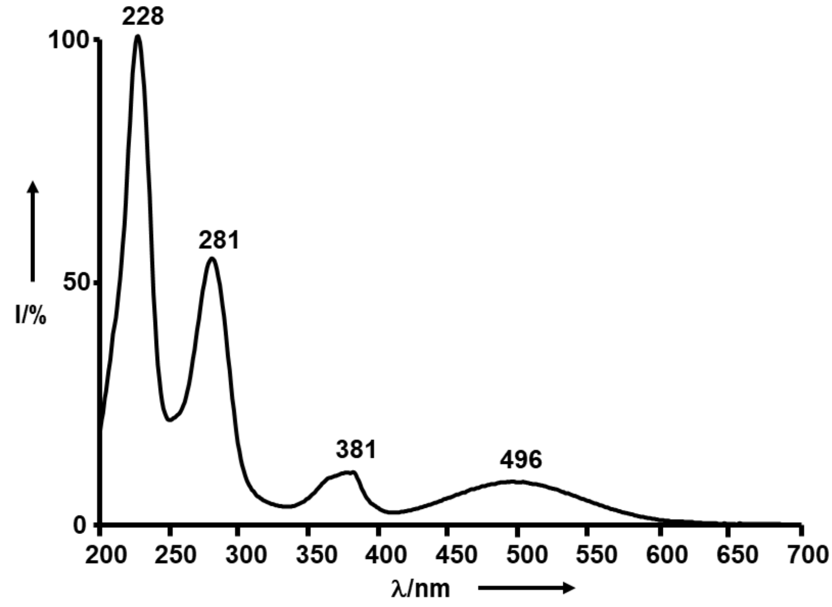

Figure S37: UV-Vis 7-amino-phenazine-1-carboxylic acid (**4**)

HR-ESI-MS of 7-amino-phenazine-1-carboxylic acid (**4**)

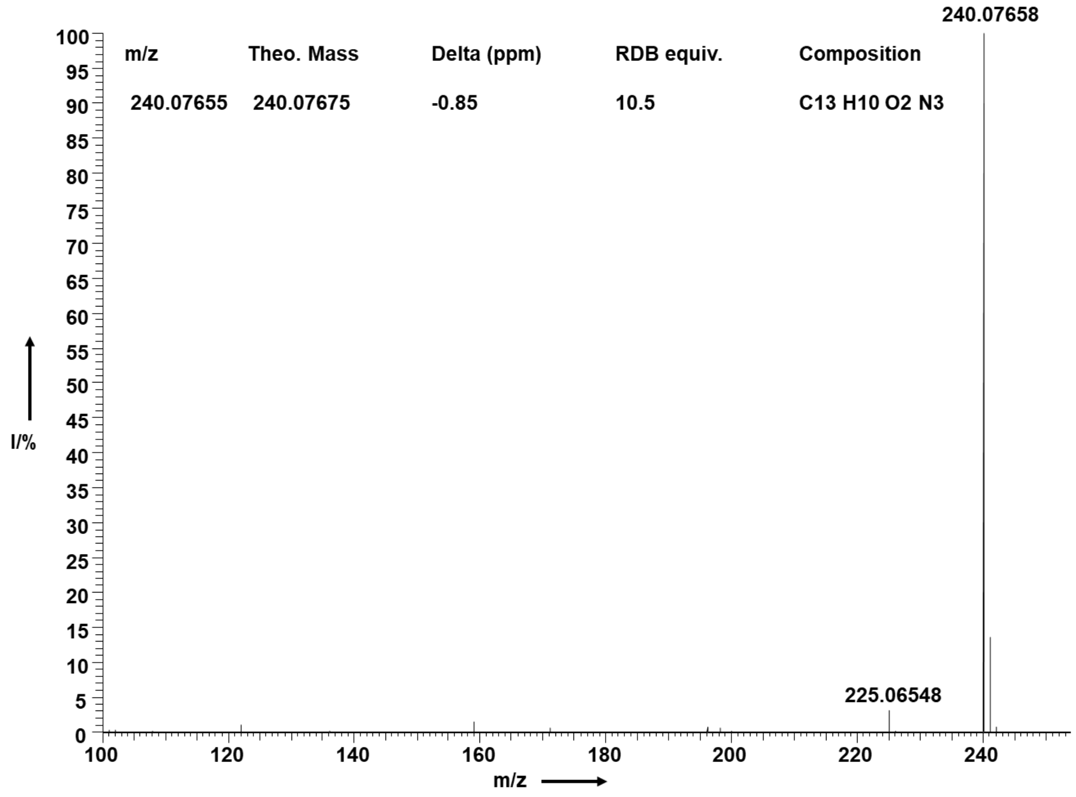

Figure S38: HR-ESI-MS of 7-amino-phenazine-1-carboxylic acid (**4**)

HR-ESI-MS/MS of 7-amino-phenazine-1-carboxylic acid (**4**)

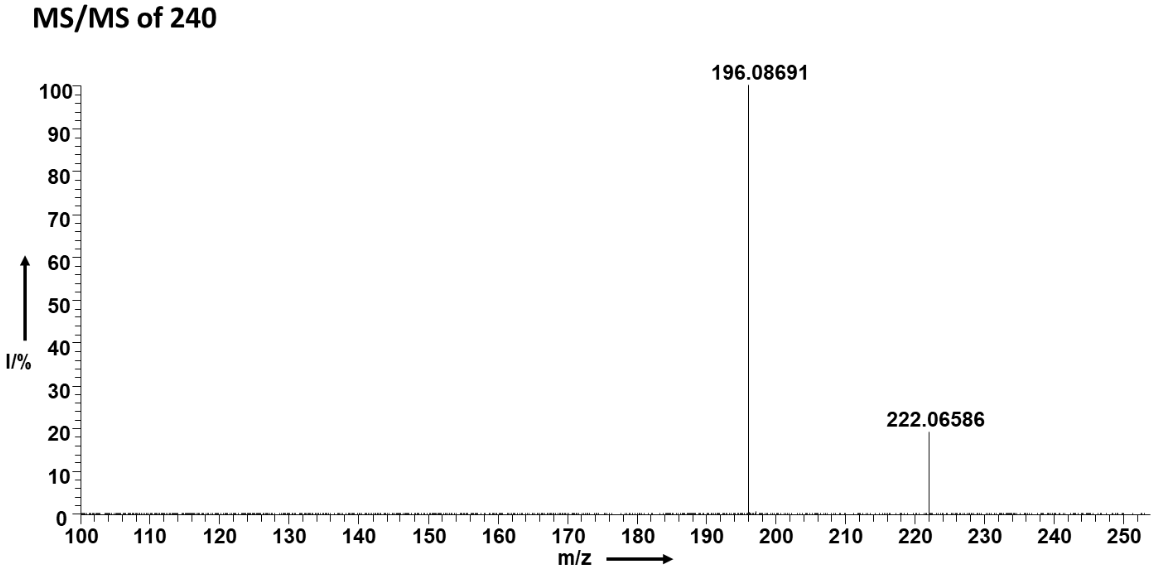

Figure S39: HR-ESI-MS/MS of 7-amino-phenazine-1-carboxylic acid (**4**)

^{1}H-NMR of 7-amino-phenazine-1-carboxylic acid (**4**) at 600 MHz in D_2O

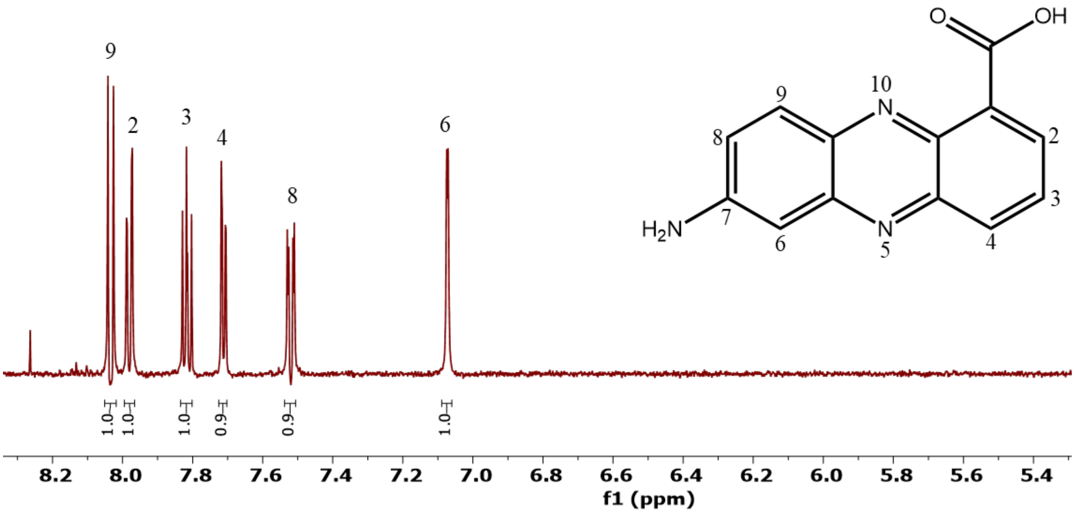

Figure S40: ^{1}H-NMR of 7-amino-phenazine-1-carboxylic acid (**4**) at 600 MHz in D_2O

^{1}H-^{1}H-COSY NMR of 7-amino-phenazine-1-carboxylic acid (**4**) at 600 MHz in D_2O

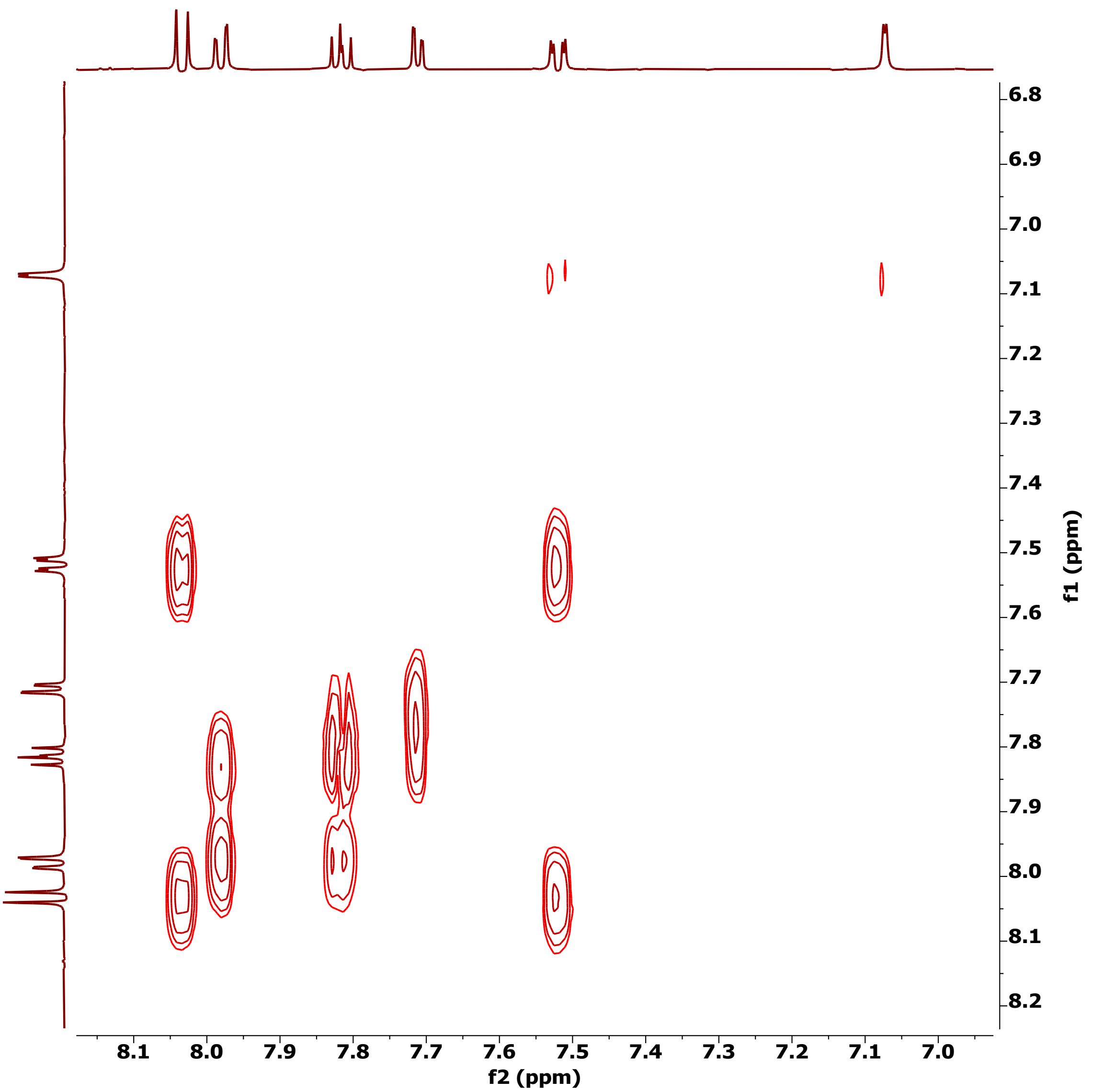

Figure S41: ^{1}H-^{1}H-COSY NMR of 7-amino-phenazine-1-carboxylic acid (**4**) at 600 MHz in D_2O

^{1}H-^{13}C-HSQC NMR 7-amino-phenazine-1-carboxylic acid (**4**) at 600 MHz in D_2O

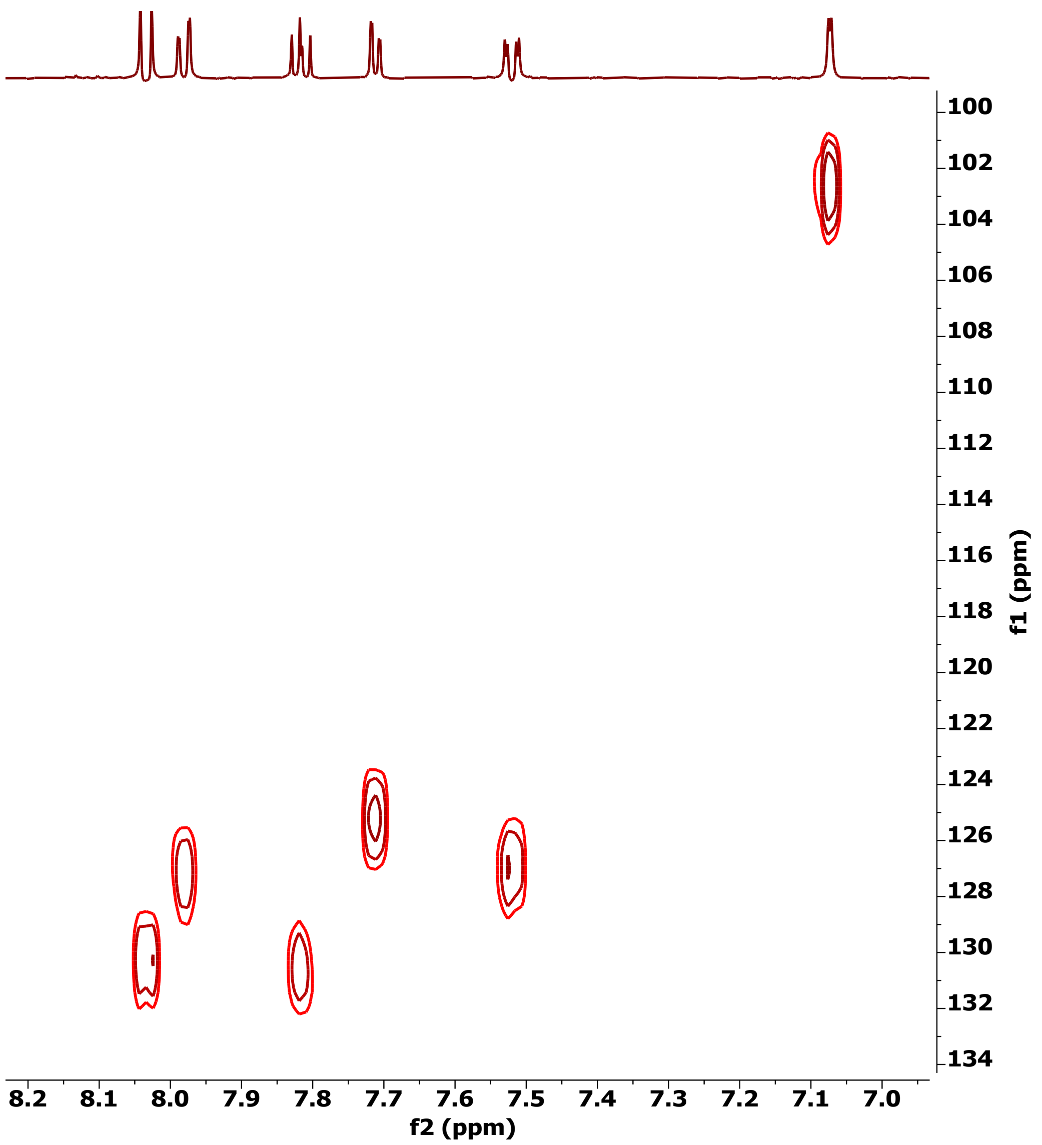

Figure S42: ^{1}H-^{13}C-HSQC NMR of 7-amino-phenazine-1-carboxylic acid (**4**) at 600 MHz in D_2O

^{1}H-^{13}C-HMBC NMR of 7-amino-phenazine-1-carboxylic acid (**4**) at 600 MHz in D_2O

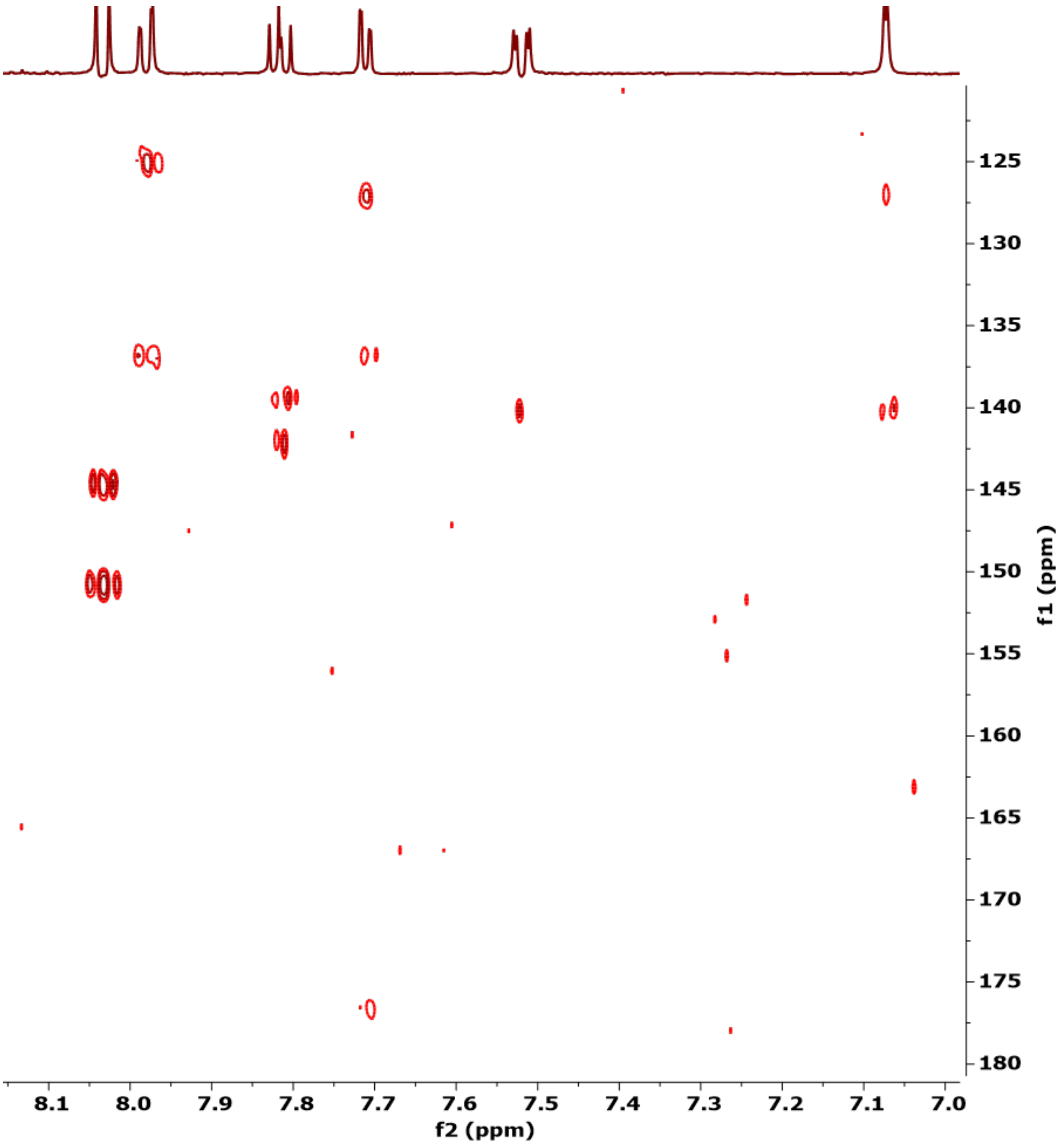

Figure S43: ^{1}H-^{13}C HMBC NMR spectrum of 7-amino-phenazine-1-carboxylic acid (**4**) at 600 MHz in D_2O

^{1}H-^{1}H COSY and ^{1}H-^{13}C HMBC correlations of 7-aminophenazine-1-carboxylic acid (**4**)

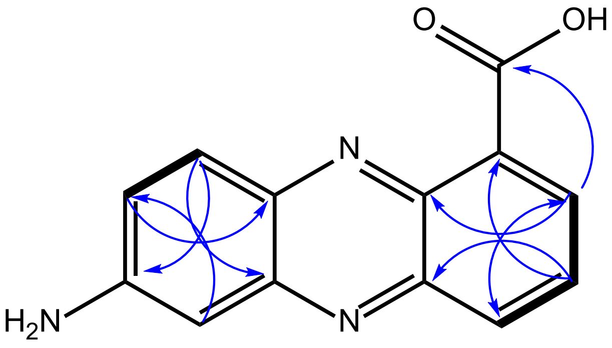

Figure S44: Structure of 7-aminophenazine-1-carboxylic acid (**4**). ^{1}H-^{1}H COSY correlations are indicated by thick bonds and ^{1}H-^{13}C-HMBC correlations are highlighted by blue arrows.

NMR data of 7-aminophenazine-1-carboxylic acid (**4**)

Table S4: ^{1}H-NMR (600 MHz, D_2O) data for 7-aminophenazine-1-carboxylic acid (**4**)

**Position**	**^{1}H-NMR of 4**<br>shift (ppm) /multiplicity/coupling constant *J* (Hertz)	**^{13}C-NMR of 4**<br>shift (ppm)
COOH	-	176.6, qC
1	-	142.0, qC
2	7.71, dd, 6.8, 1.4	125.2, CH
3	7.82, dd, 8.8, 6.8	130.5, CH
4	7.98, dd, 8.8, 1.4	127.1, CH
4a	-	139.4, qC
5a	-	144.5, qC
6	7.07, d, 2.5	102.7, CH
7	-	150.7, qC
8	7.52, dd, 9.6, 2.5	127.0, CH
9	8.03, d, 9.6	130.3, CH
9a	-	140.1, qC
10a	-	136.7, qC

Identification of the sugar moiety of imino-5*N*-(1′β-D-glucopyranosyl)-dihyrophenazine-1-carboxylic acids 2 and 3

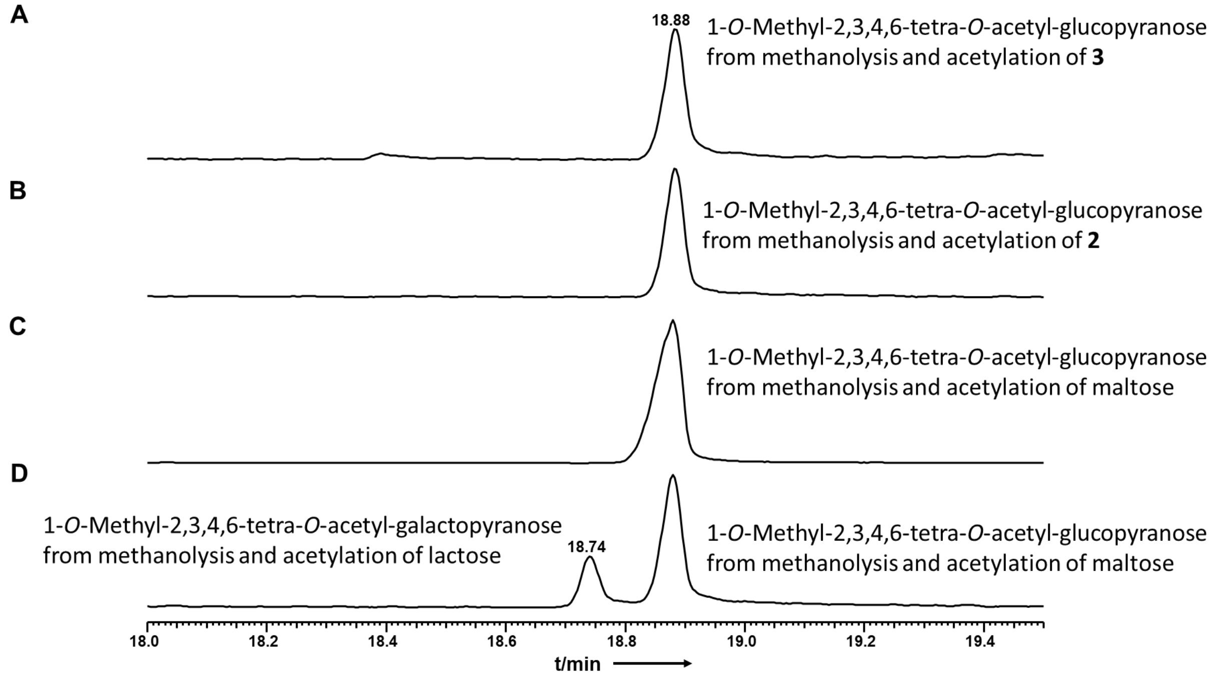

Figure S45: GC-MS identification of sugar moieties of 7-imino-5*N*-(1′β-D-glucopyranosyl)-5,7-dihydrophenazine-1-carboxylic acid (**2**) and 3-imino-5*N*-(1′β-D-glucopyranosyl)-3,5-dihydrophenazine-1-carboxylic acid (**3**).

GC-MS total ion current chromatograms of the sugar moieties after methanolysis and subsequent tetraacetylation [5] of 3-imino-5*N*-(1′β-D-glucopyranosyl)-3,5-dihydrophenazine-1-carboxylic acid (**3**) and 7-imino-5*N*-(1′β-D-glucopyranosyl)-5,7-dihydrophenazine-1-carboxylic acid (**2**) in comparison to maltose and lactose standards obtained by methanolysis and subsequent tetraacetylation. (**A**) Peak of 1-*O*-methyl-2,3,4,6-tetra-*O*-acetyl-glucopyranose obtained from degradation of **2**. (**B**) Peak of 1-*O*-methyl-2,3,4,6-tetra-*O*-acetyl-glucopyranose obtained from degradation of **3**. (**C**) Peak of 1-*O*-methyl-2,3,4,6-tetra-*O*-acetyl-glucopyranose obtained from degradation of maltose. (**D**) Peaks of 1-*O*-methyl-2,3,4,6-tetra-*O*-acetyl-galactopyranose and methyl-2,3,4,6-tetra-*O*-acetyl-glucopyranose obtained from degradation of lactose.

Determination of the stereochemistry of the sugar moiety of imino-5*N*-(1′β-D-glucopyranosyl)-dihyrophenazine-1-carboxylic acids 2 and 3

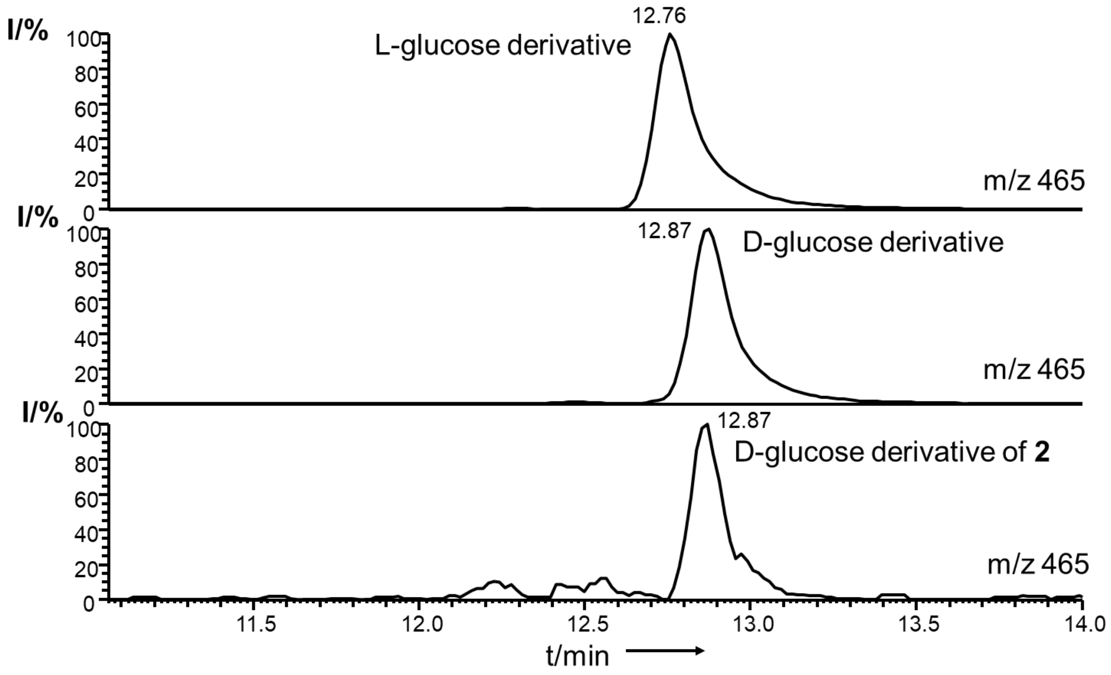

Figure S46: Determination of the stereochemistry of the glucose moiety of 7-imino-5*N*-(1′β-D-glucopyranosyl)-5,7-dihydrophenazine-1-carboxylic acid (**2**). LC-MS ion traces of the quasimolecular ion m/z 465 of the glucose derivative. Comparison to L-glucose and D-glucose standards to the glucose obtained after acid hydrolysis and derivatisation with L-cysteine methyl ester and 4-fluorobenzyl isothiocyanate of **2** [6].

Atropisomers of the imino-5*N*-(1′β-D-glucopyranosyl)-dihydrophenazine-1-carboxylic acids 2 and 3

NOESY NMR spectrum of 7-imino-5*N*-(1′β-D-glucopyranosyl)-5,7-dihydrophenazine-1-carboxylic acid (**2**)

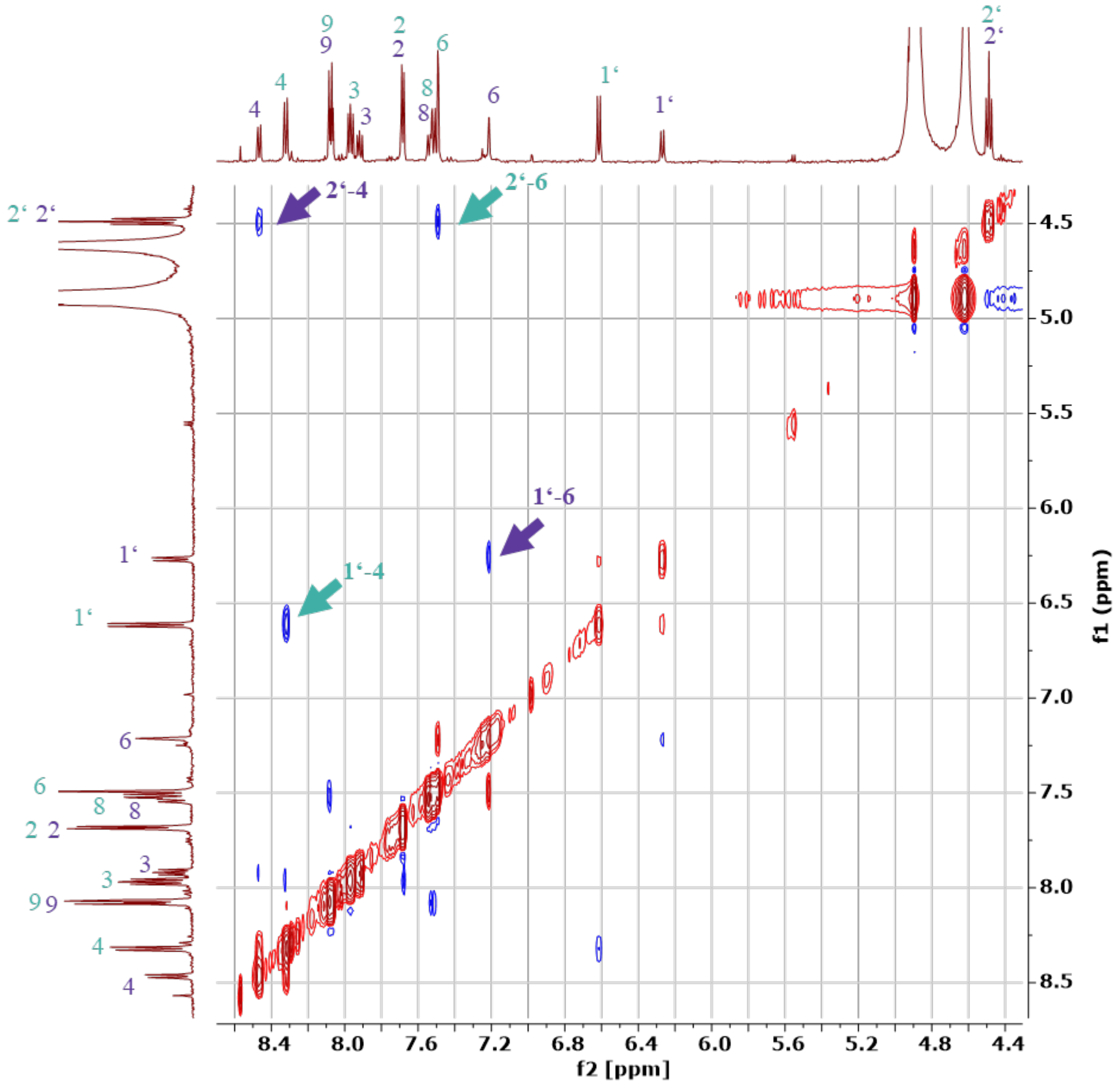

Figure S47: NOESY NMR spectrum of 7-imino-5*N*-(1′β-D-glucopyranosyl)-5,7-dihydrophenazine-1-carboxylic acid (**2**) and structures of its atropisomers **2a** and **2b.** The relevant NOESY correlations are highlighted by arrows. Atom numbers of the major isomer **2a** are shown in green and atom numbers of the minor isomer **2b** in purple.

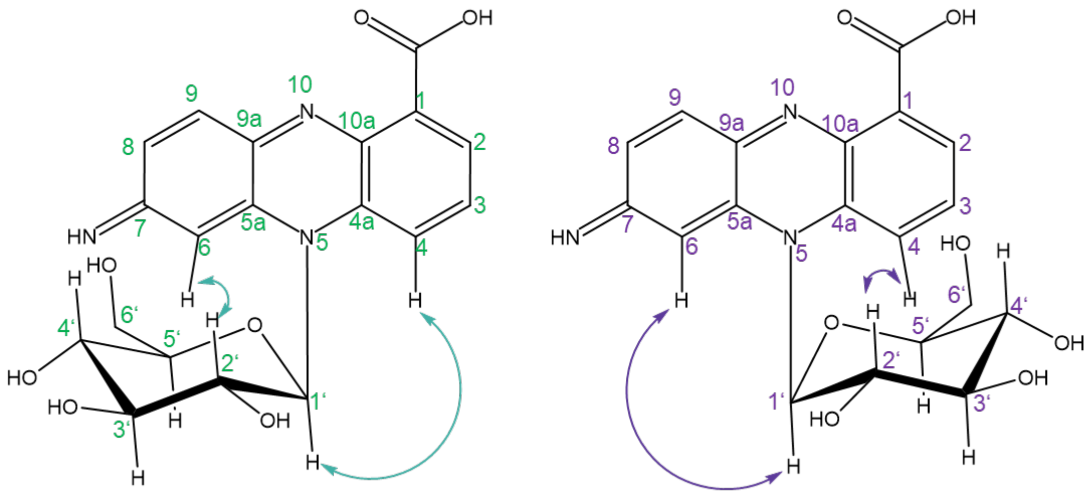

2a **2b**

Figure S48: Suggested 3-dimensional structures of the atropisomers of 7-imino-5*N*-(1′β-D-glucopyranosyl)-5,7-dihydrophenazine-1-carboxylic acid (**2a** and **2b**) fitting to the NOESY NMR correlations: **2a** exhibits NOESY correlations between H1′ and H4 and between H2′ and H6. **2b** exhibits NOESY correlations between H1′ and H6 and between H2′ and H4. Moreover, the 5*N* nitrogen of **2** is chiral due to its lone electron pair. Only one of the possible stereoisomers is depicted. It was not possible for us to address the stereochemistry at *N*5 with the current experiments.

NOESY NMR spectrum of 3-imino-5*N*-(1′β-D-glucopyranosyl)-3,5-dihydrophenazine-1-carboxylic acid (**3**)

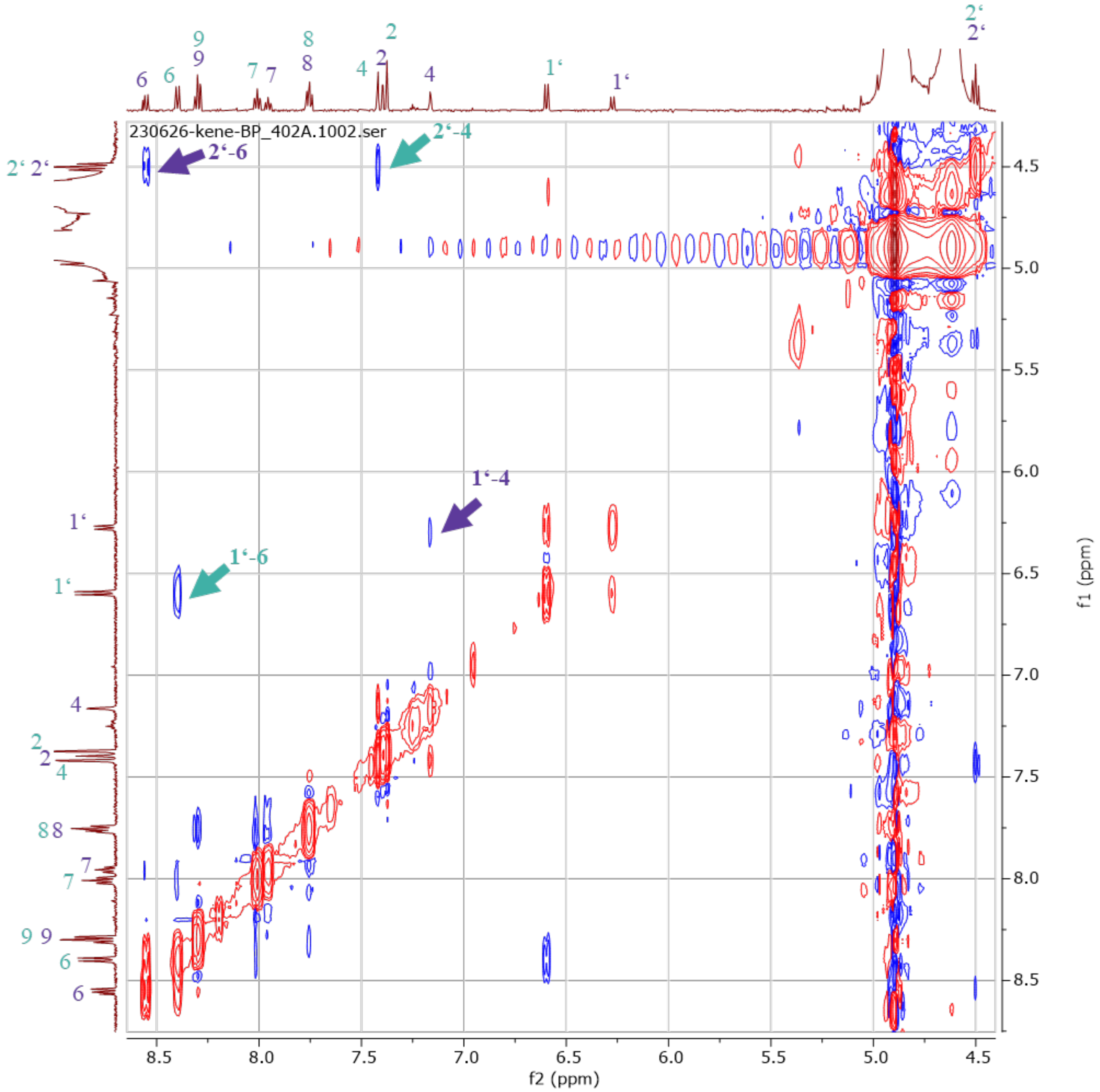

Figure S49: NOESY NMR spectrum of 3-imino-5*N*-(1′β-D-glucopyranosyl)-3,5-dihydrophenazine-1-carboxylic acid (**3**) and structures of its atropisomers **3a** and **3b**. The relevant NOESY correlations are highlighted by arrows. Atom numbers of the major isomer (**3a**) are shown in green and atom numbers of the minor isomer (**3b**) in purple.

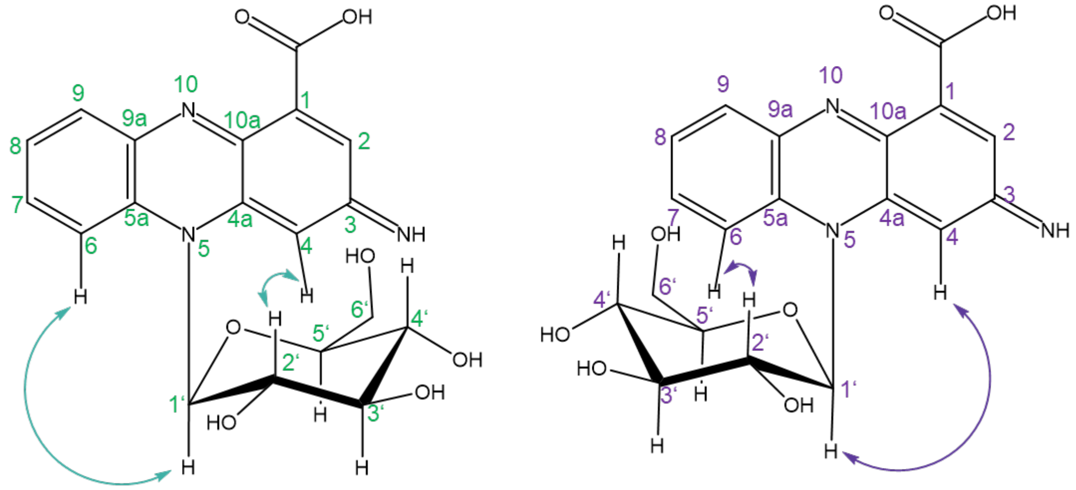

3a **3b**

Figure S50: Suggested 3-dimensional structures of the atropisomers of 3-imino-5*N*-(1′β-D-glucopyranosyl)-3,5-dihydrophenazine-1-carboxylic acid (**3a** and **3b**) fitting to the NOESY-NMR correlations. **3a** exhibits NOESY correlations between H1′ and H6 and between H2′ and H4. **3b** exhibits NOESY correlations between H1′ and H4 and between H2′ and H6. Moreover, the 5*N* nitrogen of **3** is chiral due to its lone electron pair. Only one of the possible stereoisomers is depicted. It was not possible for us to address the stereochemistry at *N*5 with the current experiments.

Toxicity assays of phenazine-1-carboxylic acid (1) and 3-imino-5*N*-(1′β-D-glucopyranosyl)-3,5-dihydrophenazine-1-carboxylic acid (3)

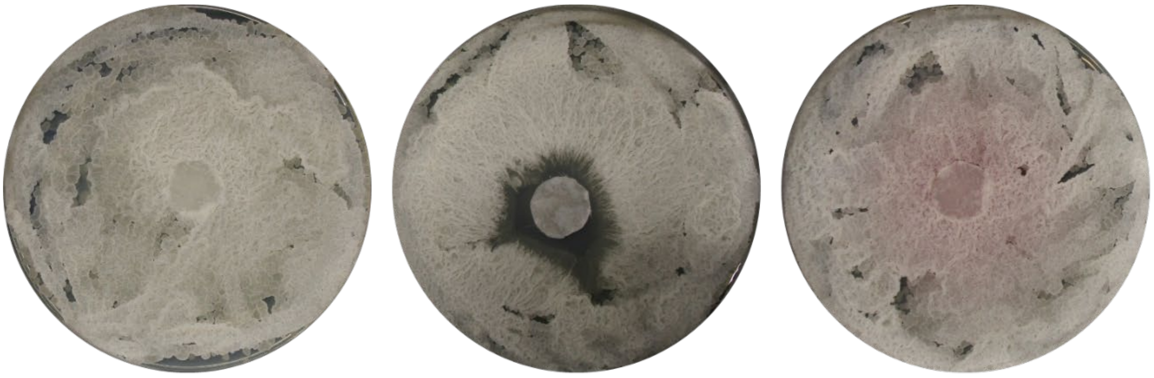

Control (MeOH) | PCA (**1**) | 3-imino-5*N*-(1’β-D-glucopyranosyl)-3,5-dihydrophenazine-1-carboxylic acid (**3**)

Figure S51: Agar diffusion bioassays with PCA (**1**) and 3-imino-5*N*-(1′β-D-glucopyranosyl)-3,5-dihydrophenazine-1-carboxylic acid (**3**) against *Bacillus* sp. G2112. 20 µL of 20 mM PCA (**1**) inhibited the growth of *Bacillus* sp. G2112, whereas 20 µL of 20 mM 3-imino-5*N*-(1′β-D-glucopyranosyl)-3,5-dihydrophenazine-1-carboxylic acid (**3**) did not inhibit the growth of *Bacillus* sp. G2112.

Structures of all known glycosylated phenazines

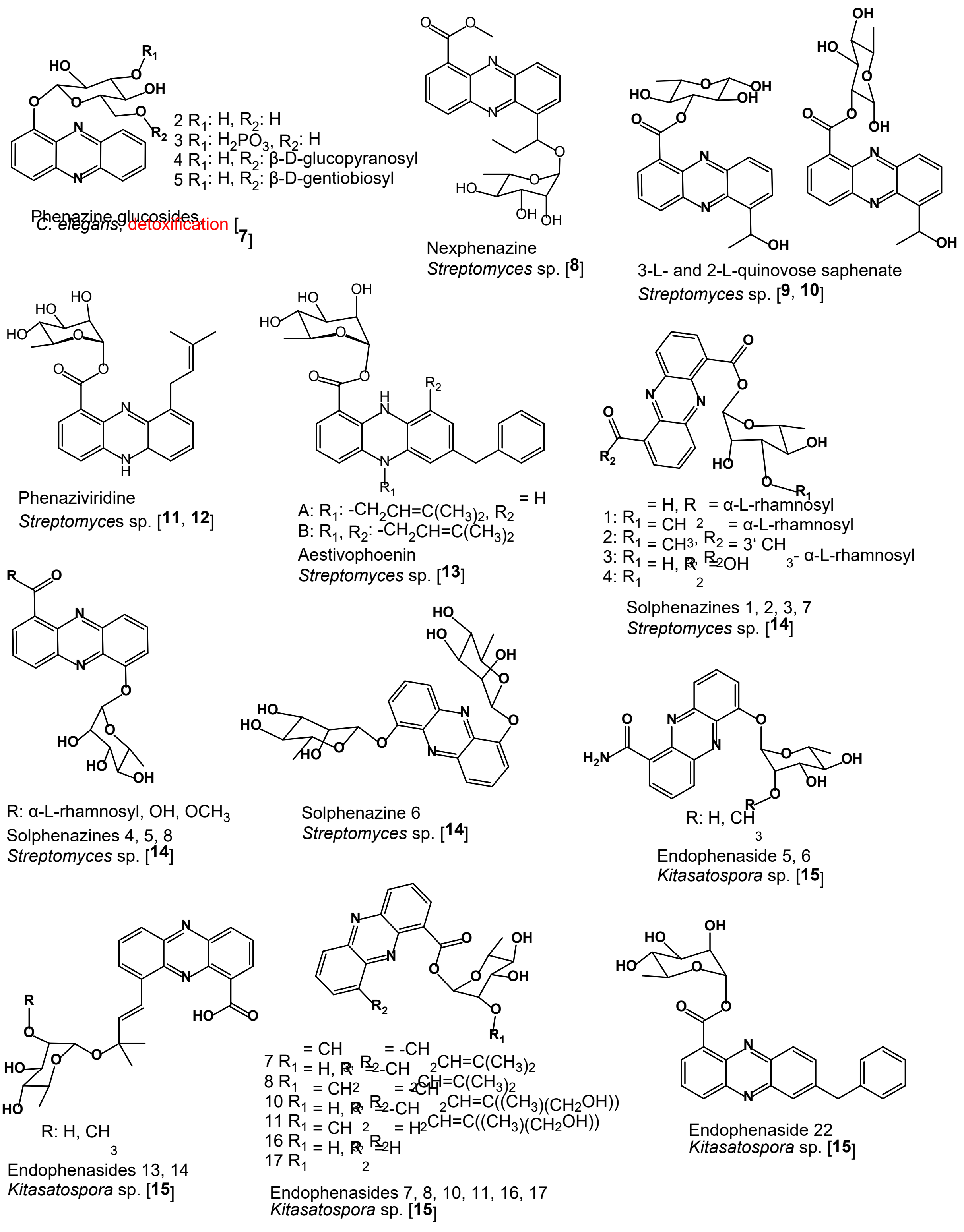

Figure S52: Structures of known glycosylated phenazines. Except for one group of phenazine *O*-glycoside (highlighted red) which are detoxification products of 1-phenazinol from *C. elegans* [7], all compounds constitute secondary metabolites produced by *Streptomyces* [8–14] or *Kitasatospora* [15] strains. No 5*N* functionalized glycosides have been reported.

References

1. Saitou, N.; Nei, M. The neighbor-joining method: A new method for reconstructing phylogenetic trees. *Mol. Biol. Evol.* **1987**, *4*, 406–425. https://doi.org/10.1093/oxfordjournals.molbev.a040454.
2. Edgar, R. C. MUSCLE: Multiple sequence alignment with high accuracy and high throughput. *Nucleic Acids Res.* **2004**, *32*, 1792–1797. https://doi.org/10.1093/nar/gkh340.
3. Felsenstein, J. Confidence Limits on Phylogenies: An approach using the bootstrap. *Evolution (N. Y).* **1985**, *39*, 783–791. https://doi.org/10.2307/2408678.
4. Tamura, K.; Stecher, G.; Kumar, S. MEGA11: Molecular evolutionary genetics analysis version 11. *Mol. Biol. Evol.* **2021**, *38*, 3022–3027. https://doi.org/10.1093/molbev/msab120.
5. Dasgupta, F.; Singh, P. P.; Srivastava, H. C. Acetylation of carbohydrates using ferric chloride in acetic anhydride. *Carbohydr. Res.* **1980**, *80*, 346–349. https://doi.org/10.1016/S0008-6215(00)84876-4.
6. Tanaka, T.; Nakashima, T.; Ueda, T.; Tomii, K.; Kouno, I. Facile discrimination of aldose enantiomers by reversed-phase HPLC. *Chem. Pharm. Bull.* **2007**, *55*, 899–901. https://doi.org/10.1248/cpb.55.899.
7. Stupp, G. S.; Von Reuss, S. H.; Izrayelit, Y.; Ajredini, R.; Schroeder, F. C.; Edison, A. S. Chemical detoxification of small molecules by *Caenorhabditis elegans*. *ACS Chem. Biol.* **2013**, *8*, 309–313. https://doi.org/10.1021/cb300520u.
8. Wang, Z.; Yang, F. X.; Liu, C.; Wang, L.; Qi, Y.; Cao, M.; Guo, X.; Li, J.; Huang, X.; Yang, J.; Huang, S. X. Isolation and biosynthesis of phenazine-polyketide hybrids from *Streptomyces* sp. KIB-H483. *J. Nat. Prod.* **2022**, *85*, 1324–1331. https://doi.org/10.1021/acs.jnatprod.2c00067.
9. Laursen, J. B.; Petersen, L.; Jensen, K. J.; Nielsen, J. Efficient synthesis of glycosylated phenazine natural products and analogs with DISAL (methyl 3,5-dinitrosalicylate) glycosyl donors. *Org. Biomol. Chem.* **2003**, *1*, 3147–3153. https://doi.org/10.1039/b306789k.
10. Pathirana, C.; Jensen, P. R.; Dwight, R.; Fenical, W. Rare phenazine l-quinovose esters from a marine actinomycete. *J. Org. Chem.* **1992**, *57*, 740–742.
11. Krastel, P.; Zeeck, A. Endophenazines A-D, new phenazine antibiotics from the athropod associated endosymbiont *Streptomyces anulatus*. *J. Antibiot.* **2002**, *55*, 801–806.
12. Kato, S.; Shindo, K.; Yamagishi, Y.; Matsuoka, M.; Kawai, H.; Mochizuki, J. Phenazoviridin, a novel free radical scavenger from *Streptomyces* sp. Taxonomy, fermentation, isolation, structure elucidation and biological properties. *J. Antibiot.* **1993**, *46*, 1485–1493.
13. Shin-Ya, K.; Shimizu, S.; Kunigami, T.; Hayakawa, Y.; Seto, H.; Furihata, K. Novel neuronal cell protecting substances, aestivophoenins A and B, produced by *Streptomyces purpeofuscus*. *J. Antibiot.* **1995**, *48*, 1378–1381. https://doi.org/10.7164/antibiotics.48.1378.
14. Rusman, Y.; Oppegard, L. M.; Hiasa, H.; Gelbmann, C.; Salomon, C. E. Solphenazines A-F, glycosylated phenazines from *Streptomyces* sp. strain DL-93. *J. Nat. Prod.* **2013**, *76*, 91–96. https://doi.org/10.1021/np3007606.
15. Wu, C.; Medema, M. H.; Läkamp, R. M.; Zhang, L.; Dorrestein, P. C.; Choi, Y. H.; Van Wezel, G. P. Leucanicidin and endophenasides result from methyl-rhamnosylation by the same tailoring enzymes in *Kitasatospora* sp. MBT66. *ACS Chem. Biol.* **2016**, *11*, 478–490. https://doi.org/10.1021/acschembio.5b00801.
